# Characterization and quantitation of urinary metabolites of 3-monochloropropane-1,2-diol (3-MCPD) in rats

**DOI:** 10.1007/s00204-026-04318-x

**Published:** 2026-03-10

**Authors:** Thorsten Henning, Cornelius Goerdeler, Ahmed H. El-Khatib, Klas Meyer, Gustav G. Bruer, Klaus Abraham, Bernhard H. Monien

**Affiliations:** 1 German Federal Institute for Risk Assessment (BfR), Food and Feed Safety in the Food Chain, Max-Dohrn-Strasse 8-10, 10589 Berlin, Germany; 2 German Federal Institute for Risk Assessment (BfR), Reference Center Food and Feed Analysis, 10589 Berlin, Germany; 3 Institute of Legal Medicine, University Hospital Essen, Tüschener Weg 44, 45239 Essen, Germany; 4 Analytical Chemistry, Federal Institute for Materials Research and Testing (BAM), 12489 Berlin, Germany; 5 Fraunhofer Institute for Toxicology and Experimental Medicine (ITEM), 30625 Hannover, Germany

**Keywords:** 3-Monochloropropane-1,2-diol, 3-MCPD, Metabolism, Urine, Thiodiglycolic acid

## Abstract

Fatty acid esters of 3-monochloropropane-1,2-diol (3-MCPD) are heat-induced contaminants formed from fats and sodium chloride. The mode of action for the 3-MCPD mediated induction of renal tubule neoplasms in rats is still unclear, which is in part due to lacking metabolism data. In the current study, urinary metabolites were identified by one- and two-dimensional ^13^C nuclear magnetic resonance spectroscopy and high-resolution mass spectrometry following oral administration of 3-MCPD or [^13^C_3_]3-MCPD in rats. In addition to 3-MCPD itself, nine metabolites were identified. Four of those, *N*-acetyl-S-(2,3-dihydroxypropyl)cysteine (DHPMA), 3-MCPD sulfate, β-chlorolactic acid (β-ClLA) and oxalic acid have been reported before. Five novel metabolites in rat urine were thiodiglycolic acid (TDGA), thionyldiglycolic acid (TNDGA), 3-MCPD glucuronide (at least three isomers), 3-carboxy-2-hydroxypropyl mercapturic acid (CHPMA), and 3-(S-carboxymethyl)mercaptolactic acid (CMMLA). Only three metabolites were excreted at mean dose ratios > 1% (after treatment with 50 mg 3-MCPD/kg body weight in male and female rats), i.e. 3-MCPD (7.3%, 9.7%), DHPMA (3.5%, 1.7%) and TDGA (1.1%, 4.0%). The overall mean dose excretion in urine samples (males: 12.2%, females: 16.3%) supported hypotheses on formation of conjugates/adducts of reactive metabolites and the possibility that dechlorination leads to suitable building blocks for amino acid and fatty acid synthesis. The identification of TDGA indicated the interim formation 2-chloroacetaldehyde, previously identified to mediate specific nephrotoxic effects of the cytostatic ifosfamide in rats.

**Supplementary Information:**

The online version contains supplementary material available at 10.1007/s00204-026-04318-x.

## Introduction

Fatty acid esters of 3-MCPD are heat-induced contaminants particularly formed as by-products in the refinement process of vegetable and animal oils with highest amounts being quantified in palm oil/fat (Beekmann et al. 2022; Weißhaar 2011). Used as ingredient in various foods, contaminated palm oil is a frequent cause for elevated contents of 3-MCPD esters found in, e.g., baked goods or infant formula. Upon intake, 3-MCPD esters are hydrolyzed rapidly in the gastrointestinal tract, resulting in the release and subsequent absorption of free 3-MCPD (Abraham et al. 2013).

3-MCPD has been classified by the International Agency for Research on Cancer (IARC) as possibly carcinogenic to humans (IARC 2013). Short term exposure of 3-MCPD above 1 mg/kg body weight (bw) was shown to decrease sperm motility in male rats (Takayama et al. 1995). Additionally, development of nephropathy and renal tubular hyperplasia were observed in male rats after chronic daily exposure to 2 mg 3-MCPD/kg bw (Cho et al. 2008). A benchmark dose level (BMDL_10_) of 0.20 mg/kg bw per day was estimated and the European Food Safety Authority (EFSA) derived a group tolerable daily intake (TDI) for 3-MCPD and its fatty acid esters of 2 µg/kg bw (EFSA 2018).

The average exposure to 3-MCPD in European countries was estimated to be in the range of 0.5–1.5 μg/kg bw per day for infants, toddlers, and children up to 10 years old and between 0.2 and 0.7 μg/kg bw per day for adolescents and adults (EFSA 2016). An exposure assessment conducted by the German Federal Institute for Risk Assessment (BfR) showed that the TDI is not exceeded in an adult population even if high consumption of 3-MCPD containing foods is considered (0.5–0.6 µg/kg bw per day). In contrast, the estimated intake of non-breastfed infants (3.1 µg/kg bw per day) exceeded the TDI assuming exclusive consumption of infant formula (German Federal Institute for Risk Assessment 2020). In order to ensure an optimal protection, the maximum levels for the sum of free and ester-bound 3-MCPD in infant formulae, follow-on formulae, food for special medical purposes intended for infants and young children were reduced by the European Commission to 80 µg/kg for powdered and to 12 µg/kg for liquid preparations in 2025 (European Commission 2024).

The metabolism of 3-MCPD has not been well characterized. In rats, detoxification occurs via two routes. The oxidative conversion, presumably to β-chlorolactaldehyde, leads to the formation of β-chlorolactic acid (β-ClLA), and subsequently to oxalic acid (Jones and Murcott 1976) and CO_2_ (Jones 1975). The conjugation with glutathione results in the formation of 2,3-dihydroxypropyl mercapturic acid (DHPMA) (Jones 1975). Limited quantitative data are available for rats and humans. The intraperitoneal injection (i.p.) of 100 mg/kg bw [^14^C]3-MCPD in rats revealed that about 30% of the dose was exhaled as [^14^C]CO_2_ (Jones 1975). In another experiment, i.p. injection of 100 mg/kg bw [^36^Cl]3-MCPD showed that 16% of the radioactive labelled chlorine was excreted as Cl^−^ and 8.5% was excreted unmetabolized (Jones et al. 1978; Jones and Murcott 1976). After oral administration of 29.5 mg/kg bw 3-MCPD in rats, Barocelli et al. (2011) recovered 0.9/1.7% (females/males) of the original dose as β-ClLA, 7.3/12.5% as DHPMA, and 5.8/2.6% as unmetabolized 3-MPCD within 24 h in collected urine samples. In a human study, oral controlled exposure to 12 g MCPD-rich hazelnut oil (content 54.5 mg 3-MCPD/kg) in twelve healthy non-smokers led to the mean urinary excretion of free 3-MCPD (3.7%), and β-ClLA (0.28%). In contrast to rats, however, DHPMA was only excreted at around 1%, observed in a single person after oral uptake of 10 mg 3-MCPD (Abraham et al. 2021; Bergau et al. 2021). Taken together, only small parts of 3-MCPD metabolism in rats has been clarified and the gap in human data is even larger. Furthermore, there is a lack of evidence for metabolites of 3-MCPD that could explain the tissue-specific toxicity of 3-MCPD observed, e.g., in the renal and reproductive system (Eisenreich et al. 2023).

The primary goal of the current study was to complete the knowledge on 3-MCPD metabolism. In accordance to most previous studies, rats were used as model organism. Single high doses of 3-MCPD (50 or 5 mg/kg bw) and [^13^C_3_]3-MCPD (50 mg/kg bw) were administered in male and female Wistar rats. The application of 3-MCPD and the isotope-labelled compound ensures a particular specificity of identification with data obtained from high-resolution mass spectrometry (HRMS) and nuclear magnetic resonance (NMR) spectroscopy.

## Materials and methods

### Chemicals

2,2’-Thiodiglycolic acid (TDGA), S-carboxymethyl-L-cysteine and chloroacetic acid were purchased from VWR (Darmstadt, Germany). Phenylboronic acid (≥ 97%), diethyl ether (analytical grade), ethyl acetate (analytical grade), tert-butyl methyl ether (tBME, analytical grade), iso-hexane (analytical grade), iso-octane (analytical grade), methanol (analytical grade), sodium bromide (Ph. Eur.), sodium sulfate (anhydrous, granulated, for organic trace analysis) and formic acid were purchased from Merck KGaA (Darmstadt, Germany). HPLC-grade water was prepared using a Milli-Q Integral Water Purification System from Millipore Merck. Sodium sulfate was dried overnight in a muffle furnace at approx. 200 °C before use. ( ±)-3-Chloro-1,2-propanediol (3-MCPD) was from Fisher Scientific (Schwerte, Germany). [d_5_]3-MCPD was obtained from Sigma-Aldrich (Steinheim, Germany). [d_4_]Thiodiglyolic acid, [^13^C_2_]oxalic acid and DHPMA (N-acetyl-S-(2,3-dihydroxypropyl)-L-cysteine) and its corresponding internal standard [d_5_]DHPMA were purchased by Toronto Research Chemicals (North York, Canada). The main of two isomers of 3-MCPD sulfate (3-chloro-2-hydroxypropyl hydrogen sulfate) was synthetized by WITEGA Laboratories GmbH (Berlin, Germany). The compound [^13^C_3_]β-ClLA was synthesized by ASCA GmbH (Berlin, Germany). Synthesis pathways (if disclosed by the companies) and certificates of analysis are included in the Supplemental Information.

### Animal experiment

24 Wistar rats (12 male, 12 female, eight weeks old) were purchased by Charles River Laboratory (Sulzfeld, Germany) and distributed into four groups (n = 3 per group, male and female). Prior to the treatment, animals were allowed to acclimatize for two weeks. Rats were administered single oral doses by gavage of either 3-MCPD (50 or 5 mg/kg bw) or [^13^C_3_]3-MCPD (50 mg/kg bw) dissolved in 10% ethanol (6.25 ml/kg bw) or the vehicle solvent without detectable amounts of 3-MCPD (control groups). Animals were held in metabolic cages to collect complete urine samples between 0–8 h, 8–24 h and 24–48 h. Afterwards rats were euthanized by intraperitoneal injection of 160 mg pentobarbital. Ethical approval was provided by the Lower Saxony State Office for Consumer Protection and Food Safety (LAVES), file number 33.19–42,502-04–22-00249 (study-IDs 02 N 22 542 and 02 N 24 508).

### Mass spectrometric quantification of urinary metabolites

#### 3-MCPD by gas chromatography-mass spectrometry (GC–MS)

Derivatization and GC–MS analysis were performed similarly as described before (Abraham et al. 2021). Briefly, urine samples (4 mL) were spiked with 40 µL [d_5_]3-MCPD internal standard (1 mg/L in methanol). Matrix was depleted with 3.5 mL tBME/iso-hexane (1:4), and 3-MCPD was extracted three times with 2.5 mL diethyl ether/ethyl acetate (9:1). After drying over sodium sulfate and derivatization with 50 µL phenylboronic acid (2 mg/L) in diethyl ether/ethyl acetate (9:1), samples were concentrated under nitrogen flow. The residuals were resuspended in 150 µL iso-octane and 2 µL injected by pulsed splitless injection onto an 8890 GC (Agilent, Waldbronn, Germany) equipped with a 5977B mass selective detector (Agilent). Separation was performed on an Restek Rxi-17 column (30 m × 0.25 mm ID, 0.25 µm film consisting of 50% diphenyl/50% dimethylpolysiloxane) using the following temperature gradient: 80 °C (isothermal for 2 min), 5.4 °C/min to 150 °C (isothermal for 4 min), 20 °C/min to 280 °C (isothermal for 5 min), cool-down and re-equilibration (3 min). Analytes were ionized by electron impact and operated in single ion monitoring using the detailed MS parameters as described previously. Recorded mass-to-charge ratios (*m/z*) of the phenylboronic derivatives of 3-MCPD were 147, 196, 198 (3-MCPD) and 150, 201, 203 ([d_5_]3-MCPD). The *m/z* ratio 147/150 was used for the quantification of 3-MCPD.

#### β-ClLA by ion-pair liquid chromatography (IPC)-MS/MS

The method was described by Bergau et al. (2021). Briefly, aliquots of urine samples (20 µL) were diluted with 80 µL of internal standard solution (100 ng/mL [^13^C_3_]β-ClLA) and centrifuged at 18,000 × g for 10 min. Eight µL of the supernatants were injected into the IPC system consisting of an 1100 HPLC (Agilent) equipped with a Nucleoshell RP 18 plus column (2.0 × 150 mm, 2.7 µm; Macherey–Nagel, Düren, Germany). Gradient elution was performed using water containing 10 mM tributylamine and 0.06% acetic acid (eluent A) and acetonitrile (eluent B). The flow rate of the gradient (0–3 min, 2% B; 3–6.5 min, 2–36% B; 6.5–8 min, 36–95% B; 8–10.5 min, 95% B; 10.5–15 min, 2% B) was 0.5 mL/min. The column oven was set to 40 °C. β-ClLA was detected with a QTrap 6500 (Sciex, Darmstadt, Germany) operated in the negative ionization mode using multiple reaction monitoring (MRM). The analyte was quantified by the peak ratio of the transitions *m/z* 123 → 35 (β-ClLA) and *m/z* 126 → 35 ([^13^C_3_]β-ClLA). Further details can be found elsewhere (Bergau et al. 2021).

#### TDGA, 3-MCPD sulfate and [^13^C_2_]oxalic acid by IPC-MS/MS

TDGA and 3-MCPD sulfate were analyzed simultaneously. TDGA was determined by isotope-dilution IPC-MS/MS, and 3-MCPD sulfate was quantified at hand of an external calibration line. Aliquots of urine samples (20 µL) were spiked with 10 µL [d_4_]TDGA solution (100 µg/mL in water) and diluted with 370 µL eluent A (see below). Twelve mixtures containing 20 µL of pooled rat urine of six control animals (devoid of 3-MCPD sulfate) and between 0.01 and 50 µM 3-MCPD sulfate were prepared for the external calibration line and were otherwise treated as normal samples. The samples were centrifuged at 18,000 × g for 10 min and 10 µL of the supernatants were injected. The IPC was performed on an 1100 HPLC (Agilent) using a Nucleoshell RP18 plus column (2.0 × 150 mm, 2.7 µm, Macherey–Nagel) heated to 45 °C. The analytes were eluted using 10 mM tributylamine and 0.25% acetic acid (eluent A) and acetonitrile (eluent B). The flow rate of the gradient (0–3 min, 2% B; 3–4 min, 2–25% B; 4–6 min, 25% B; 6–8 min, 25–95% B; 8–10 min, 95% B; 10–10.1 min, 2% B; 10.1–15 min, 2% B) was 0.5 mL/min. TDGA and 3-MCPD sulfate were detected on a QTrap 6500 (Sciex). The MRM transitions for the quantifications were *m/z* 149.0→104.8 (TDGA), *m/z* 152.9→108.8 ([d_4_]TDGA), and *m/z* 188.9→152.7 (3-MCPD sulfate). Further details on sample preparation and mass spectrometric analyses of TDGA and 3-MCPD sulfate are summarized in the Supplemental Information. In a similar fashion, [^13^C_2_]oxalic acid was quantified by IPC-MS/MS using an Acquity I-Class UHPLC (Waters, Eschborn, Germany) and a QTrap 6500 (Sciex). Details are summarized in the Supplemental Information. Exemplary chromatograms of 3-MCPD sulfate (Figure S1), TDGA (Figure S2) and [^13^C_2_]oxalic acid (Figure S3) as well as data on the linearity of detection (Figure S4) are shown in the Supplemental Information.

#### DHPMA by ultra-high performance liquid chromatography-tandem mass spectrometry (UHPLC-MS/MS)

Aliquots of 20 µL urine were spiked with 80 µL of internal standard solution (10 ng/mL [d_5_]DHPMA in 0.1% formic acid) and centrifuged at 18,000 × g for 10 min. Five µL of sample supernatants were subjected to chromatographic separation using an Acquity I-Class UHPLC (Waters) equipped with an HSS T3 column (2.1 × 100 mm, 1.8 µm, Waters). Gradient elution was performed using 0.1% formic acid (eluent A) and 0.1% formic acid in acetonitrile (eluent B). The gradient was: 0–1 min, 2% B; 1–3 min, 2–20% B; 3.01–4 min, 95% B; 4.01–5 min, 2% B. The flow rate was 0.4 mL/min and the column temperature was set to 40 °C. DHPMA was determined using a QTrap 6500 (Sciex) after electrospray ionization in the negative mode with the ratio of the transitions *m/z* 236→107 (DHPMA) and *m/z* 238→107 ([d_5_]DHPMA). Details on the methods are described elsewhere (Abraham et al. 2021).

### High-resolution mass spectrometry (HRMS) of urinary metabolites

Metabolites of 3-MCPD were identified using an UltiMate 3000 liquid-chromatograph coupled to a QExactive Focus Orbitrap mass spectrometer (Thermo Fisher Scientific, Dreieich, Germany), using an HSS T3 column (2.1 × 100 mm, 1.8 µm, Waters) for chromatographic separation. Compounds were eluted with a gradient of water containing 0.5% formic acid (eluent A) and acetonitrile with 0.5% formic acid (eluent B). The flow rate of the gradient (0–3 min, 0% B; 3–10 min, 0–70% B; 10–12 min, 70% B; 12.01–15 min, 0% B) was 0.25 mL/min and the column temperature was 40 °C. Mass spectrometry was performed using electrospray-ionisation (ESI) in both positive and negative mode, using the following parameters: source temperature, 413 °C; capillary temperature, 256 °C; source voltage, 3.8 kV; S-lens radio frequency level, 60%. The nitrogen flow rates for sheath and auxiliary gas were 37 and 7 L/min, respectively. All data were acquired using a full scan mode covering the mass range from 50 to 550 m*/z* with a resolution of 70,000 and automatic gain control (AGC) setting of 3 × 10^6^ with a maximum injection time (IT) of 100 ms. For metabolite identification data-dependent MS^2^ (dd-MS^2^) was applied. The three most abundant precursor ions in each full scan were selected by the quadrupole, sent to the higher-energy collisional dissociation (HCD) cell for ion fragmentation and finally to the Orbitrap mass analyzer for detection. To increase coverage of the dd-MS^2^ acquisition for the identification of low abundance metabolites of 3-MCPD, an exclusion list was used containing ions obtained from an initial screening of pooled urine samples of untreated rats. The dd-MS^2^ scans were performed at a mass resolution of 17,500, intensity threshold of 1.6e5, isolation width of 1.0 m*/z* and normalized collision energy (NCE) of 50% with ± 20% step. Valid metabolites were identified comparing accurate masses, isotope distributions, retention times, and fragmentation patterns of ions in urine samples of 3-MCPD- and [^13^C_3_]3-MCPD-treated animals. The analyses of data-dependent acquisition results acquired in the ESI^+^ mode did not show any positively charged metabolites.

### NMR spectroscopy

NMR spectra were recorded on a 500 MHz NMR spectrometer system (VNMRS500, Varian Associates, Palo Alto, USA) operating at frequencies of 499.9 MHz for ^1^H and 125.71 MHz for ^13^C, which was equipped with a 5 mm OneNMR probe (Agilent Technologies, Santa Clara, USA). Incredible natural abundance double quantum transfer experiments (INADEQUATE) were done on a concentrated urine sample (5 mL) collected from three female rats that received a single dose of 50 mg/kg [^13^C_3_]3-MCPD (time interval 0–8 h). After dilution with 5 mL methanol, the mixture was vortexed, centrifuged and the supernatant was reduced to dryness under a stream of nitrogen. The residues were resuspended in 500 µL D_2_O, vortexed and centrifuged at 18.000 × g for 10 min. The INADEQUATE were carried out accumulating 256 scans with 128 steps in second dimension. Spectral width was set to 248.6 ppm with a transmitter offset of 110 ppm, using a 90° pulse of 9.68 µs with an acquisition time of 0.066 s and a relaxation delay of 1 s. Receiver gain was estimated using autogain function and set to the maximum value of 60 dB for ^13^C NMR experiments. For time efficiency a non-uniform-sampling (NUS) scheme with 50% sampling density was used for acquiring the INADEQUATE data, resulting in an overall measurement time of 589 min per experiment. During the experiment the sample was locked on ^2^H using a small amount of D_2_O, which was added to the sample for this purpose. The NMR probe temperature was kept at 15 °C during all measurement operations.

### Data analyses

Mass spectrometric data from targeted quantification analyses of DHPMA, β-ClLA, 3-MCPD sulfate, TDGA and [^13^C_2_]oxalic acid were processed using Analyst software version 1.7 (Sciex). GC–MS data of 3-MCPD in urine samples were analyzed using MassHunter Quantitative Analysis software version 10.1 (Agilent). The urinary excretion of 3-MCPD and its metabolites determined in three animals per group are reported as mean values ± standard deviations (SD). Differences were evaluated with the Student’s t-test using SigmaPlot 14.0 (Systat Software, Inc., Erkrath, Germany). Differences with *p*-values < 0.05 were considered statistically significant. Orbitrap data were evaluated using Freestyle™ software version 1.8 (Thermo Fisher Scientific). NMR data were processed using Maestre Nova NMR software version 15.0.1 (Mestrelab Research, Santiago de Compostela, Spain).

## Results

### 1D and 2D ^13^C NMR spectra of urinary [^13^C_3_]3-MCPD metabolites

**Fig. 1:**
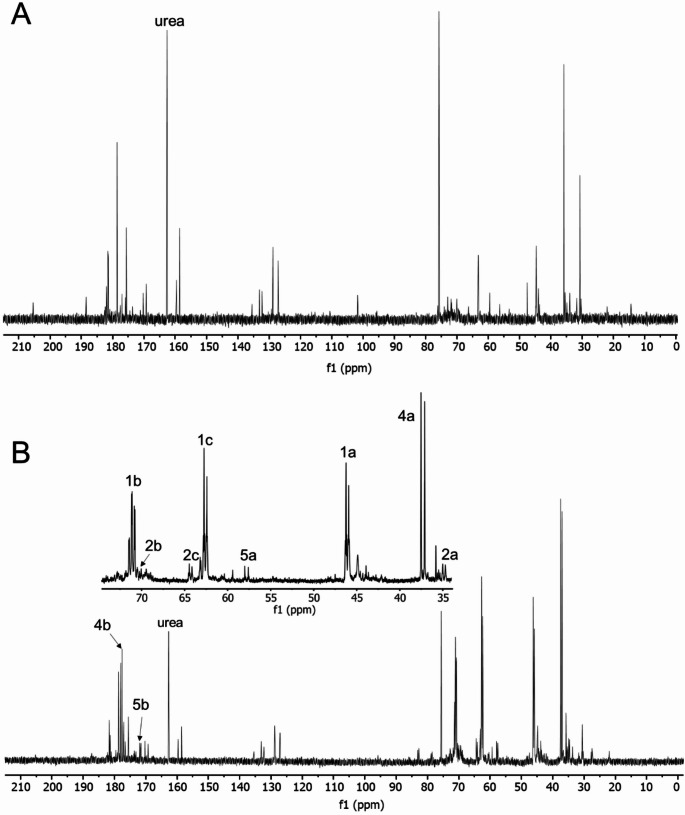
^1^H-decoupled ^13^C NMR spectra of concentrated pooled control rat urine (A) and concentrated pooled rat urine following administration of 50 mg/kg [^13^C_3_]3-MCPD (B), collected 8 h after dosing. Metabolite signals are labeled with a number and a letter according to the structural scheme of the parent molecule [^13^C_3_]3-MCPD (C_**a**_H_2_Cl-C_**b**_HOH-C_**c**_H_2_OH)

A ^13^C NMR spectrum of pooled control rat urine is depicted in Fig. 1A. The signals between d_C_ 170 and 190 ppm result mainly from ^13^C of carboxylic acid groups in, e.g., amino acids and citrate, and the intense single peak at about d_C_ 162.6 ppm was assigned to urea, a characteristic calibration signal for urine samples. Four signals around d_C_ 135 ppm originate from the ^13^C in the phenyl ring of hippurate. The signals between d_C_ 70 and 80 ppm result from ^13^C in glucose and other sugar molecules, and between d_C_ 20 and 65 ppm from ^13^C in methylene groups of, e.g., creatine, citrate, and different amino acids (Keun et al. 2002). These endogenous compounds appeared as single resonances in the ^13^C NMR spectrum due to the low probability of ^13^C-^13^C coupling (~ 0.012%). In contrast, the coupling of adjacent ^13^C atoms expected in the current study facilitated the detection of metabolites formed from [^13^C_3_]3-MCPD, leading to the frequent occurrence of multiplet patterns (Fig. 1B). The chemical shifts of signals related to metabolites of 3-MCPD are summarized in Table 1.

**Table 1:** Chemical shifts, multiplicities and connectivities of ^13^C resonances of urinary metabolites after [^13^C_3_]3-MCPD administration in rats

Compound no	Chemical shift^a^ (ppm)	Multiplicity	Connectivity from INADEQUATE	Chemical shifts calculated^b^ (ppm)	Chemical shifts reference substances^c^ (ppm)	Proposed carbon vicinity	Proposed molecule^d^
M2	34.86	d	2a	34.9	34.89	–CH_2_SR	DHPMA
M4	37.35	d	4a	34.8	33.90	–CH_2_SR	TDGA
M3	44.42	d	3a	47.6	47.14	–CH_2_Cl	3-MCPD sulfate
M1	46.10	d	1a	46.8	45.75	–CH_2_Cl	3-MCPD
M5	57.81	d	5a	61.6	57.40	–CH_2_SOR	TNDGA
M1	62.59	d	1c	63.8	62.46	–CH_2_OH	3-MCPD
M2	64.31	d	2c	65.3	64.28	–CH_2_OH	DHPMA
M3	69.42	d	3c	72.9	67.08	–COSO_3_	3-MCPD sulfate
M2	70.55	d,d	2b	70.8	70.61	–CHOH	DHPMA
M3	70.94	d,d	3b	73.2	68.96	–CHOH	3-MCPD sulfate
M1	71.02	d,d	1b	72.2	71.11	–CHOH	3-MCPD
M5	171.76	d	5b	175.2	173.03	–COOH	TNDGA
M4	177.74	d	4b	174.8	173.98	–COOH	TDGA

^a^Chemical shifts were from ^13^C (Fig. 1) or from INADEQUATE NMR spectra (Fig. 2)

^b^Chemical shifts of the suspected metabolites were predicted by nmrdb.org (https://www.nmrdb.org/13c/index.shtml?v=v2.138.0) (Banfi and Patiny 2008)

^c^Reference ^13^C NMR spectra were usually recorded in D_2_O

^d^acronyms (omitting the isotope-labeling): M1, 3-MCPD (3-monochloropropane-1,2-diol); M2, DHPMA (2,3-dihydroxypropyl mercapturic acid); M3, 3-MCPD sulfate (3-chloro-2-hydroxypropyl hydrogen sulfate); M4, TDGA (thiodiglycolic acid); M5, TNDGA (thionyldiglycolic acid)

**Fig. 2:**
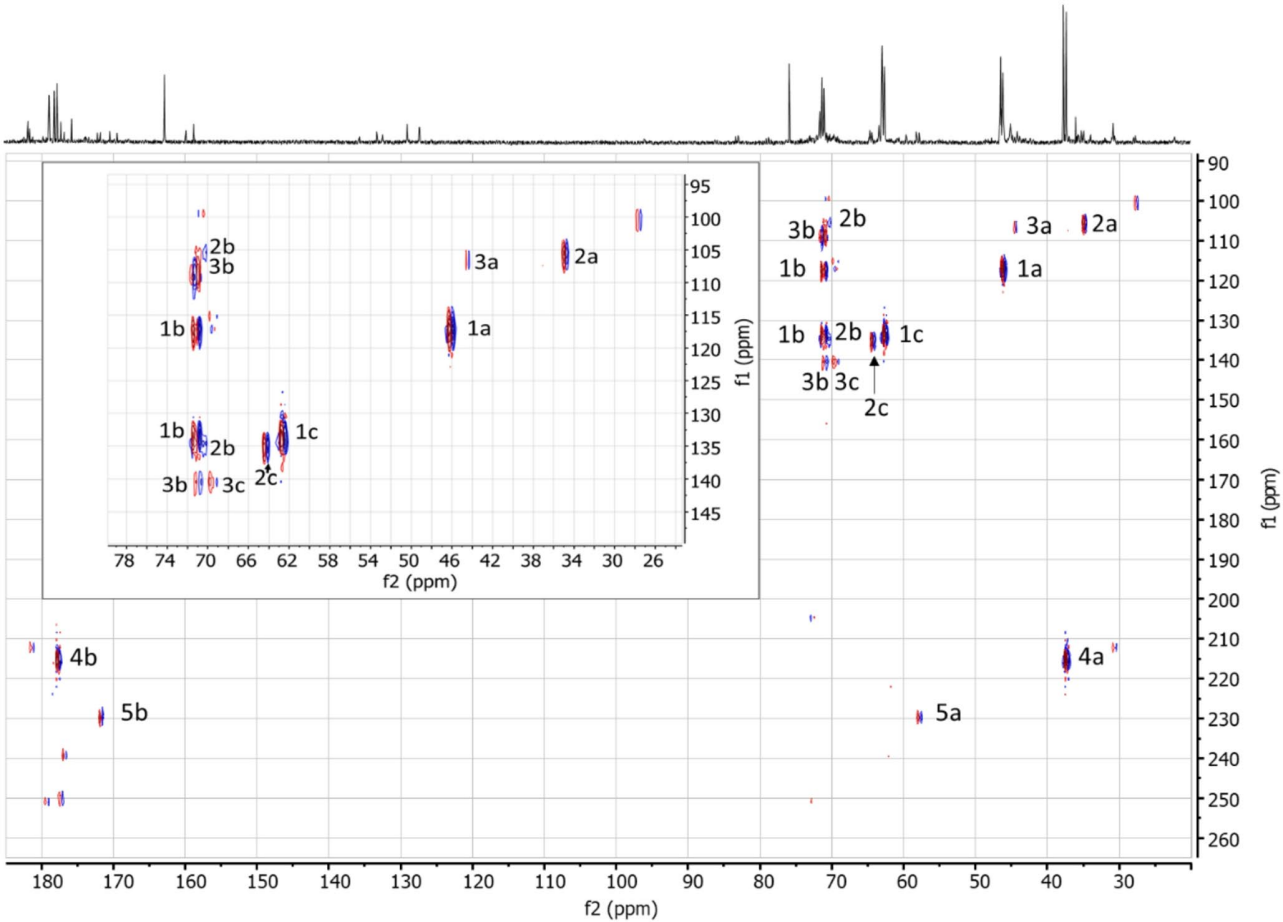
INDEQUATE spectrum from concentrated pooled rat urine collected 8 h after administration of 50 mg/kg bw [^13^C_3_]3-MCPD. The horizontal alignment (matching resonances on the f1 axis) of two signals with different resonances on the f2 axis indicates the vicinity of the two carbons. For example, the two contour signals 1b aligning vertically on the f1 axis each match the signals of 1a and 1c, which in turn align horizontally with 1b but not with any other resonance, indicating a metabolite with three adjacent carbon atoms (C_**a**_-C_**b**_-C_**c**_) derived from [^13^C_3_]3-MCPD (C_**a**_H_2_Cl-C_**b**_HOH-C_**c**_H_2_OH)

Coupling constants may be used to study connectivities of adjacent carbon atoms. The presence of multiple metabolites with structural similarities, however, impedes unambiguous assignments. Here, we used INADEQUATE to determine the connectivity between individual signals recorded by ^13^C NMR (Fig. 2). The occurrence of two signals with different chemical shifts (f2 axis) and the same double quantum frequency (i.e. aligned horizontally along the f1 axis) indicated that the respective ^13^C atoms were directly adjacent. The connectivities between ^13^C atoms are summarized in Table 1.

Tentative structural assignments of the metabolites were based on the comparisons of recorded and estimated chemical shifts, the signal multiplicities and the INADEQUATE connectivity data, which are summarized in Table 1. The structural hypotheses were confirmed later by ^13^C NMR spectra of synthesized reference materials. Five putative metabolites (M1–M5) were detected in the NMR spectra with varying confidence depending on the urinary concentrations. The INADEQUATE spectrum showed correlations between the three relatively strong resonances at d_C_ 46.10 (d, C1a), 62.59 (d, C1c), and 71.02 ppm (dd, C1b). The multiplicities indicated that C1a and C1c were endstanding with ^13^C chemical shifts agreeing to the assignments of -CH_2_Cl and -CH_2_OH, respectively, with C1b as the central carbon (-CHOH). In summary, metabolite 1 (M1) was assigned to the expected 3-MCPD. Two other systems including three resonances each were assigned to two metabolites (M2 and M3) observed in previous studies. The chemical shifts of the doublets at d_C_ 34.86 and 64.31 ppm (Fig. 1B) matched well with the expected structural elements of DHPMA (Abraham et al. 2021; Barocelli et al. 2011), the thioether carbon C2a (-CH_2_SR) and the endstanding C2c (–CH_2_OH), respectively. The expected multiplet signal (dd) of C2b was not detected in the ^13^C NMR spectrum, however, there was a weak correlation signal assigned to C2b observed in the INADEQUATE spectrum (Fig. 2).

Two other weak doublets observed by INADEQUATE were tentatively assigned to 3-MCPD sulfate (M3). The signals at d_C_ 44.42 ppm matched with the expected -CH_2_Cl (C3a) and at d_C_ 69.42 ppm with –CHOSO_3_^−^ (C3c). The central carbon (C3b, -CHOH) gave rise to two signals at d_C_ 70.94 ppm, which align horizontally with the signals of C3a and C3c (Fig. 2). The chemical shifts were consistent with those of the reference standard of 3-MCPD sulfate (3-chloro-2-hydroxypropyl hydrogen sulfate).

Two pairs of intense signals sharing the same double quantum frequency but without any other correlating signals were observed by INADEQUATE (Fig. 2), indicating the presence of metabolites with two remaining ^13^C. One pair of resonances (assigned to M4) were at d_C_ 37.35 ppm (d, C4a) and 177.74 ppm (d, C4b). The latter chemical shift (C4b) agreed with a carboxylic acid (-*C*OOH). The chemical shift of the other carbon (C4a) may result from a thioether group attached (-*C*H_2_SR). The other metabolite (M5) contained two carbons with the chemical shifts d_C_ 57.81 (d, C5a) and 171.76 (d, C5b). Again, the chemical shift of C5b indicated a carboxylic acid (-*C*OOH). The other signal was tentatively assigned to ^13^C in the vicinity of a thionyl group (-*C*H_2_SOR). It was hypothesized that these pairs of resonances resulted from the formation of thiodiglycolic acid (TDGA, M4) and the respective oxidation product thionyldiglycolic acid (TNDGA, M5), which required further confirmation.

The INADEQUATE spectrum did not allow a comprehensive assignment of all resonances observed. For example, a pair signals at d_C_ 37.35 ppm (d) and d_C_ 177.74 ppm (d) probably represented two adjacent ^13^C atoms of one metabolite, which, however, was not identified.

### Mass spectrometric identification of metabolites in urine samples of rats treated with 3-MCPD or [^13^C_3_]3-MCPD

**Fig. 3:**
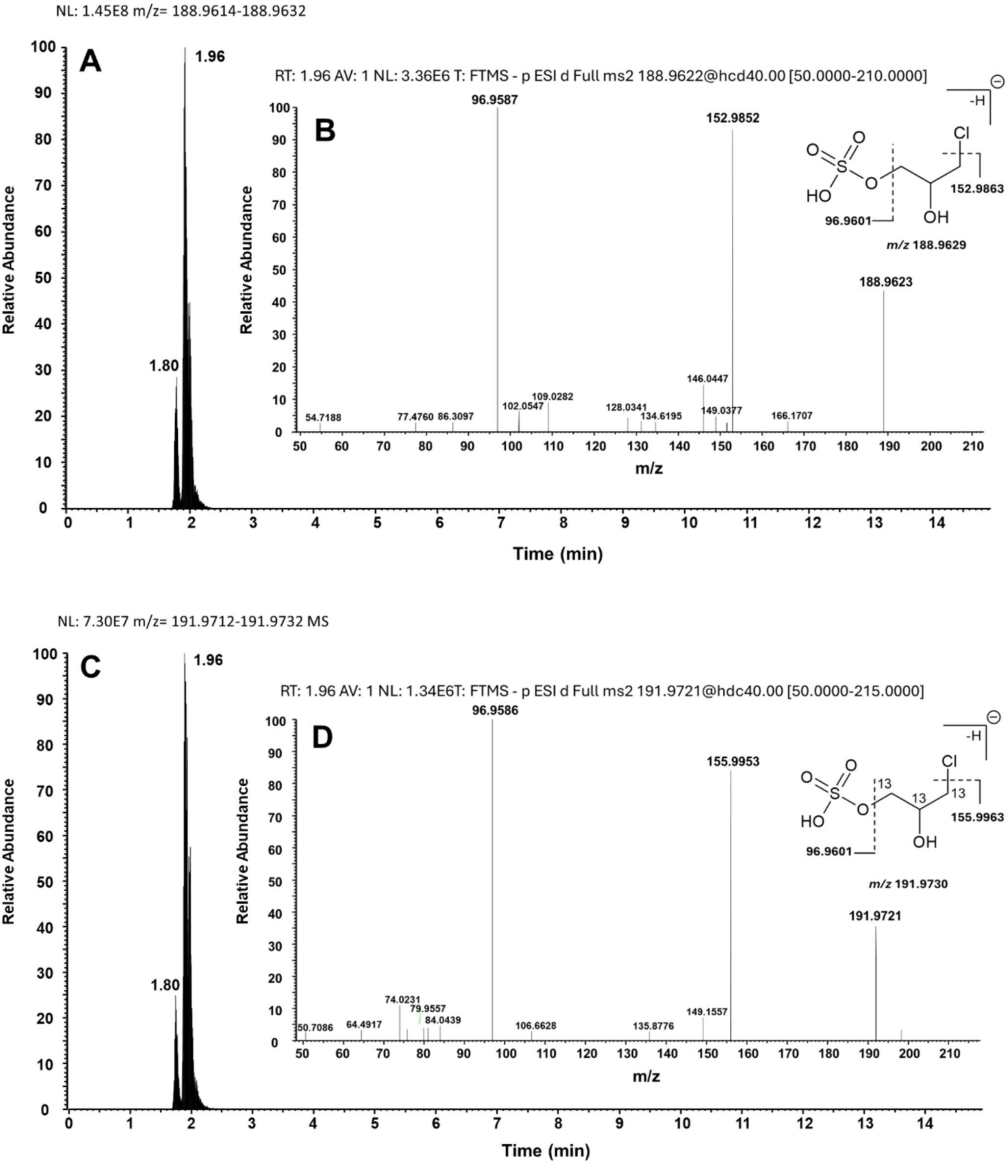
Extracted ion chromatograms of 3-MCPD sulfate in rat urine collected 8 h after treatment with 50 mg/kg 3-MCPD (**A**) and of [^13^C_3_]3-MCPD sulfate in rat urine collected 8 h after treatment with 50 mg/kg [^13^C_3_]3-MCPD (**C**). The insets show the respective fragment spectra (**B**, **D**)

Using data-dependent acquisition, a list of accurate masses of possible metabolites in urine samples from 3-MCPD- and [^13^C_3_]3-MCPD-treated animals was generated by comparison of retention times, isomer distributions, intensities and fragmentation spectra of unlabeled molecules and ^13^C-labeled isotopologues. An example, the data of the sulfo conjugate of 3-MCPD (two isomers), is shown in Fig. 3. Experimental fragment spectra and isotope distributions of the molecular peaks of all other 3-MCPD metabolites identified are summarized in the Supplemental Information (Figures S5–S18). Accurate masses, retention times, molecular formula and number of structural isomers are listed in Table 2.

**Table 2:** Accurate ion mass ratios and main fragments of 3-MCPD metabolites and their corresponding ^13^C isotopologues in rat urine samples after administration of 3-MCPD or [^13^C_3_]3-MCPD^a^

Compound no.^b^	RT^c^ [min]	Isomers	Formula	Measured *m/z* [M-H]^−^ [u]	Theoretical *m/z* [M-H]^−^ [u]	Fragments *m/z* [M-H]^−^ [u]	Compound
M2	4.32	1	C_8_H_15_NO_5_S	236.0599	236.0598	107.0158, 128.0340	DHPMA
	4.32	1	^13^C_3_C_5_H_15_NO_5_S	239.0690	239.0698	110.0255, 128.0339	[^13^C_3_]DHPMA
M3	1.80 1.96	2	C_3_H_7_ClO_5_S	188.9623	188.9629	152.9852, 96.9587	3-MCPD sulfate
	1.80 1.96	2	^13^C_3_H_7_ClO_5_S	191.9721	191.9730	155.9953, 96.9586	[^13^C_3_]3-MCPD sulfate
M4	4.20	1	C_4_H_6_O_4_S	148.9905	148.9914	105.0002	TDGA
	4.20	1	^13^C_2_C_2_H_6_O_4_S	150.9976	150.9981	106.0035, 107.0069	[^13^C_2_]TDGA
M5	1.30	1	C_4_H_6_O_5_S	164.9852	164.9863	n.d.^*c*^	TNDGA
	1.30	1	^13^C_2_C_2_H_6_O_5_S	166.9919	166.9930	n.d.^*c*^	[^13^C_2_]TNDGA
M6	2.43	1	C_3_H_5_ClO_3_	122.9840	122.9854	n.d.^*c*^	β-ClLA
	2.43	1	^13^C_3_H_5_ClO_3_	125.9939	125.9955	n.d.^*c*^	[^13^C_3_]β-ClLA
M7	4.16 4.39 4.62	3	C_9_H_15_ClO_8_	285.0382	285.0382	249.0615, 175.0240, 113.0231	3-MCPD glucuronide
	4.16 4.39 4.62	3	^13^C_3_C_6_H_15_ClO_8_	288.0484	288.0483	252.0717, 175.0237, 113.0229	[^13^C_3_]3-MCPD glucuronide
M8	4.15 4.56	2	C_8_H_13_NO_6_S	250.0392	250.0391	120.9951, 128.0340	CHPMA
	4.15 4.56	2	^13^C_3_C_5_H_13_NO_6_S	253.0493	253.0491	124.0053, 128.0340	[^13^C_3_]CHPMA
M9	3.26	1	C_5_H_8_O_5_S	179.0014	179.0019	135.0107, 90.9844, 87.0072	CMMLA
	3.26	1	^13^C_2_C_3_H_8_O_5_S	181.0080	181.0086	136.0141, 92.9911, 87.0072	[^13^C_2_]CMMLA

^a^HRMS extracted ion chromatograms, isotope patterns and MS^2^ spectra of all metabolites are summarized in the Supplemental Information (Figures S5–S18)

^b^3-MCPD (M1) was not detected by HRMS

^c^RT, retention time; n.d.; not detectable

Eight metabolites of 3-MCPD were characterized using HRMS, among them those detected by NMR spectroscopy (M2 to M5, Table 2). The formation of the expected mercapturic acid DHPMA (M2) was confirmed by detection of the anions with *m/z* 236.0599 (DHPMA) and with *m/z* 239.0690 ([^13^C_3_]DHPMA). Two analogous fragmentations of DHPMA (*m/z* 236.0599→107.0158/128.0340) were also observed for [^13^C_3_]DHPMA, which confirmed the structural assignment (Figures S5 and S6 in the Supplemental Information). 3-MCPD sulfate (M3, *m/z* 188.9623) showed a fingerprint product ion with *m/z* 96.9587 (hydrogensulfate anion), which resulted from a neutral loss of C_3_H_7_OCl (Fig. 3). The equivalent fragmentation was observed for [^13^C_3_]3-MCPD sulfate (*m/z* 191.9721→96.9586). A second fragmentation, a neutral loss of hydrogen chloride, led to the formation of a product ion with *m/z* 152.9852 from 3-MCPD sulfate and with *m/z* 155.9953 from the ^13^C_3_-labelled analogue. Two peaks in the chromatogram were an indication for the presence of two structural isomers. The retention time of the higher signal (1.96 min) was equivalent to that of the synthesized 3-chloro-2-hydroxypropyl hydrogen sulfate, which is depicted as 3-MCPD sulfate in Fig. 3. The minor peak may originate from sulfo conjugation at the hydroxy group at C2 of 3-MCPD. The putative metabolite TDGA (M4) appeared with a precursor ion mass of *m/z* 148.9902 and a dominant product ion with *m/z* 105.0001 resulting from a CO_2_ neutral loss of one of its two carboxylic acid groups (Figure S7). The same fragmentation was observed for [^13^C_2_]TDGA (*m/z* 150.9969), resulting in two product ions with *m/z* 106.0034 and 107.0068 following the neutral loss of either ^13^CO_2_ or CO_2_, respectively (Figure S8). The presence of the TDGA oxidation product TNDGA (M5) was confirmed by detection of the anion with *m/z* 164.9852 and its corresponding ^13^C-labeled anion with *m/z* 166.9919 (Figures S9 and S10). Due to the low intensity of the precursor ions, the fragment spectra for both substances were not recorded.

The metabolites described in the following (M6 to M9) were detected exclusively by HRMS. One of them, β-ClLA (M6), produced a rather faint signal in the current samples with *m/z* 122.9840 (Figure S11). The observation of the ^13^C-labeled anion (*m/z* 125.9939) confirmed the formation of β-ClLA (Figure S12). However, the concentrations of β-ClLA and [^13^C_3_]β-ClLA were too low for the recording of fragment spectra by dd-MS^2^. The precursor ion [M-H]^−^ with *m/z* 285.0382 was assigned to a glucuronic acid conjugate of 3-MCPD, in short 3-MCPD glucuronide (M7). According to fingerprint fragments specific for glucuronic acid conjugates (Bendadani et al. 2016; Staines et al. 2004), M7 abstracted two fragments with *m/z* 175.0240 (glucuronide anion) and 113.0231 (Figure S13). The same fragments were observed after collision-induced dissociation of [^13^C_3_]3-MCPD glucuronide (*m/z* 288.0484), observed in urine samples of [^13^C_3_]3-MCPD-treated rats (Figure S14). In addition, specific product ions resulting from a hydrogen chloride neutral loss from 3-MCPD glucuronide (*m/z* 285.0382→249.0615) and [^13^C_3_]3-MCPD glucuronide (*m/z* 288.0484→252.0717) were detected. The suggested molecular structures (Figures S13 and S14) are the most probable hypotheses. Considering that 3-MCPD can be glucuronidated at both hydroxyl groups, two regioisomers can be expected. Both may have four diastereomers because of the chiral centers at C2 of 3-MCPD and at C1 of the hexose ring (determining anomeric α- or β-configuration). The current data did not allow a more detailed analysis of the three peaks.

Another metabolite anion with *m/z* 250.0392 and a product ion with *m/z* 128.0340 (characteristic for mercapturic acids) was tentatively assigned 3-carboxy-2-hydroxypropyl mercapturic acid (CHPMA, M8, retention time 4.15 min; Figure S15). The assignment was confirmed by observation of the corresponding [^13^C_3_]CHPMA (*m/z* 253.0493) in urine samples of [^13^C_3_]3-MCPD treated rats, accompanied by the respective fragmentations *m/z* 253.0493 → 124.0053/128.0340 (Figure S16). The presence of two major peaks in the chromatograms of CHPMA and [^13^C_3_]CHPMA sharing the same masses of parent ions and fragments suggested the existence of a pair of diastereomers, differing by the chirality of the C2 in the 2-hydroxypropanoic acid group. One metabolite with a precursor ion [M-H]^−^ with *m/z* 179.0009 was assigned to 3-(S-carboxymethyl)mercaptolactic acid (CMMLA, M9, Figure S17). This fragmented into the anions of hydroxypropanoic acid (*m/z* 87.0072) and thioglycolic acid (*m/z* 90.9844). A corresponding pattern was observed for [^13^C_2_]CMMLA with [M-H]^−^ with *m/z* 181.0076 (Figure S18), displaying the same hydroxypropanoic acid product ion (*m/z* 87.0072) and the anion of [^13^C_2_]thioglycolic acid (*m/z* 92.9911).

### Quantification of urinary 3-MCPD metabolites

The urinary excretion of 3-MCPD and four of the metabolites detected by HRMS, i.e. DHPMA, β-ClLA, TDGA and 3-MCPD sulfate, were quantified by GC-, LC–MS/MS or UHPLC-MS/MS. The average excretion values in dependency of sex and dosage (5 or 50 mg 3-MCPD/kg bw) are summarized in Table 3. Unchanged 3-MCPD was excreted at relatively high levels in the high dose groups in comparison to the low dose groups in both males (7.3% vs 3.3%, *p* = 0.01) and females (9.7% vs 2.6%, *p* = 0.01). No significant sex-specific differences in both dose-groups were observed. The most abundant urinary metabolite was DHPMA. It was excreted at higher levels in males compared to females in both the high dose groups (3.5% vs 1.74%, *p* = 0.004) and low dose groups (4.6% vs 1.8% *p* = 0.001). The differences between high and low dose groups were not statistically significant. The excretion of β-ClLA was significantly higher in the high-dose group in both males (0.06%) and females (0.10%) when compared to the low dose groups of males (0.02%, *p* = 0.04) and females (0.04%, *p* = 0.005). There was no sex-specific difference of β-ClLA excretion. In contrast, TDGA excretion was higher in female than in male rats in both the high dose (4.5% vs 1.1%, *p* = 0.007) and low dose groups (5.0% vs 1.8%, *p* = 0.001), but no differences were observed between doses. The excretion of 3-MCPD sulfate was slightly higher in high-dosed females (0.22%) compared to low-dosed females (0.08%, *p* = 0.013), whereas no difference was observed in males. In the low-dose group, male rats (0.14%) excreted higher levels of 3-MCPD sulfate than females (0.08%, *p* = 0.048), while no sex difference was found in the high-dose group.

**Table 3:** Urinary excretion of 3-MCPD and four metabolites until 48 h after administration of two different doses of 3-MCPD. Values are means of three animals ± standard deviation (SD)

Compound	sex	% excreted after 50 mg/kg	% excreted after 5 mg/kg
3-MCPD	m	7.3 ± 1.2	3.3 ± 1.1
	f	9.7 ± 0.7	2.6 ± 1.1
DHPMA^a^	m	3.5 ± 0.4	4.6 ± 0.5
	f	1.7 ± 0.4	1.8 ± 0.3
β-ClLA	m	0.06 ± 0.03	0.02 ± 0.01
	f	0.10 ± 0.01	0.04 ± 0.02
TDGA^b^	m	1.1 ± 0.5	1.8 ± 0.2
	f	4.5 ± 1.0	5.0 ± 0.6
3-MCPD sulfate	m	0.21 ± 0.05	0.14 ± 0.03
	f	0.22 ± 0.04	0.08 ± 0.02
[^13^C_2_]oxalic acid^c^	m	0.04 ± 0.01	–
	f	0.03 ± 0.02	–

^a^Urine samples of the control rats contained DHPMA at low concentrations. The background corresponded to 0.5 to 3.7% and to 6.8 to 61.2% of the DHPMA concentrations in the high dose and the low dose groups, respectively, and were subtracted during data evaluation

^b^Background TDGA concentrations in untreated rats corresponded to between 1.7 and 34.4% and between 5.1 and 114% of the TDGA concentrations in the high dose and the low dose groups, respectively, and were subtracted during data evaluation

^c^Due to the natural background excretion of oxalic acid, we determined urinary concentrations of [^13^C_2_]oxalic acid in rats treated with 50 mg [^13^C_3_]3-MCPD/kg bw using an external calibration line of [^13^C_2_]oxalic acid prepared in pooled urine sample of untreated rats

Oxalic acid was suggested to be a major metabolite of 3-MCPD by Jones and Murcott (1976). In the current study, however, [^13^C_2_]oxalic acid was neither observed by ^13^C NMR spectroscopy nor by HRMS; the latter may be due to its dianionic nature with an *m/z* value below the detection range. Because oxalic acid was previously discussed as a major 3-MCPD metabolite with toxicological relevance, we determined [^13^C_2_]oxalic acid in urine samples of [^13^C_3_]3-MCPD-treated rats by ion-pair UHPLC-MS/MS. Mean amounts of 0.04% in male and 0.03% of the [^13^C_2_]3-MCPD doses in female rats were excreted as [^13^C_2_]oxalic acid within 48 h.

## Discussion

Most studies on mapping of metabolic pathways rely on either mass spectrometry (Jaster-Keller et al. 2023; Xiong et al. 2009) or NMR spectroscopy (Emwas et al. 2019; Fennell et al. 1991; Sumner et al. 1992). The combination of both techniques, however, brings about two major advantages. First, the confidence of structural characterization is higher compared to the application of one technique alone (Bendadani et al. 2016). Second, the combination approach permits an optimal coverage of metabolite detection. HRMS is a very sensitive technique, however, it is blind to uncharged molecules. The less sensitive NMR spectroscopy may detect compounds that go unnoticed by HRMS, like e.g. neutral or volatile molecules. The current study completed the scheme of 3-MCPD metabolism applying both HRMS and ^13^C NMR spectroscopy. The inclusion of ^13^C-labelled 3-MCPD yielded two benefits. First, the tentative assignments of metabolites by HRMS were confirmed by detection of the labelled congeners. Second, the presence of ^13^C in 3-MCPD metabolites enhanced the sensitivity of ^13^C NMR, which is normally limited by the natural scarcity of ^13^C (1.1% of all carbons).

**Fig. 4:**
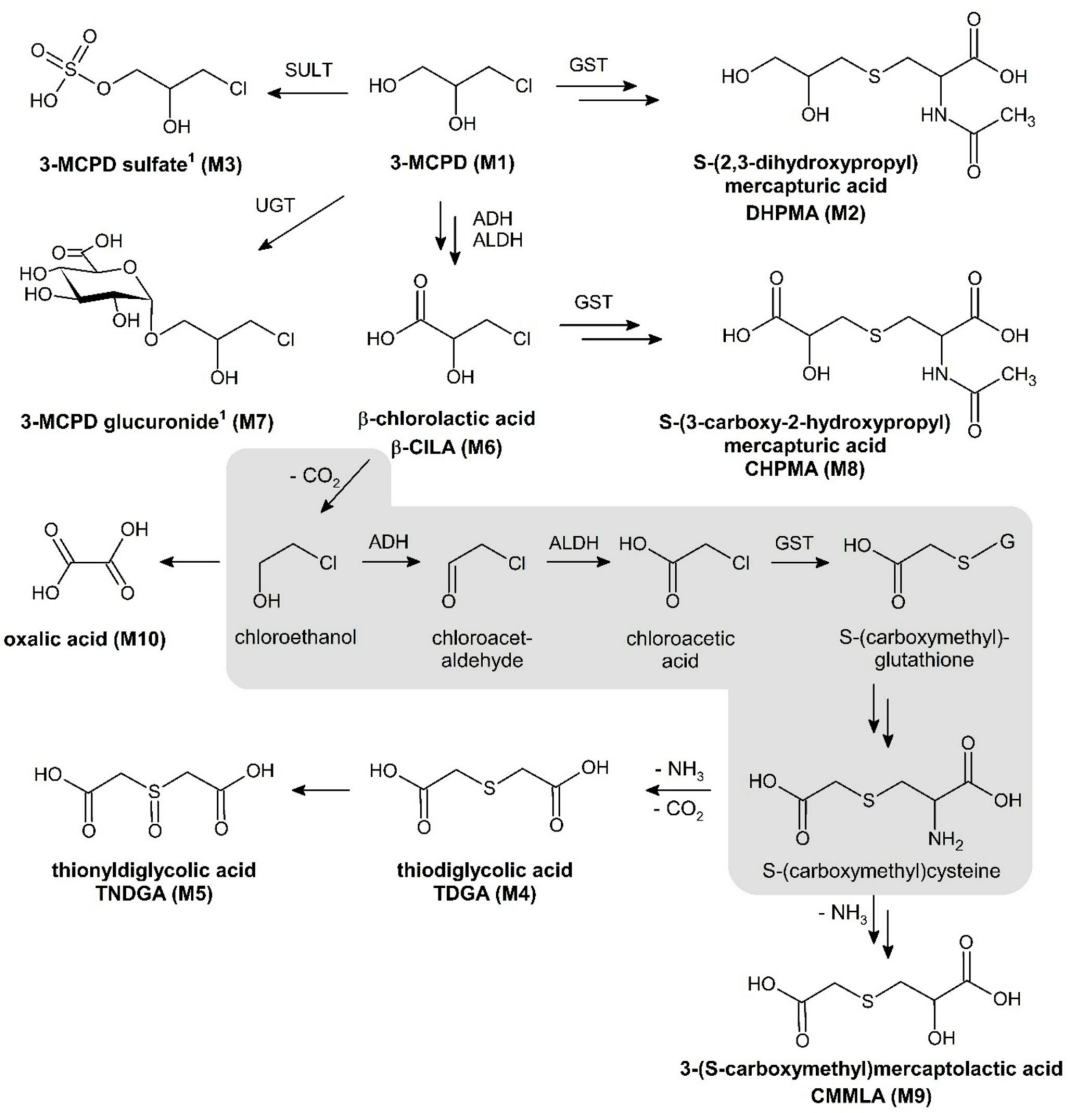
Extended scheme of 3-MCPD metabolism in rats. Metabolites detected in the current study are marked in bold letters. 3-MCPD sulfate and 3-MCPD glucuronide structures represent the most probable of two and three isomers, which were discernible by UHPLC. Pairs of arrows indicate the involvement of several transformations. The possible formation of 2-chloroethanol by decarboxylation of β-ClLA may explain the detection of CMMLA, TDGA and TNDGA in the current study. The metabolic intermediates shaded in grey were proposed (but also not detected) after direct 2-chloroethanol treatment of rats (Grunow et al. 1982). The enzyme classes involved were alcohol dehydrogenases (ADH), aldehyde dehydrogenases (ALDH), glutathione S-transferases (GST), sulfotransferases (SULT), and UDP-glucuronosyltransferases (UGT)

The overall scheme of urinary 3-MCPD metabolites with probable formation pathways is summarized in Fig. 4. Five of the ten compounds detected in the current study, i.e. 3-MCPD itself, DHPMA, β-ClLA, 3-MCPD sulfate and oxalic acid, have been discovered previously in urine samples of rats (Barocelli et al. 2011; Jia et al. 2022; Jones and Murcott 1976), and 3-MCPD, DHPMA, and β-ClLA also in human urine (Abraham et al. 2021; Bergau et al. 2021). The percentage excretions were largely in line with data from previous studies (Table 3). Barocelli et al. (2011) reported that 3.4 and 2.3% of an oral dose of 7.37 mg 3-MCPD/kg bw were excreted unchanged in male and female rats, respectively. A higher dose of 50 mg [^14^C]3-MCPD/kg bw administered intraperitoneally led to the excretion of 8.5% in male rats (Jones 1975). This matched the current observation that the 3-MCPD excretion was higher after administration of 50 mg/kg bw than after 5 mg/kg bw, which indicated a saturation of metabolism.

The most abundant metabolite in the current study was DHPMA, probably formed after glutathione conjugation of 3-MCPD (Jones 1975). The recoveries of urinary DHPMA were similar to those observed by Barocelli et al. (2011) and confirmed the observation that male rats excreted higher levels of [d_5_]DHPMA (6.2%) compared to female rats (1.2%) following a single administration of 29.5 (male) or 20.0 mg/kg bw (female) [d_5_]3-MCPD. It was reported before that excretion of metabolites formed after glutathione conjugation of, e.g. hexachlorobenzene (D'Amour and Charbonneau 1992), trichloroethylene (Lash et al. 1998), or benzaldehyde (Laham and Potvin 1987), were several fold higher in male rats than in female rats. This, however, has never been attributed to a single sexual dimorphism, because of the variety of enzymes involved in mercapturic acid formation, e.g. glutathione S-transferases, $\gamma$-glutamyltransferases, dipeptidases, and N-acetyltransferases (Hanna and Anders 2019). Following an oral dose of 29.5 mg 3-MCPD/kg bw, Barocelli et al. (2011) observed that approximately 0.9% (males) and 1.7% (females) of the dose was excreted as β-ClLA, which is an order of magnitude higher compared to the current data. β-ClLA is probably formed by oxidation of 3-MCPD via β-chlorolactaldehyde, a suspected reactive intermediate of 3-MCPD metabolism (Jones et al. 1978). This compound, however, or any other aldehyde, was not detected by NMR spectroscopy in the current study. β-ClLA may be detoxified by glutathione conjugation, and the corresponding 3-carboxy-2-hydroxypropyl mercapturic acid (CHPMA) was observed by HRMS in the urine samples (Fig. 4).

In contrast to the current data and to those of Barocelli et al. (2011), Jones et al. (1978) reported that at least 23% of a dose of 3-MCPD administered in rats was excreted as β-ClLA. The authors further suggested that oxalic acid was the main metabolite formed from β-ClLA (Jones and Murcott 1976), and remarked that it was not possible to quantify the urinary oxalic acid due to the precipitation of calcium oxalate crystals in the kidney. These, however, were merely identified microscopically as characteristic envelope-shaped crystals (Jones et al. 1978). In addition, the renal deposits of calcium oxalate crystals were only observed following i.p. administration of 120–150 mg/kg β-ClLA. In the current study, excretion levels of β-ClLA and oxalic acid were very low (< 0.1% of the dose). A decrease of [^13^C_2_]oxalic acid excretion over time was observed, being readily detectable in the first (0–8 h) and hardly quantifiable in the last urine samples (24–48 h, data not shown). This contradicted the notion that a reservoir of oxalate crystals in kidney tubuli leads to a continuous urinary excretion of oxalic acid. Our findings, consistent with the low β-ClLA excretion observed by Barocelli et al. (2011), indicated that oxidative metabolism of 3-MCPD is probably less relevant than believed previously, or that β-ClLA is detoxified efficiently by alternative metabolic pathway(s).

The two 3-MCPD conjugates resulting from sulfo conjugation and glucuronidation have been suspected by Barocelli et al. (2011) however, could not be detected by mass spectrometry in rat urine samples after treatment with [d_5_]3-MCPD (29.5 mg/kg for males, 20 mg/kg for females) or with [d_5_]3-MCPD palmitic diester (156.7 mg/kg). In a later study, two isomers of 3-MCPD sulfate were detected by HRMS in rat urine after a single oral administration of 50 mg/kg bw 3-MCPD (Jia et al. 2022). The current data confirmed the presence of two isomers of 3-MCPD sulfate. The structure of isomer 1 with the retention time of 1.96 min (Fig. 3), presumably 3-chloro-2-hydroxypropyl hydrogen sulfate (as depicted in Fig. 4), was confirmed by chemical synthesis. The minor peak was probably 1-chloro-3-hydroxypropan-2-yl hydrogen sulfate (isomer 2) resulting from sulfo conjugation of the hydroxy group at C2 of 3-MCPD. Assuming similar mass spectrometric fragmentation properties for the 3-MCPD sulfate esters, we used both peaks for quantification with an external calibration line of chemically synthesized 3-chloro-2-hydroxypropyl hydrogen sulfate (isomer 1), showing that sulfo conjugation contributed to less than < 1% to the overall metabolism of 3-MCPD (Table 3). The identification of (at least three structural isomers of) 3-MCPD glucuronide by UHPLC (Figures S12 and S13), exact mass and the fingerprint fragments was very specific (Table 2). One of the most probable isomers is depicted representatively in Fig. 4. The current HRMS and NMR spectroscopy data did neither allow structural assignments of the 3-MCPD glucuronide isomers nor conclusions on the urinary concentrations.

The INADEQUATE spectra indicated the formation of carboxylic acids, which apparently retained only two carbons of the parent 3-MCPD (Fig. 2). HRMS data and recording ^13^C NMR spectra of the commercially available compound confirmed the presence of TDGA. The TDGA in urine samples of [^13^C_3_]3-MCPD-treated rats (Figure S8) contained two ^13^C. The other half of the asymmetric thioether contained the naturally abundant ^12^C, probably originating from glutathione conjugation. This matches with a possible formation pathway for TDGA that was described previously for a variety of other chloroalkanes in rats or mice, e.g. vinyl chloride (Plugge and Safe 1977), 1,1-dichloroethylene (Jones and Hathway 1978), 1,2-dichloroethane (Yllner 1971), 2,2′-bis(chloroethyl)ether (Muller et al. 1979), and 2-chloroethanol (Grunow and Altmann 1982), all of which were metabolized via formation of the reactive intermediates 2-chloroacetaldehyde and 2-chloroacetic acid. For example, Grunow et al. (1982) described that after a single application of 5 mg/kg bw [^14^C]2-chloroethanol by gavage in rats, 45% of the dose was excreted as [^14^C]TDGA. The parent molecule but also the expected intermediate products [^14^C]2-chloroacetaldehyde and [^14^C]2-chloroacetic acid were not detected in the urine, indicating efficient oxidation and glutathione conjugation reactions. Also, expected turnover intermediate products of glutathione conjugation, especially S-carboxymethylcysteine, were not found, which was explained by a rapid progression of its conversion by deamination and decarboxylation (Grunow and Altmann 1982). These reports led to the pathway hypothesis outlined in Fig. 4. We assumed that β-ClLA was decarboxylated to 2-chloroethanol. Due to the fast processing, the following intermediate metabolites were not detected. Except TDGA, two other metabolites were observed that fit to this assumed pathway. The sulfoxide TNDGA has been described frequently as a co-metabolite formed by oxidation of TDGA, e.g. after treating rats with 2-chloroethanol (Grunow and Altmann 1982) or with acrylonitrile (Fennell et al. 1991). In addition, the HRMS data indicated the formation of CMMLA, which has been observed previously being formed after an oral dose of S-carboxymethylcysteine and excreted in human urine (Hofmann et al. 1991), possibly by transamination of S-carboxymethylcysteine and subsequent reduction of the ketone group. In 3-MCPD treated rats, the detection of CMMLA confirmed the possible metabolism after glutathione conjugation of 2-chloroacetic acid and formation of S-carboxymethylcysteine (Fig. 4).

Chronic toxicity of 3-MCPD manifested itself in form of renal tubular hyperplasia and other nephrotoxic effects as well as Leydig cell tumors in two-year bioassays with rats (Cho et al. 2008; Sunahara et al. 1993). However, the respective molecular mechanisms were poorly characterized (Eisenreich et al. 2023). It was hypothesized that a reactive (yet undetected) metabolite resulting from 3-MCPD oxidation, 3-chlorolactaldehyde, interferes with glycolysis by inhibiting glyceraldehyde-3-phosphate dehydrogenase (GAPDH) and triosephosphate isomerase (Jones and Porter 1995). Transcriptome analyses in kidneys, liver and testes after oral exposure of 3-MCPD or its dipalmitate ester in rats over 28 days showed the deregulation of multiple enzymes involved in glycolysis (phosphopyruvate hydratase), gluconeogenesis (pyruvate carboxylase), and in oxidative stress response (Buhrke et al. 2017). One question of the current work was if detection of yet unknown metabolites may lead to novel hypotheses for modes of action of 3-MCPD mediated toxicity. The experimental set up of urinary metabolite detection was not a perfect measure to do so, because toxic/reactive metabolites are usually detoxified and excreted as comparably inert molecules. Such a compound is the newly identified 3-MCPD metabolite TDGA. It is formed by many toxicologically relevant chloroalkanes (e.g. vinyl chloride (Plugge and Safe 1977)) via several intermediates, i.e. 2-chloroethanol, 2-chloroacetaldehyde and 2-chloroacetic acid. These compounds did not show specific nephrotoxic effects, however, the studies lacked histological examinations of the kidney (Bryant et al. 1992; Hartwig 2022). Comparably well studied is the release of the intermediate 2-chloroacetaldehyde in the kidney after ifosfamide therapy, which is associated with nephrotoxic effects observed in humans (Schwerdt et al. 2006; Springate 1997) and in rats (Han et al. 2021). 2-Chloroacetaldehyde formed from ifosfamide was detoxified by glutathione conjugation leading to the formation and excretion of S-carboxymethylcysteine and TDGA (Beyoğlu et al. 2025). Possible toxic mechanisms were studied by incubating rat renal cortical slices metabolizing physiological concentration of lactate with 2-chloroacetaldehyde. First, depletion of renal glutathione was associated with increased oxidative stress. Second, cellular ATP levels were reduced, which was attributed to the inhibition of oxidative phosphorylation in complex I of the mitochondrial respiratory chain by Knouzy et al. (2010). And third, pyruvate accumulation reflected the interference with energy metabolism. This was explained by either inhibition of the citrate cycle through pyruvate carboxylase and pyruvate dehydrogenase, or by inhibition of gluconeogenic/glycolytic enzymes, mainly glyceraldehyde 3-phosphate dehydrogenase (Knouzy et al. 2010). The induction of oxidative stress and the effect on cellular energy metabolism indicated similar mechanisms of nephrotoxicity of 3-MCPD and ifosfamide. These insights from mechanistic in vitro studies with ifosfamide/2-chloroacetaldehyde (Knouzy et al. 2010) are difficult to compare with newer indications on 3-MCPD modes of action deduced primarily from transcriptome analyses, indicating deregulation of glycolysis, gluconeogenesis, and oxidative stress responses in kidneys, liver and testes of rats (Buhrke et al. 2017). Taken together, 2-chloroacetaldehyde may be a reactive intermediate not only from ifosfamide but also of 3-MCPD, however, the current knowledge on common molecular mechanisms of nephrotoxicity is not sufficient to confirm this.

Another goal of the present study was to identify a biomarker candidate with a favorable sensitivity and specificity profile for exposure monitoring of 3-MCPD and its fatty acid esters in humans. As yet, the daily excretion of 3-MCPD in 24 h urine samples was used (Abraham et al. 2021). The specificity allowed the application for the estimation of the dietary intake from the daily excretion (Monien et al. 2025). The disadvantages are the low excretion level (3-MCPD makes out only 3.7% of the overall dose in a study with 12 participants) (Abraham et al. 2021), and the high effort of sample preparation previous to GC–MS analysis. In the current study on rat urine, only TDGA was excreted in amounts comparable to 3-MCPD. However, this metabolite is also formed from various other substances (Fennell et al. 1991; Grunow and Altmann 1982; Jones and Hathway 1978; Muller et al. 1979; Plugge and Safe 1977; Yllner 1971), suggesting a lack of specificity. Only the glucuronide and sulfo conjugates are probably very specific for 3-MCPD exposure. However, 3-MCPD sulfate is formed in small amounts only, and, based on low HRMS signal intensities, the same is assumed for the glucuronide isomers. To finally evaluate the biomarker potential, all metabolites will be tested in 24 h urine samples of humans (n = 12) from the controlled exposure study with hazelnut oil mentioned previously (Abraham et al. 2021).

A limitation of the current work was the focus on urinary excretion. Previous studies on other heat-induced food contaminants showed that high amounts of the doses were recovered in urine samples over 24 h, e.g. ~ 50% of acrylamide (Sumner et al. 1992), and ~ 55% of acrylonitrile (Fennell et al. 1991). In the current study, only 9.5% (females) to 9.9% (males) of the original 3-MCPD dose in the low-dose groups (5 mg/kg bw) and 12.2% (males) to 16.3% (females) in the high-dose groups (50 mg/kg bw) were recovered in urine after 48 h. The absence of other prominent signals in ^13^C NMR spectra and HRMS chromatograms supported the notion that most of the 3-MCPD metabolites may be excreted via other routes (e.g. via feces or exhalation). A previous study showed that merely 1–3% of the radioactive dose was recovered in the feces in animal studies with rats, rabbits and monkeys treated with [^14^C]3-MCPD (Kirton et al. 1969). In contrast, exhalation was reported to play a major role. About 30% of an i.p. dose of 100 mg/kg [^14^C]3-MCPD in rats was recovered as [^14^C]CO_2_ within 24 h (Jones 1975). It is also possible that carbons of 3-MCPD remain in the rat longer than 48 h, e.g. after reaction of reactive metabolites with nucleophiles (proteins or DNA) or by incorporation of degradation products into physiological pathways (like amino acid or fatty acid synthesis). For example, whole body autoradiography of rats dosed with [^14^C]3-MCPD after 24 h and 4 d showed accumulations of radioactivity in the cauda epididymis as well as liver and kidney. Possible mechanisms discussed included direct alkylation by 3-MCPD (or reactive metabolites) or incorporation into phospholipid membranes (Crabo and Appelgren 1972). Radioactivity was also detected in lipid extracts of various rat tissues after treatment with either [^36^Cl]3-MCPD or [^14^C]3-MCPD, showing that 3-MCPD was present unaltered but also after dechlorination and subsequent incorporation into triglycerides (Edwards et al. 1975). Unfortunately, both studies by Crabo (1972) and Edwards (1975) lack calculations of dose ratios in specific tissues. A study on a quantitative monitoring of distribution and excretion of radioactivity after treatment with [^14^C]3-MCPD is not available.

## Conclusion

The previous quantitative data on 3-MCPD metabolism in rats was largely confirmed, except that the present results contradicted indications of Jones et al*.* supporting that β-ClLA and oxalic acid may be major metabolites in rats (Jones et al. 1978; Jones and Murcott 1976). Five unknown metabolites were detected, namely TDGA, TNDGA, 3-MCPD glucuronide, CHPMA, and CMMLA (Fig. 4). Most of these metabolites reflect yet unknown pathways of detoxification, while TDGA indicated the interim formation of the reactive intermediate 2-chloroacetaldehyde. This is also released as a toxic agent from the cytostatic drug ifosfamide in the kidneys, where it leads to similar nephrotoxic effects as those observed after 3-MCPD administration in rats. Compared to the other 3-MCPD metabolites, 2-chloroacetaldehyde is a reactive intermediate, which forms etheno adducts in DNA (e.g. 1,*N*6-ethenodeoxyadenosine, εdA) (Oesch and Doerjer 1982; Pandya and Moriya 1996), and was mutagenic in different in vitro assays, e.g., in *S. typhimurium* TA1535 (Rannug et al. 1976) and in *E. coli* strains (Perrard 1985). The possible formation of 2-chloroacetaldehyde from 3-MCPD must be confirmed.

## Supplementary Information

Below is the link to the electronic supplementary material.

Supplementary Material 1

## Abbreviations

3-MCPD: 3-Monochloropropane-1,2-diol
β-ClLA: -β-chlorolactic acid
BMDL: Benchmark dose level
bw: Body weight
CHPMA: 3-Carboxy-2-hydroxypropyl mercapturic acid
CMMLA: 3-(S-carboxymethyl)mercaptolactic acid
DHPMA: 2,3-Dihydroxypropyl mercapturic acid
EFSA: European Food Safety Authority
GC-MS: Gas chromatography-mass spectrometry
HRMS: High-resolution mass spectrometry
INADEQUATE: Incredible natural abundance double quantum transfer experiment
IPC: Ion-pair liquid chromatography
MRM: Multiple reaction monitoring
NMR: Nuclear magnetic resonance
TDGA: Thiodiglycolic acid
TNDGA: Thionyldiglycolic acid
TDI: Tolerable daily intake
UHPLC-MS/MS: Ultra-high performance liquid chromatography-tandem mass spectrometry
