# Supplementary material for: Characterization and quantitation of urinary metabolites of 3-monochloropropane-1,2-diol (3-MCPD) in rats
Source: Arch Toxicol. 2026 Mar 10;100(6):2433–49. doi: 10.1007/s00204-026-04318-x (PMC13221421; doi:10.1007/s00204-026-04318-x)
Supplement: Supplementary file 1 — Supplementary Material 1 [file 204_2026_4318_MOESM1_ESM.docx]

**Characterization and Quantitation of Urinary Metabolites of 3-Monochloropropane-1,2-diol (3-MCPD) in Rats**

Thorsten Henning, Cornelius Goerdeler, Ahmed H. El-Khatib, Klas Meyer, Gustav G. Bruer, Klaus Abraham, Bernhard H. Monien

**Supplemental Information**

**Simultaneous quantification of thiodiglycolic acid (TDGA) and 3-MCPD sulfate in rat urine samples by ion-pair chromatography MS/MS**

TDGA and 3-MCPD sulfate concentrations in urine samples were determined according to a protocol for the analysis of organic acids based on ion-pair chromatography (IPC) developed by Bergau et al. {Bergau, 2021 #1549}, and adapted to the quantification of 3-MCPD metabolites. Aliquots of 20 µL urine were mixed with 10 µL [d_4_]TDGA solution (100 µg/mL in water), diluted with 370 µL eluent A and vortexed for 1 min. Isotope-labeled 3-MCPD sulfate was not available, and, thus, the quantification of 3-MCPD sulfate was based on an external calibration line of chemically prepared 3-chloro-2-hydroxypropyl hydrogen sulfate. It was prepared from 11 solutions of 3-MCPD sulfate (containing 0.01, 0.025, 0.05, 0.1, 0.25, 0.5, 1.0, 2.5, 5.0, 10 and 25 µM as well as 10 mM tributylamine and 0.25% acetic acid) prepared in the presence of 20 µL pooled rat urine of six control animals (devoid of 3-MCPD sulfate). The calibration mixtures were treated as normal samples. After preparation, samples were centrifuged at 18,000×g for 5 min (4°C). Aliquots of 200 µL were transferred into glass vials with micro inserts and 8 µL were injected into an LC system consisting of an HPLC 1100 (Agilent) equipped with a Nucleoshell RP 18 plus (2.0×150 mm, 2.7 μm; Macherey–Nagel) connected to a triple quadrupole-hybrid ion trap mass spectrometer QTrap 6500 (Sciex). The eluents were water containing 10 mM tributylamine and 0.25% acetic acid (A) and acetonitrile (B). The flow rate of the gradient (0-3 min, 2% B; 3-4 min, 2-25% B; 4-6 min, 25% B; 6-8 min, 25-95% B; 8-10 min, 95% B; 10-10.1 min, 2% B; 10.1-15 min, 2% B) was 0.5 mL/min. The temperature of the column oven was set to 40°C. The operating parameters of the QTrap 6500 were: ion spray voltage 4500 V, interface heater temperature 450°C, curtain gas 40 psi, ion source gas 1 60 psi, ion source gas 2 50 psi, collision activated dissociation gas set to medium. The multiple reaction monitoring (MRM) mode was employed for quantitative analysis. Declustering potentials and other fragmentation specific values for the detection of TDGA and 3-MCPD sulfate are summarized in Table S1. Data acquisition and processing were carried out using Analyst 1.7.1 software (Sciex).

Representative chromatograms of 3-MCPD sulfate and TDGA in a rat urine sample collected within 8 h after administration of 3-MCPD (50 mg/kg bw) are shown in Figures S1 and S2, respectively. It is of note that both isomers of 3-MCPD sulfate (3-chloro-2-hydroxypropyl hydrogen sulfate and 1-chloro-3-hydroxypropan-2-yl hydrogen sulfate) present in rat urine samples were considered for quantification. The external calibration of 3-MCPD sulfate prepared by custom synthesis (exclusively 3-chloro-2-hydroxypropyl hydrogen sulfate) is shown in Figure S3, together with the corresponding data for [d_4_]TDGA. The range of linear detection and the limit of quantification (LOQ) for 3-MCPD sulfate were 0.01 – 25 µM and 0.01 µM, respectively, and 0.05 – 25 µM and 0.1 µM for [d_4_]TDGA, respectively.

**Quantification of [^13^C_2_]oxalic acid in rat urine samples by ion-pair chromatography MS/MS**

The analysis of [^13^C_2_]oxalic acid in rat urine samples required transferring of the method for the analysis of TDGA and 3-MCPD sulfate onto a UPLC-MS/MS. Due to the high urinary background of natural oxalic acid, the turnover of 3-MCPD to oxalic acid was estimated from the animals treated with [^13^C_3_]3-MCPD. For the quantification of [^13^C_2_]oxalic acid, an external calibration line was applied, consisting of six dilutions of [^13^C_2_]oxalic acid in pooled urine of six control rats (2.72, 5.43, 13.6, 27.2, 54.3, 136 µM), which were processed as normal samples (Figure S4). Aliquots of 200 µL urine were mixed with 40 µL 2 M aqueous HCl. After stirring for 16 h (37°C), sample aliquots (20 µL) were neutralized with 8 µL 1 M aqueous ammonia, diluted with 72 µL ultrapure water and further with 400 µL 10 mM tributylamine and 10 mM acetic acid (eluent A). The samples were centrifuged at 18,000×g for 15 min (4°C), and aliquots of 5 µL of the supernatant were injected. [^13^C_2_]Oxalic acid was separated using an Acquity I-Class UPLC (Waters, Eschborn, Germany) equipped with an BEH C18 column (2.1×100 mm, 1.8 µm, Waters) and connected to a QTrap 6500 (Sciex). The eluents were water containing 10 mM tributylamine and 10 mM acetic acid (A) and acetonitrile (B).

The flow rate of the gradient (0–1 min, 3% B; 1–4 min, 3–90% B; 4–5 min, 90% B; 5–5.01 min, 90–3% B; 5.01–8 min, 3% B) was 0.35 mL/min. The temperature of the column oven was set to 35°C. [^13^C_2_]Oxalic acid was determined after electrospray ionization in the negative mode with the transition *m/z* 91.0 → 62.0. The operating parameters of the QTrap 6500 were: ion spray voltage 4500 V, interface heater temperature 450°C, curtain gas 40 psi, ion source gas 1 60 psi, ion source gas 2 50 psi, collision activated dissociation gas set to medium. Declustering potentials and other fragmentation specific values for the detection of [^13^C_2_]oxalic acid are summarized in Table S1. Data acquisition and processing were carried out using Analyst 1.7.1 software (Sciex). A representative chromatogram of [^13^C_2_]oxalic acid in a rat urine sample collected within 8 h after administration of [^13^C_3_]3-MCPD (50 mg/kg bw) is shown in Figure S3. The range of linear detection and the approximated LOQ for [^13^C_2_]oxalic acid were 2.72 - 136 µM (Figure S4, lower panel) and 2.72 µM (S/N = 8.4), respectively.

**Table S1**. Mass spectrometric parameters used for detection of TDGA, 3-MCPD sulfate and [^13^C_2_]oxalic acid (bold letters mark the transitions used for quantification).

| **analyte** | **Q1 *m/z*** | **Q3 *m/z*** | **CE [eV]** | **DP [V]** | **CXP [V]** | **EP [V]** |
| --- | --- | --- | --- | --- | --- | --- |
| **TDGA** | **149.0** | **104.8** | **-12** | **-20** | **-10** | **-10** |
|  | 149.0 | 41.0 | -60 | -20 | -10 | -10 |
| **[d_4_]TDGA** | 152.9 | 108.8 | -12 | -20 | -10 | -10 |
|  | 152.9 | 42.1 | -60 | -20 | -10 | -10 |
| **3-MCPD sulfate** | **188.9** | **152.7** | **-17** | **-20** | **-10** | **-10** |
|  | 188.9 | 96.8 | -38 | -20 | -10 | -10 |
| **[^13^C_2_]oxalic acid** | **91.0** | **62.0** | **-12** | **-20** | **-10** | **-10** |
| CE, collision energy; DP, declustering potential; CXP, cell exit potential; EP, entrance potential | | | | | | |

| 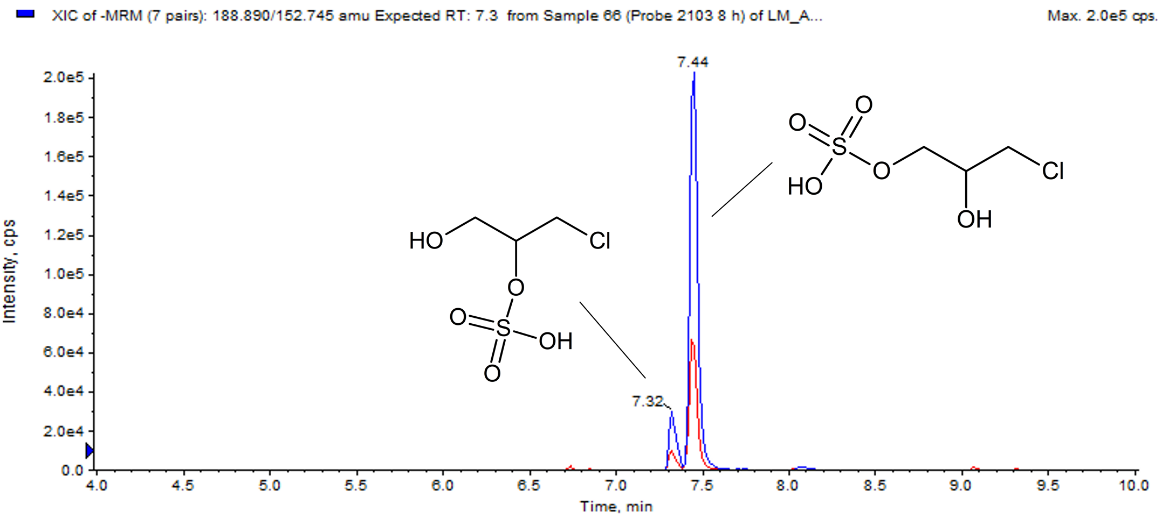 |
| --- |
| **Figure S1**. Extracted ion chromatogram of two isomers of 3-MCPD sulfate (*m/z* 188.9 → 152.7, blue, and *m/z* 188.9 → 96.8, red) detected in a urine sample 8 h after administration of 3-MCPD (50 mg/kg bw). |

| 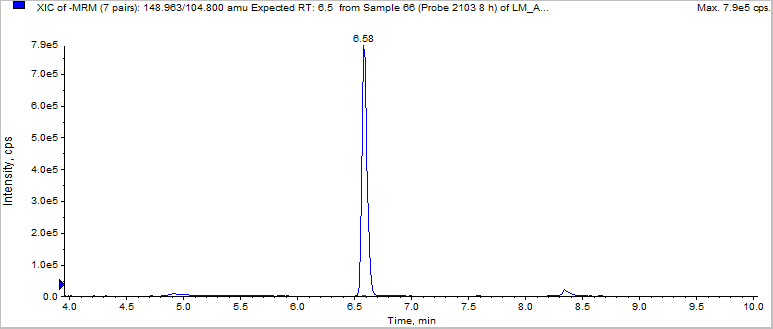 |
| --- |
| 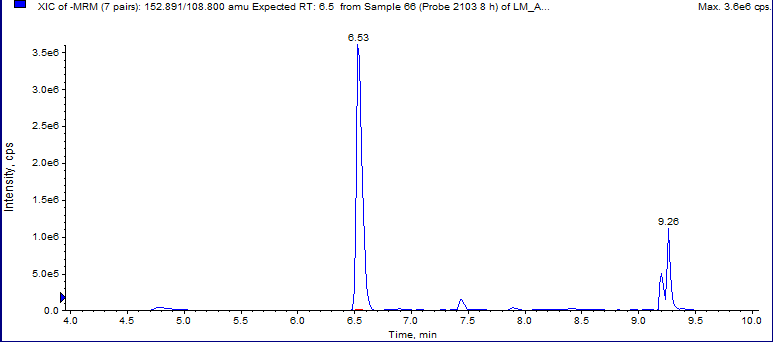 |
| **Figure S2**. Extracted ion chromatograms of TDGA (*m/z* 149.0 → 104.8; upper panel) detected in a urine sample 8 h after administration of 50 mg 3-MCPD/kg bw, and the deuterated standard [d_4_]TDGA (*m/z* 152.9 → 108.8; lower panel). |

| 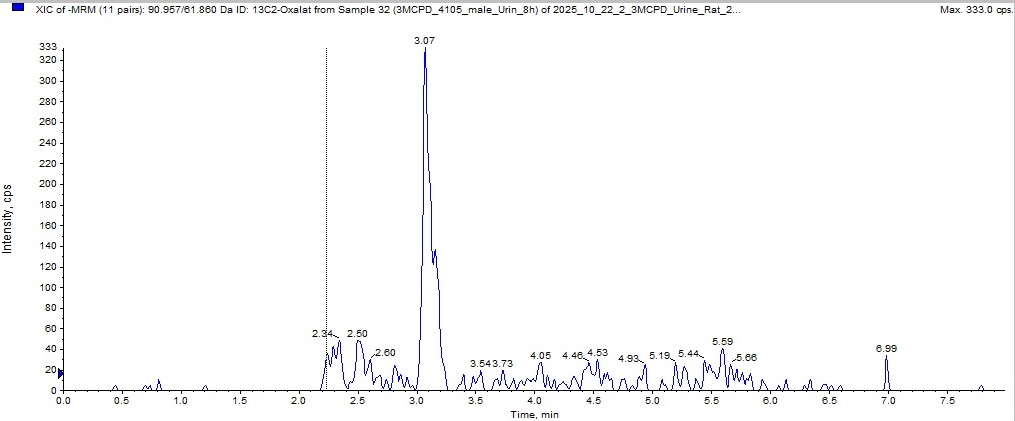 |
| --- |
| 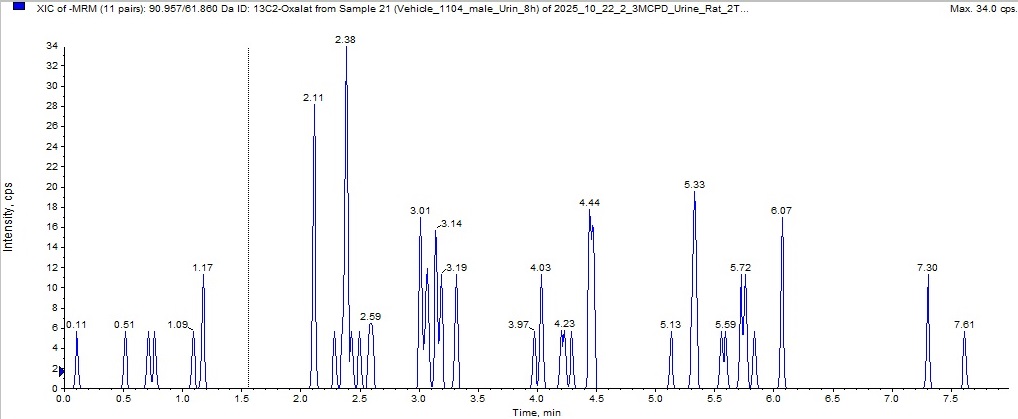 |
| **Figure S3**. Extracted ion chromatograms of [^13^C_2_]oxalate (*m/z* 91.0 → 62.0; upper panel) detected in a urine sample 8 h after administration of 50 mg [^13^C_3_]3-MCPD/kg bw. The same trace observed in pooled urine of control animals was devoid of any signal (lower panel). |

| 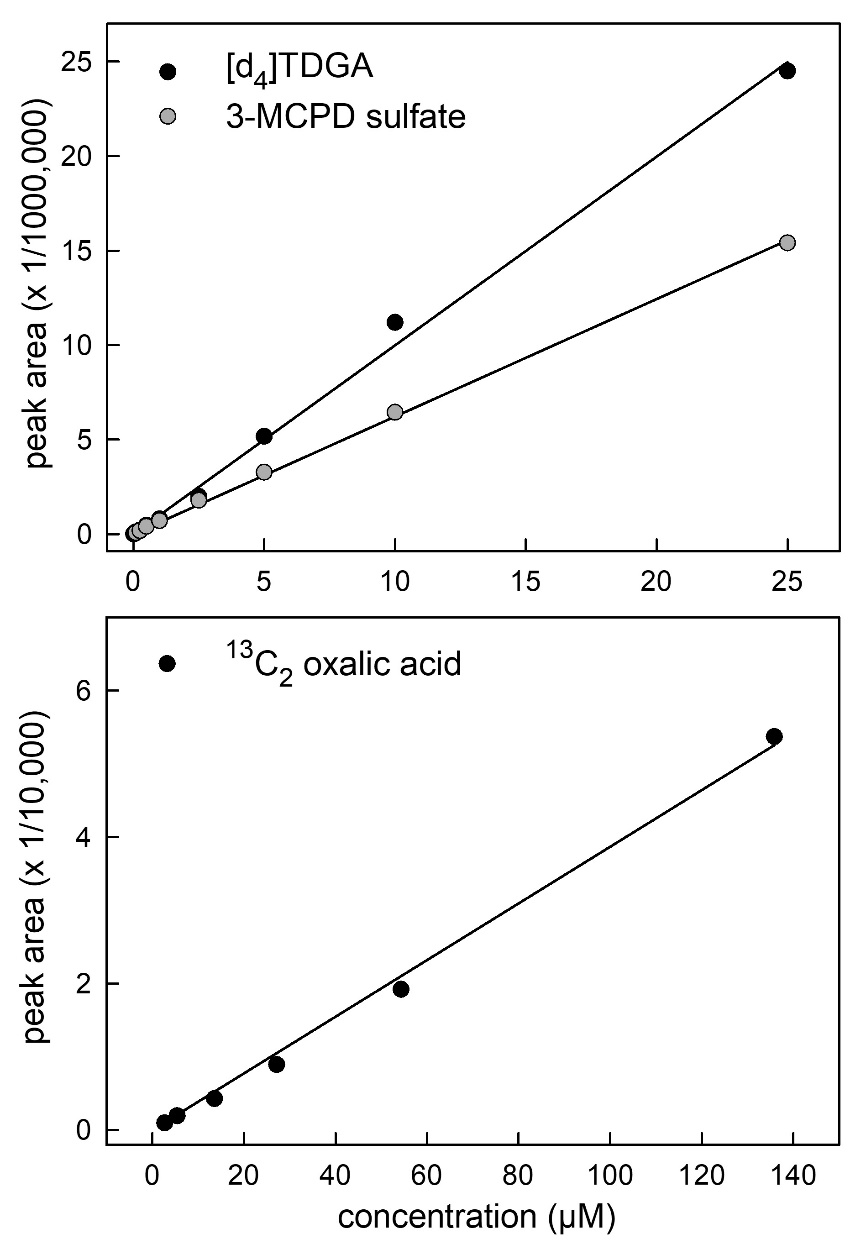 |
| --- |
| **Figure S4**. Upper panel: The linearity of detection was determined using 11 solutions of [d_4_]TDGA and of 3-MCPD sulfate (0.01 µM to 25 µM), which were prepared as mixtures in 380 µL eluent A (10 mM tributylamine and 0.25% acetic acid) and 20 µL of a pool of six blank rat urine samples. The data was fitted with a trend line by least-squares linear regression (R^2^ = 0.997 for TDGA and R^2^ > 0.999 for 3-MCPD sulfate). [d_4_]TDGA was used because rat urine samples are not completely free of TDGA. Lower panel: The linearity of detection was determined using six dilutions of [^13^C_2_]oxalic acid prepared in a pool of six blank rat urine samples (2.73 µM to 136 µM), which were processed as described. The data was fitted with a trend line by least-squares linear regression (R^2^ = 0.998). |

| 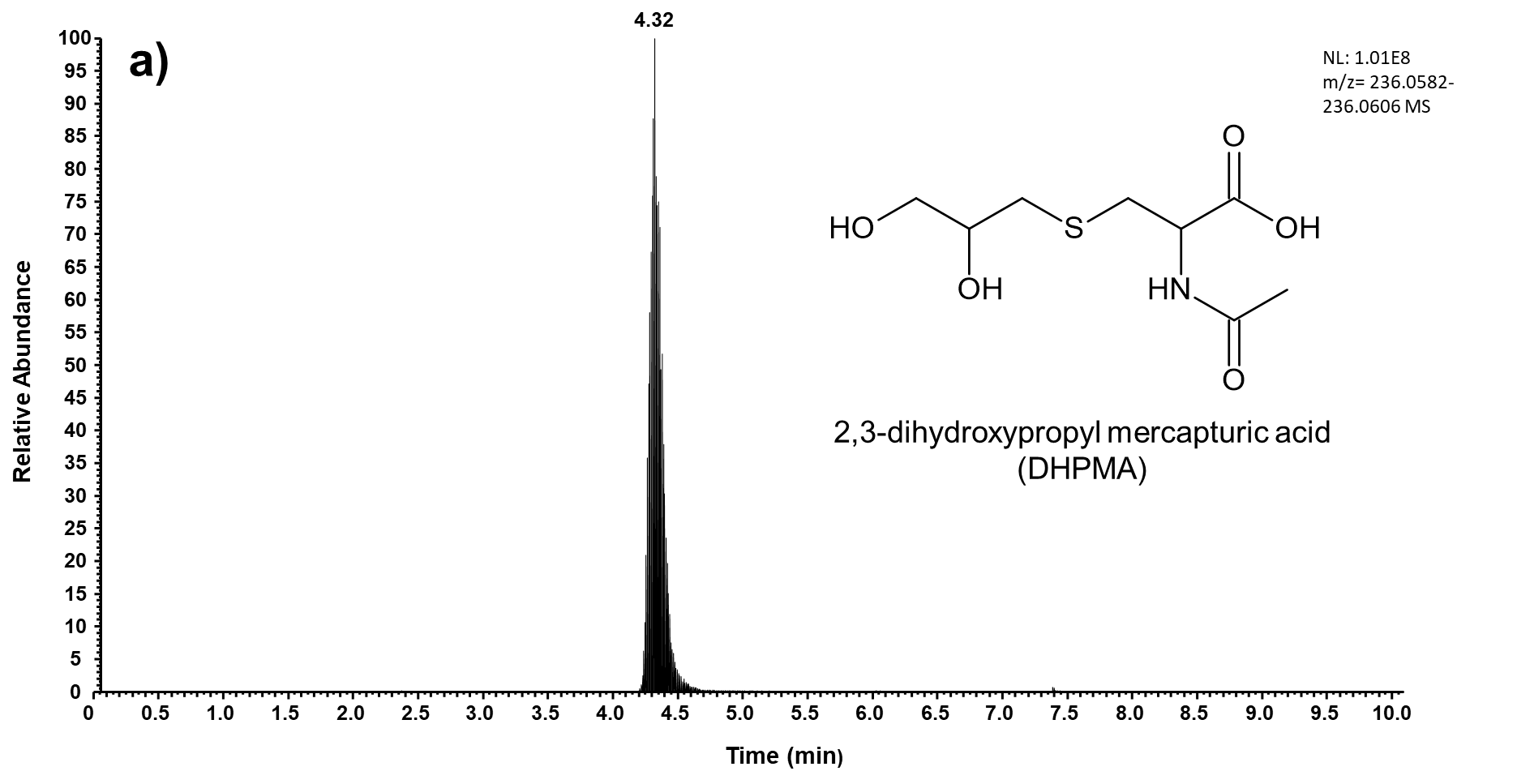 |
| --- |
| 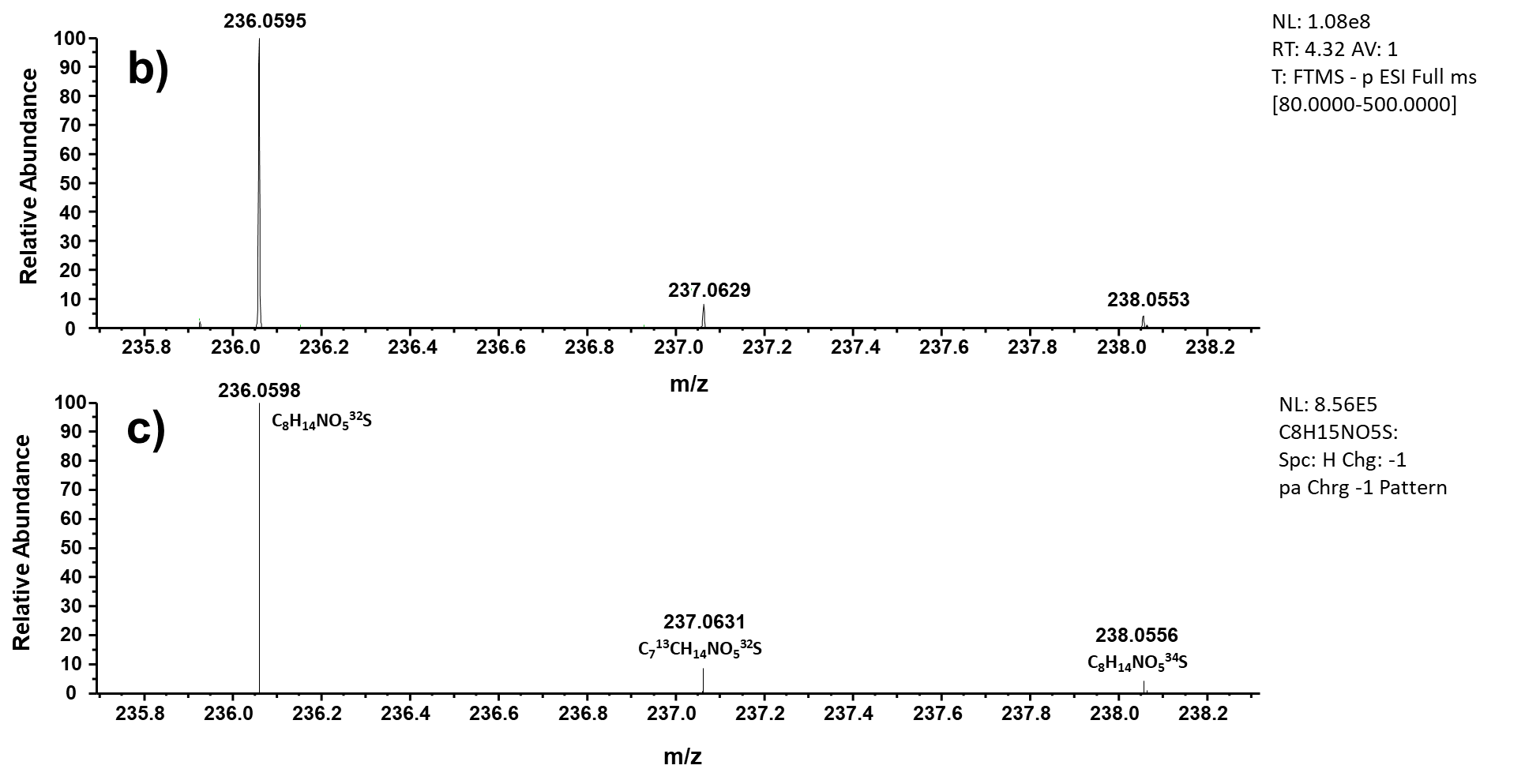 |
| 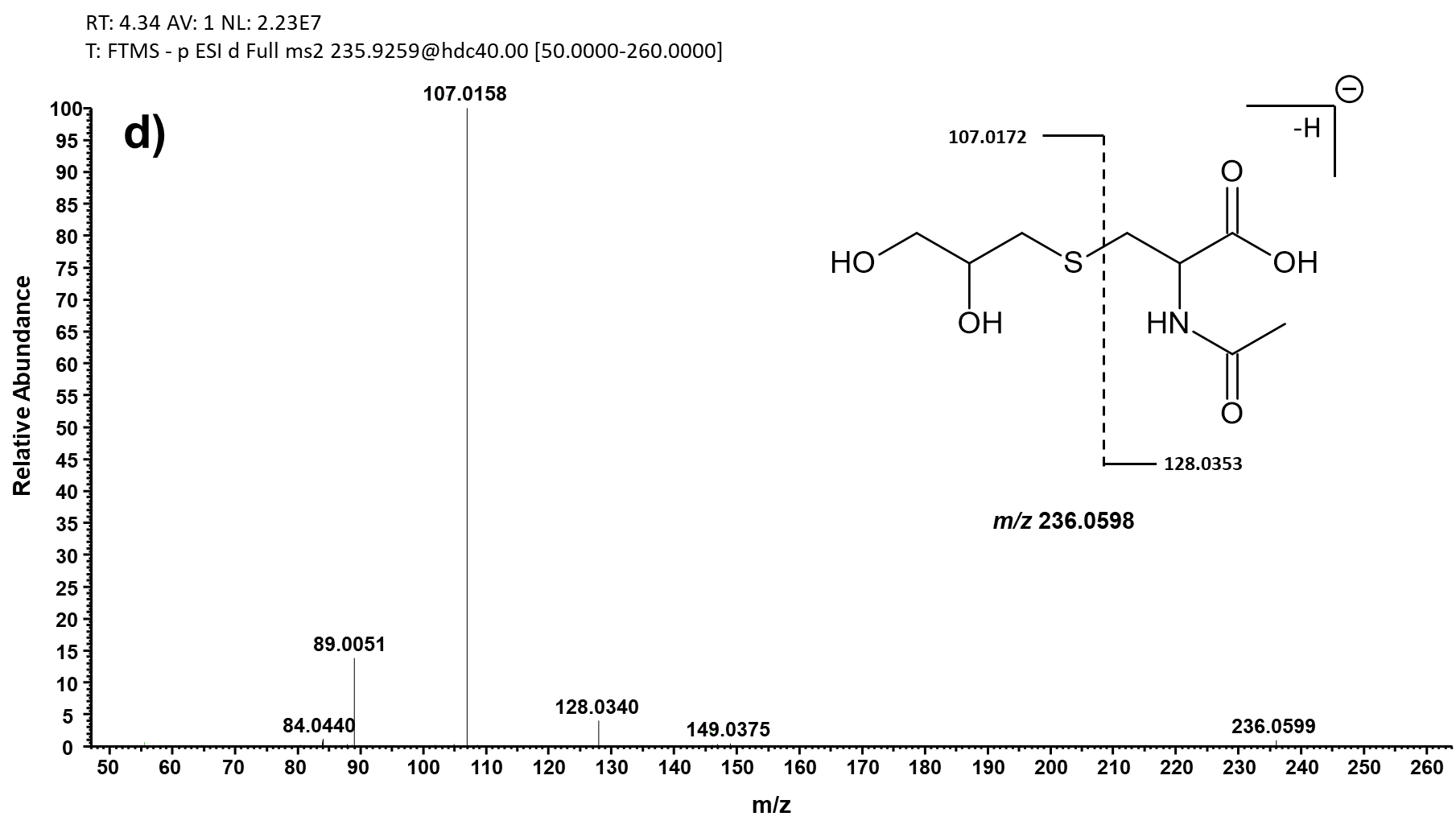 |
| **Figure S5**. HR-MS identification of 2,3-dihydroxypropyl mercapturic acid (DHPMA): extracted ion chromatogramm (a), experimental (b) and theoretical (c) isotopic patterns, and MS2 spectrum (d). |
| 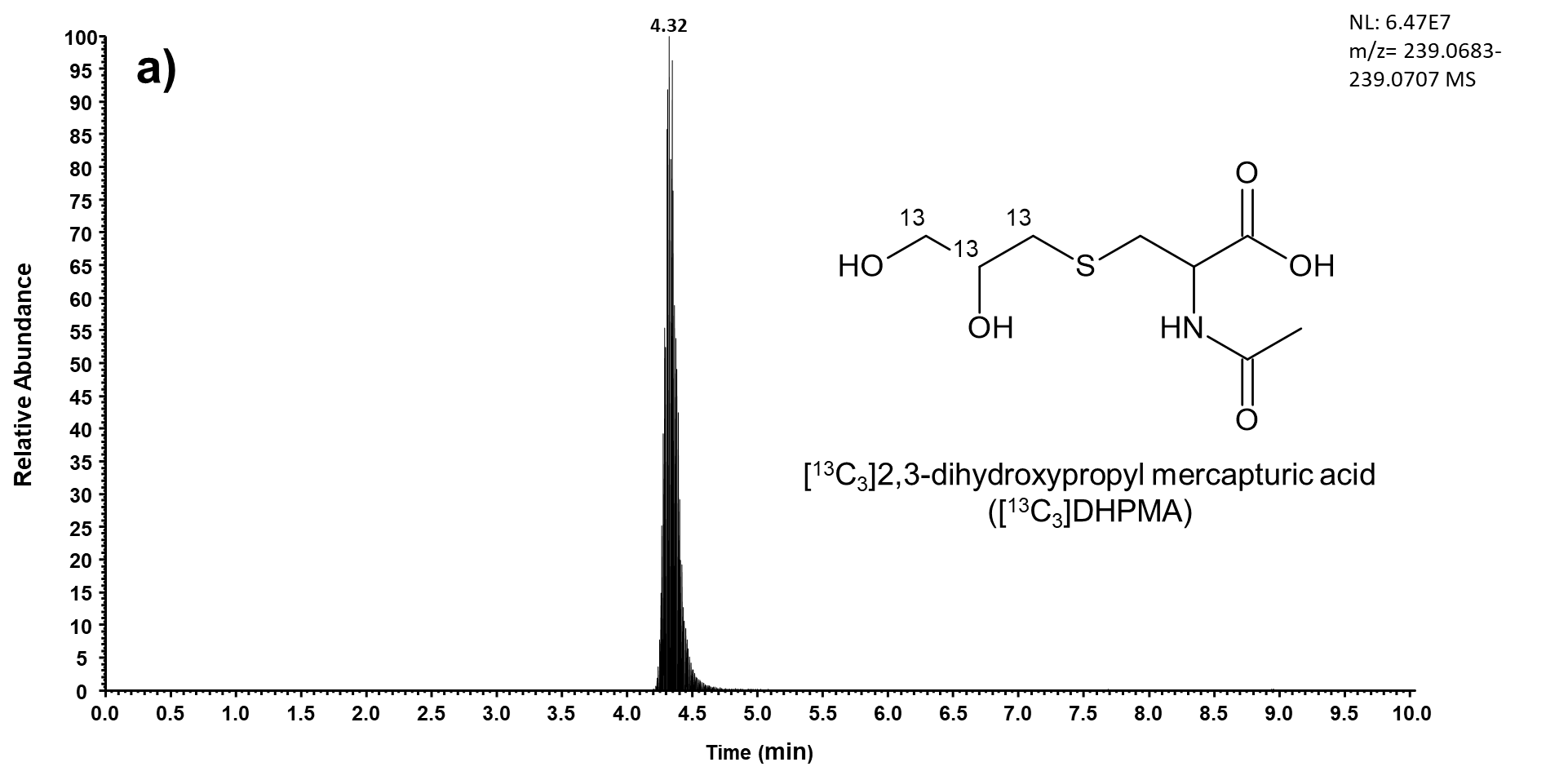 |
| 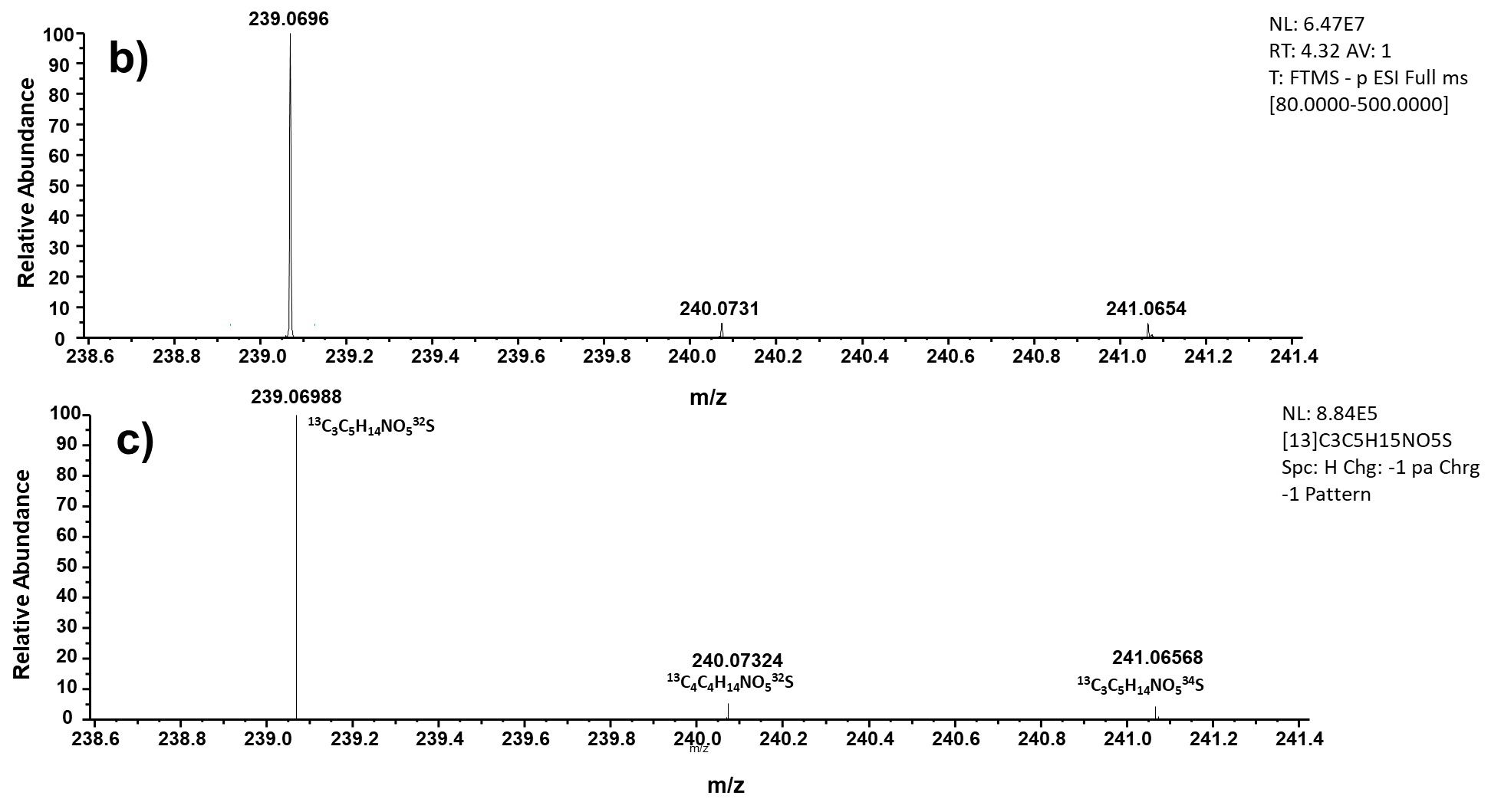 |
| 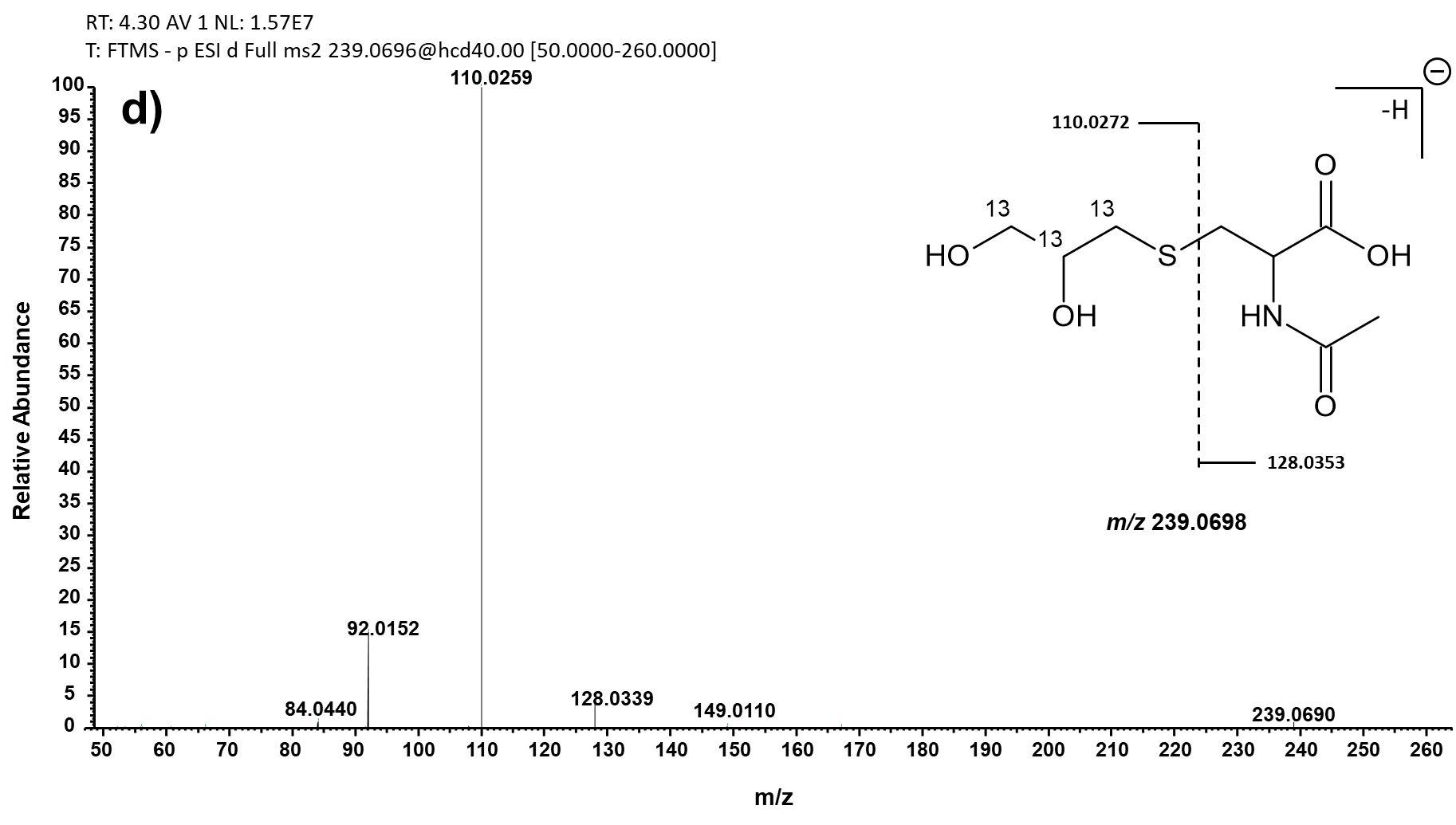 |
| **Figure S6**. HR-MS identification of [^13^C_3_]2,3-dihydroxypropyl mercapturic acid ([^13^C_3_]DHPMA): extracted ion chromatogramm (a), experimental (b) and theoretical (c) isotopic patterns, and MS2 spectrum (d). |

| 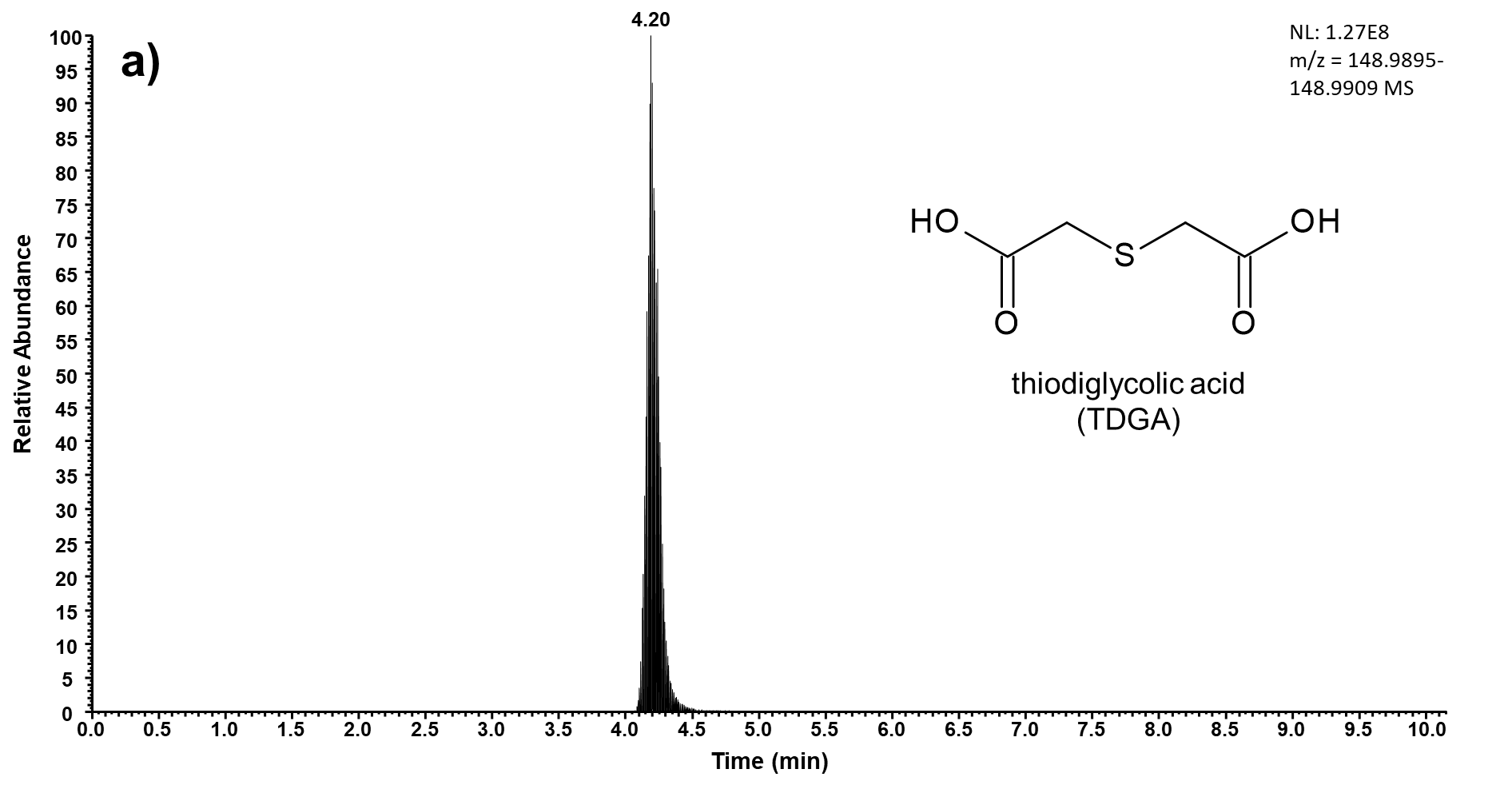 |
| --- |
| 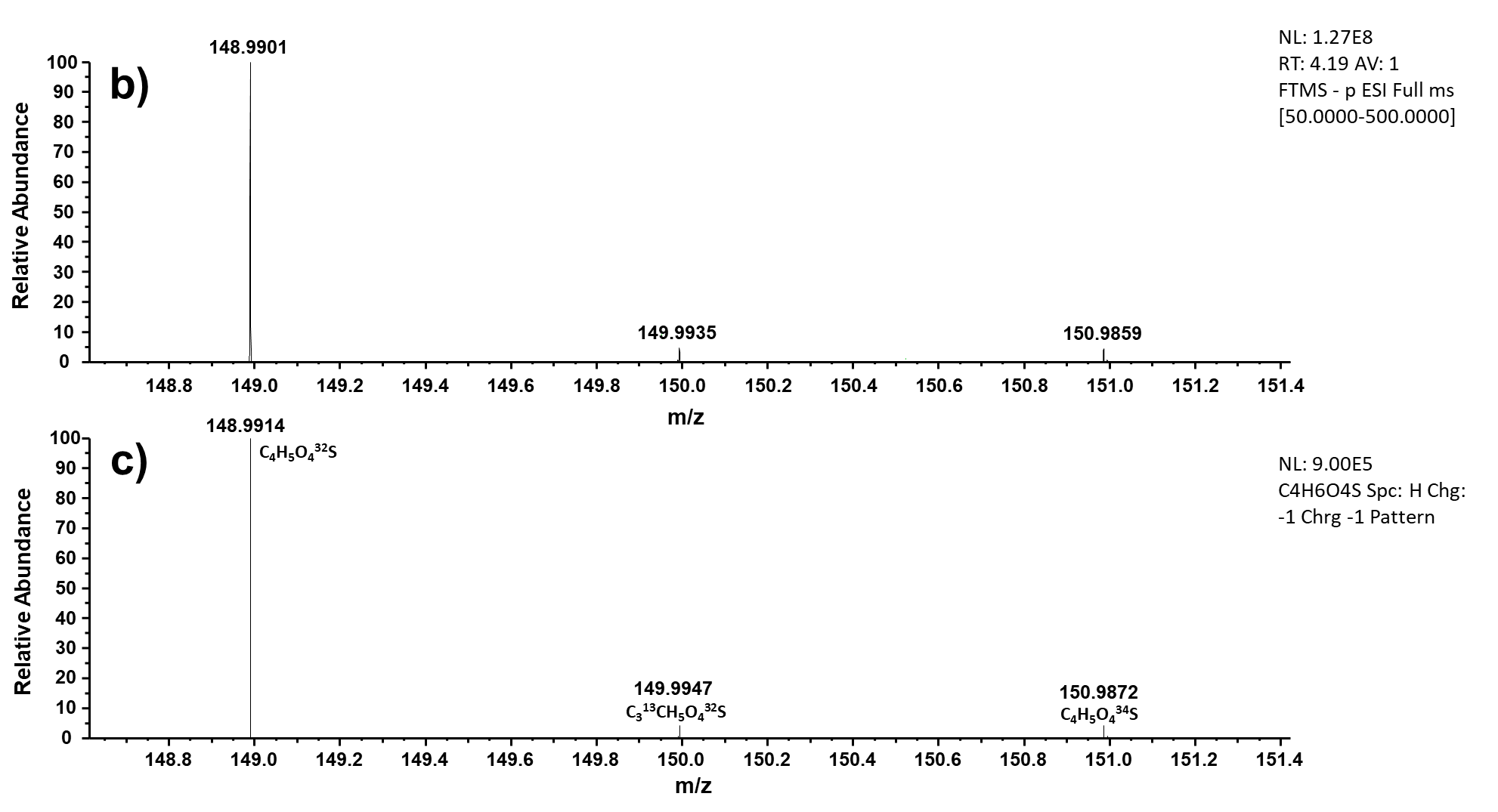 |
| 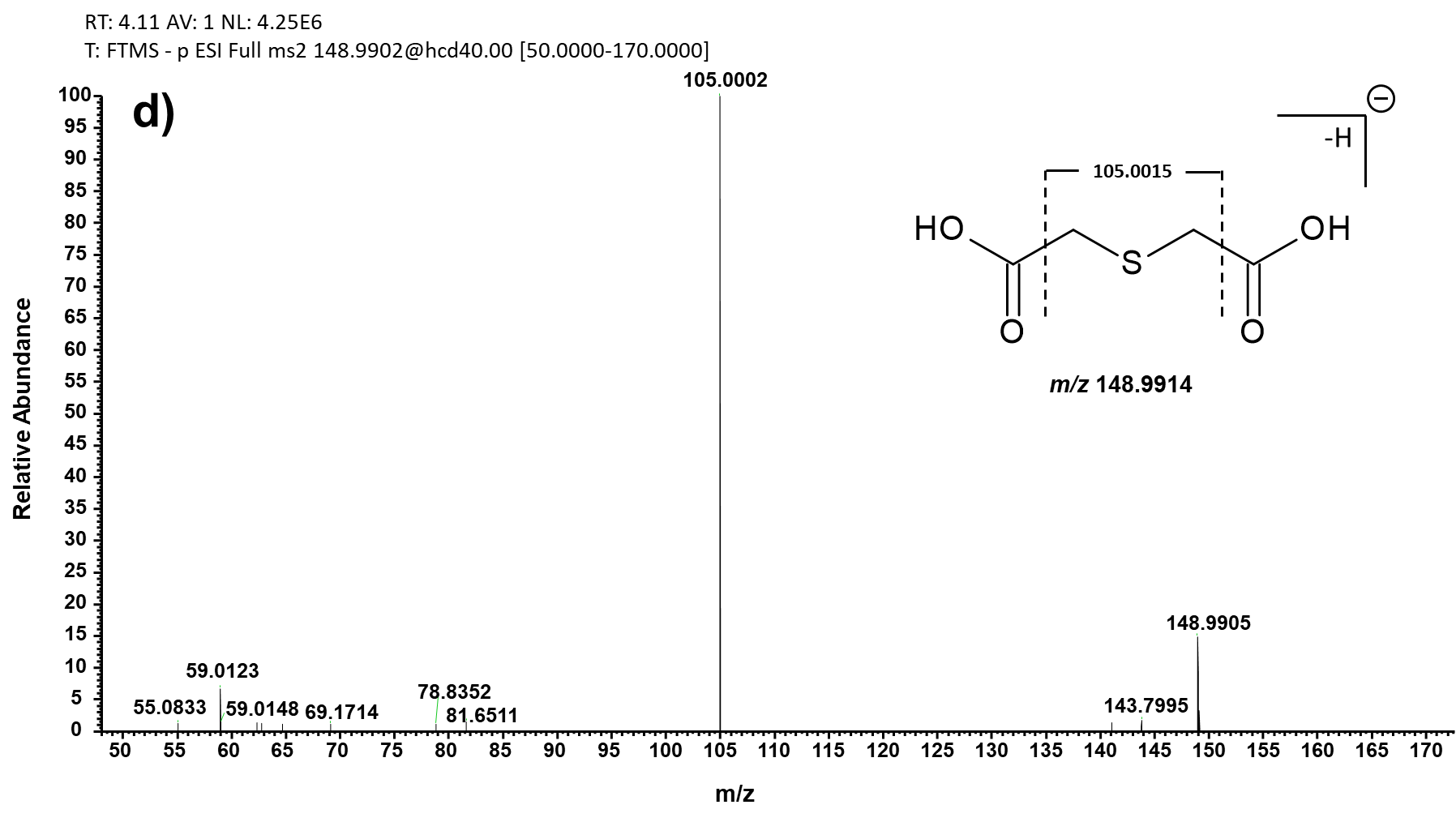 |
| **Figure S7**. HR-MS identification of thiodiglycolic acid (TDGA): extracted ion chromatogramm (a), experimental (b) and theoretical (c) isotopic patterns, and MS2 spectrum (d). |

| 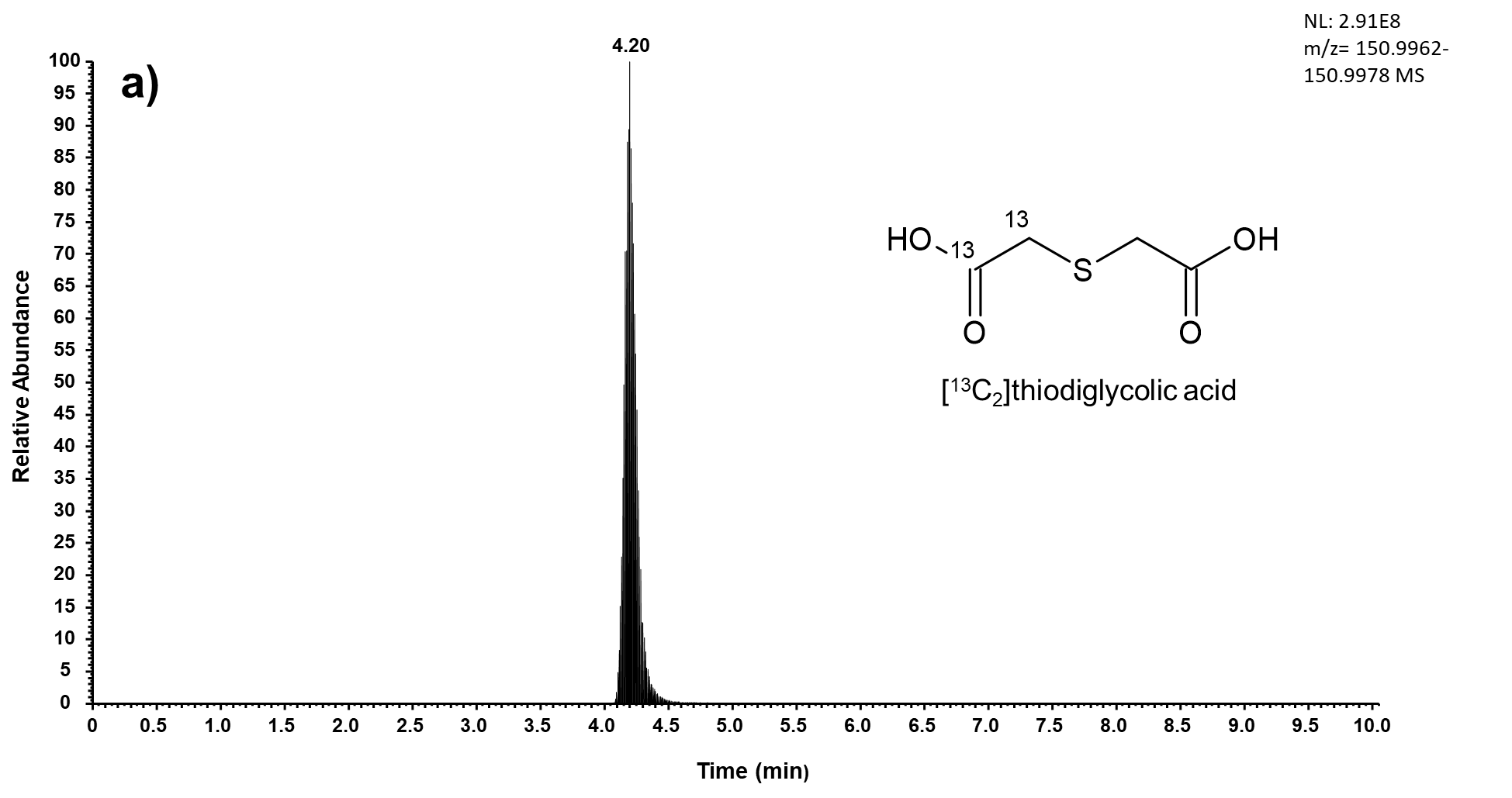 |
| --- |
| 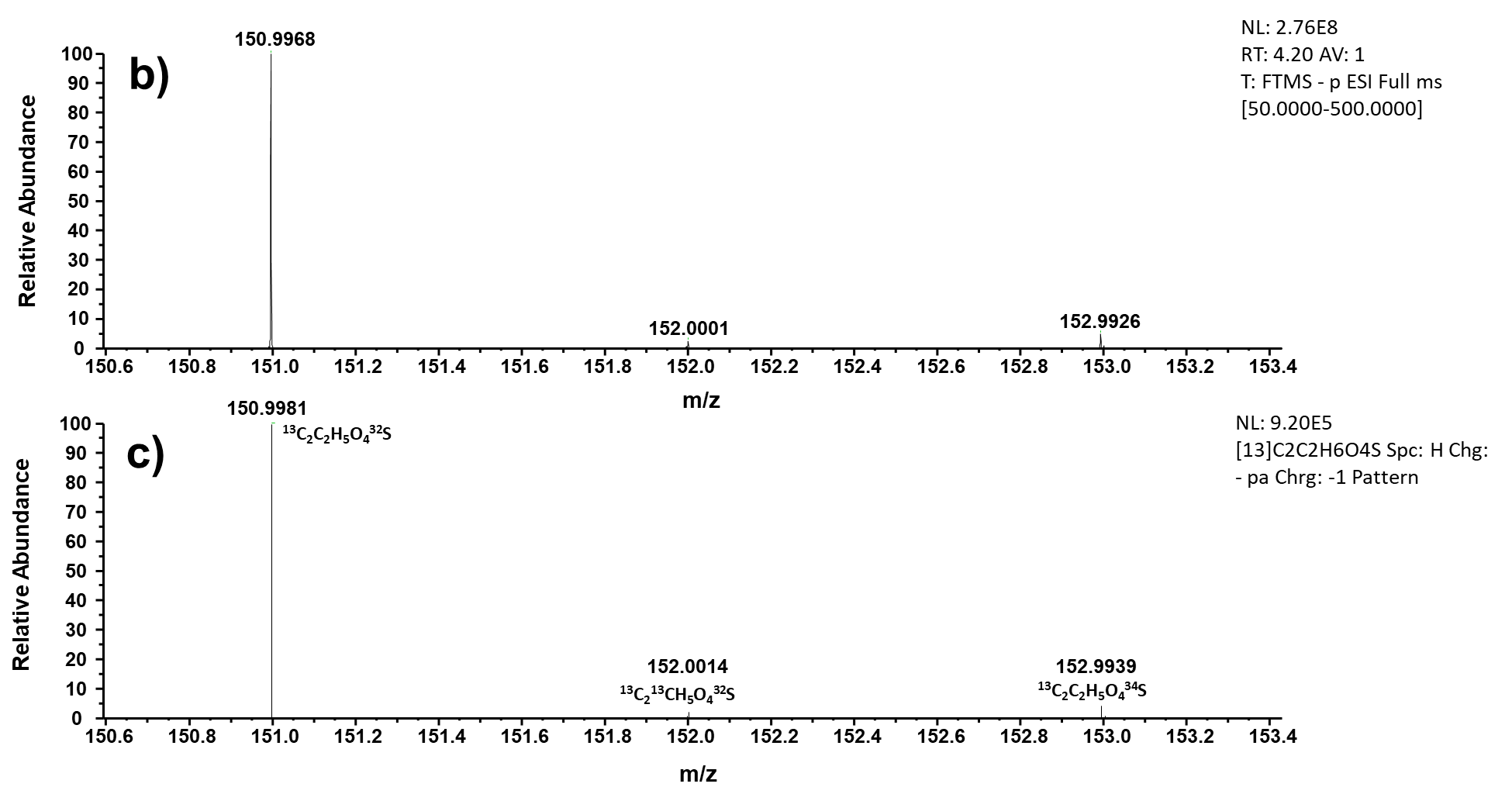 |
| 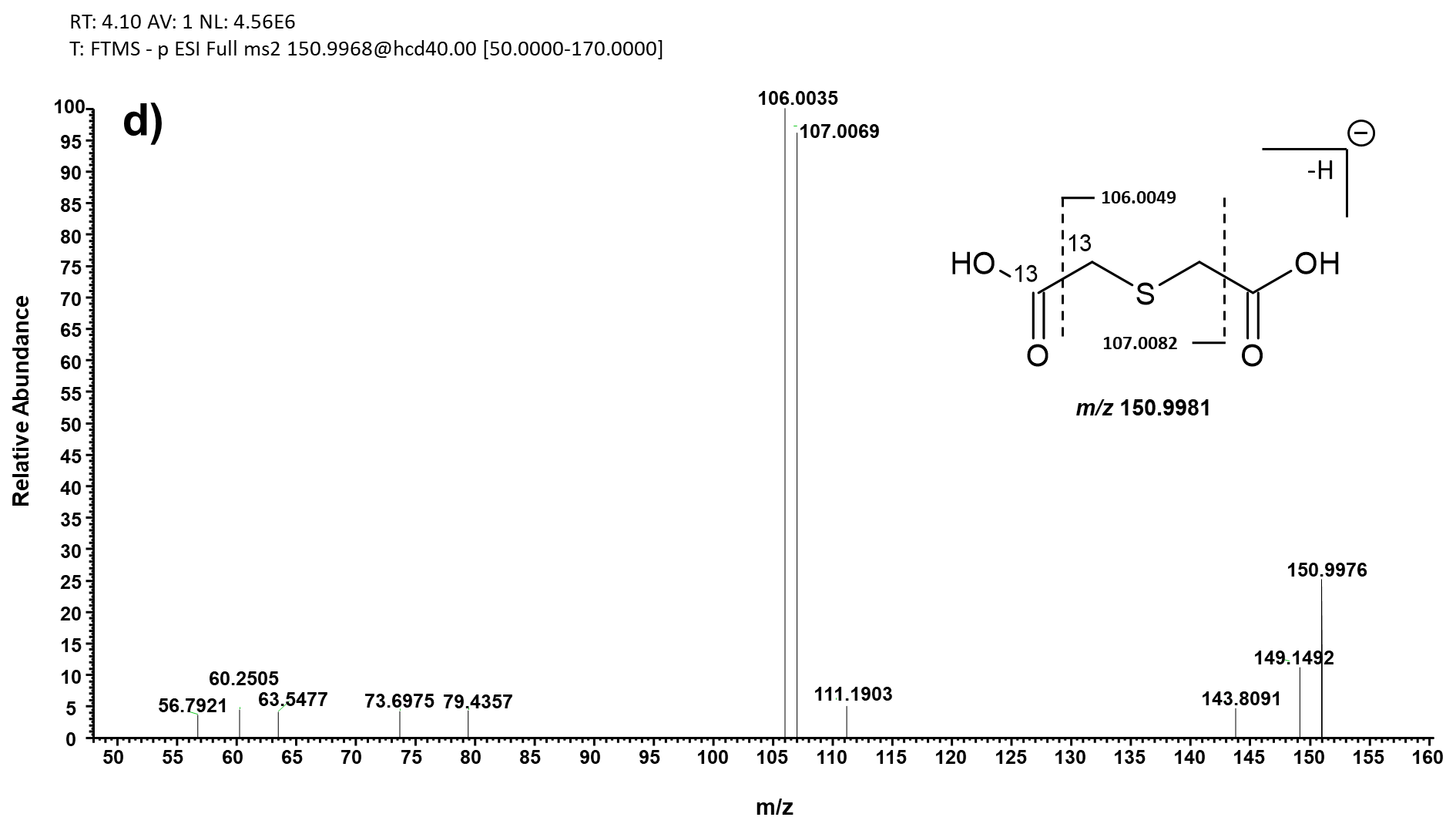 |
| **Figure S8.** HR-MS identification of [^13^C_2_]thiodiglycolic acid ([^13^C_2_]TDGA): extracted ion chromatogramm (a), experimental (b) and theoretical (c) isotopic patterns, and MS2 spectrum (d). |

| 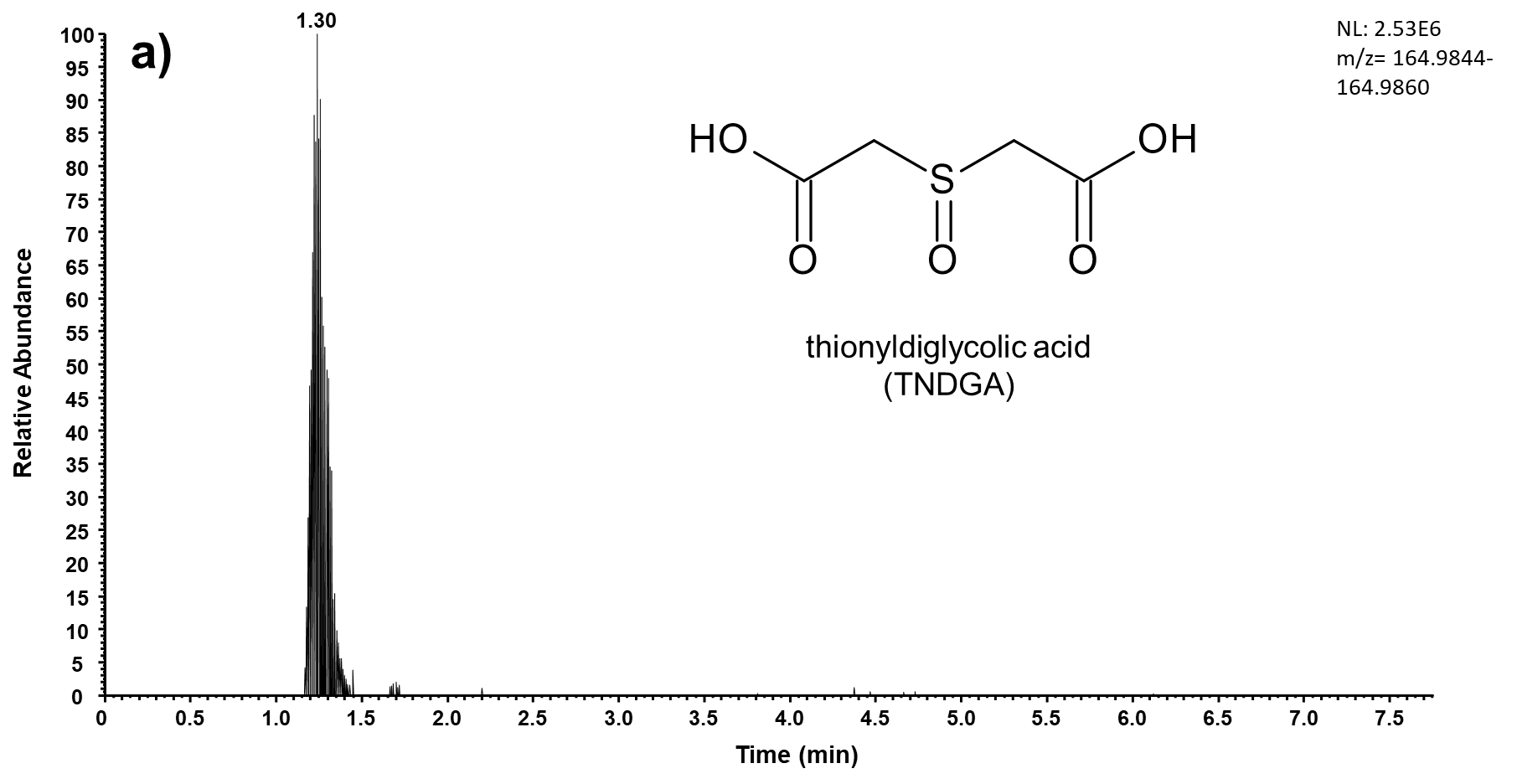 |
| --- |
| 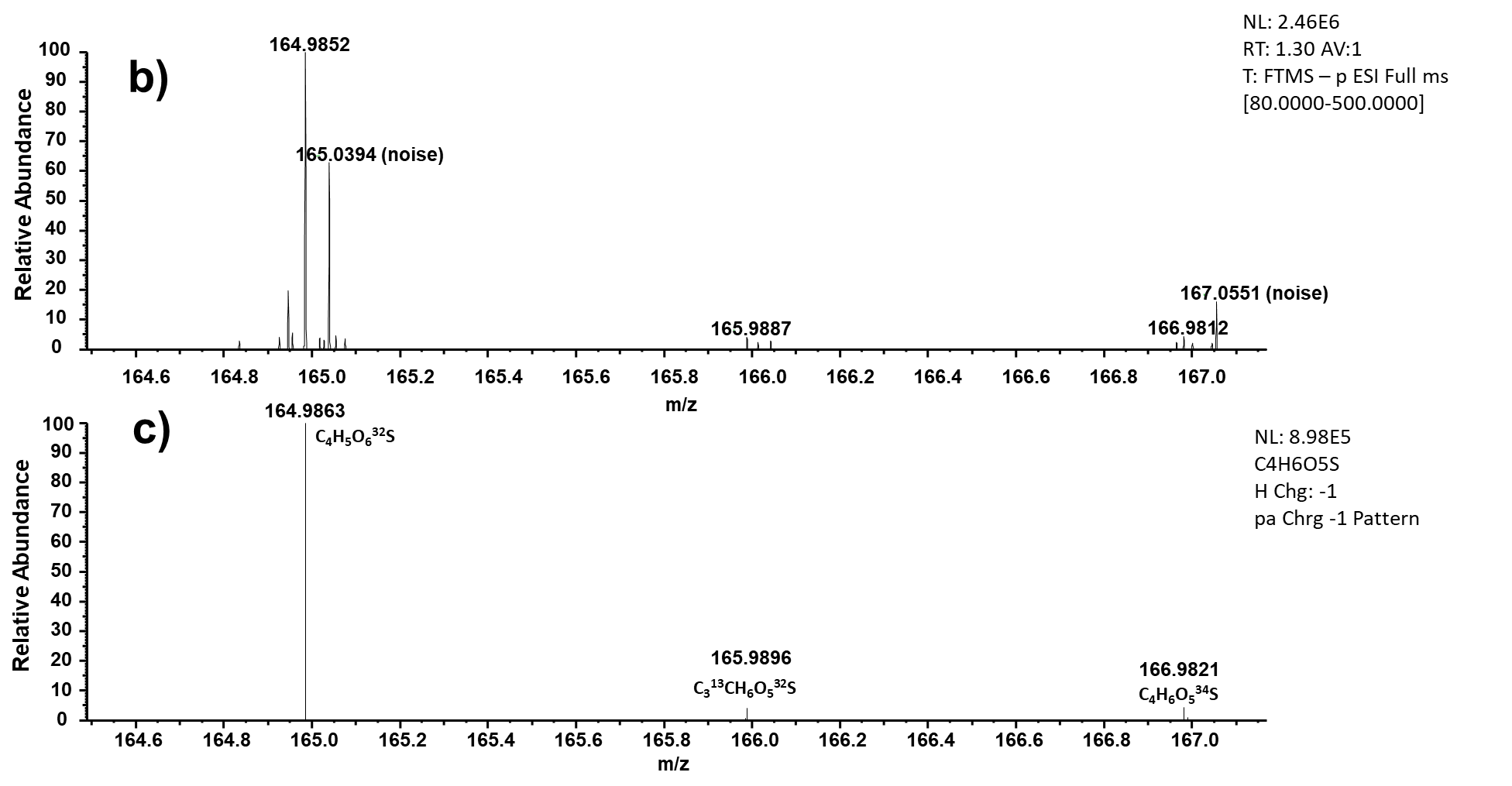 |
| **Figure S9**. HR-MS identification of thionyldiglycolic acid: extracted ion chromatogramm (a), as well as experimental (b) and theoretical (c) isotopic patterns. Due to the low urinary concentration an MS2 spectrum was not recorded. |

| 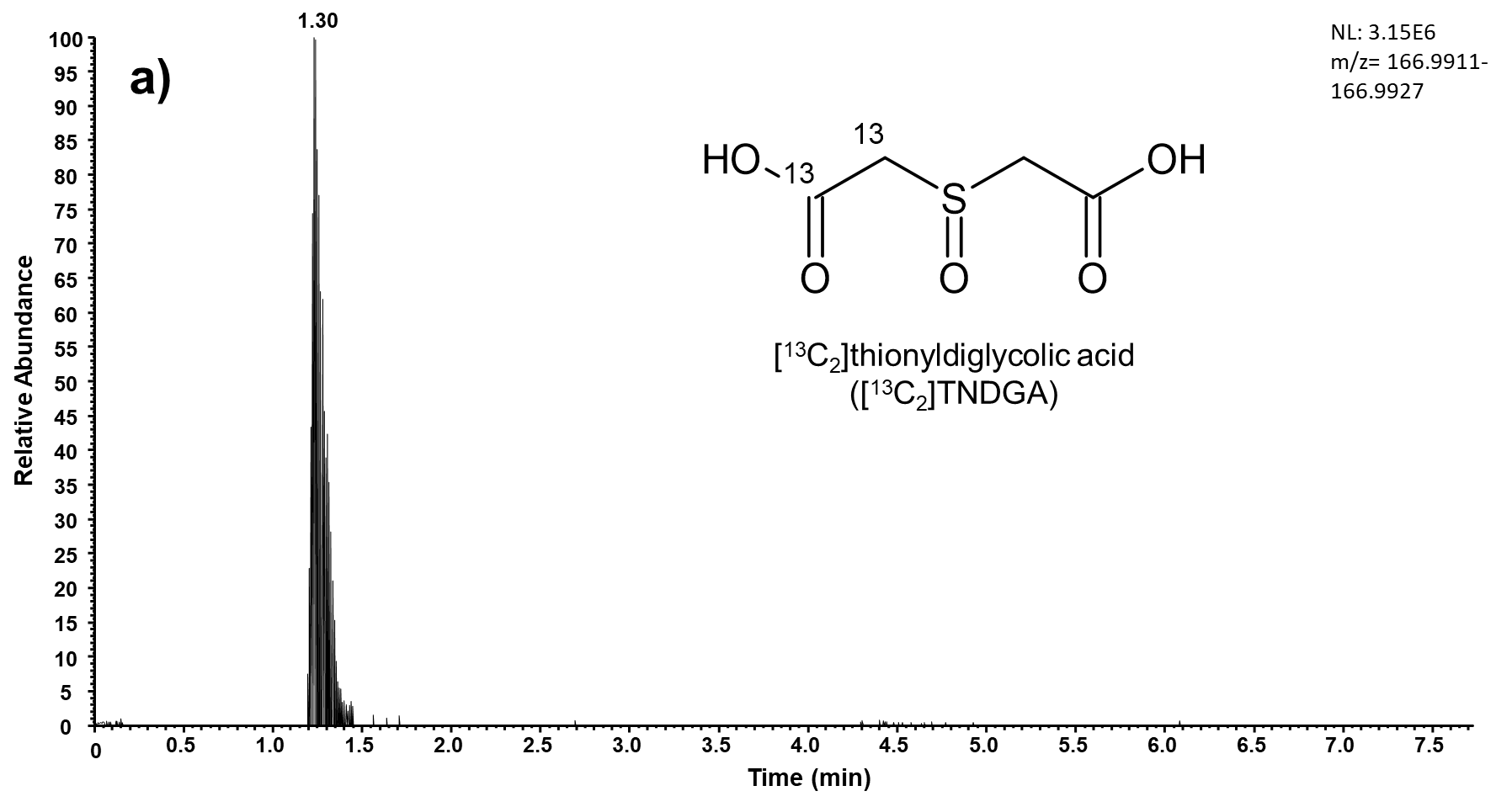 |
| --- |
| 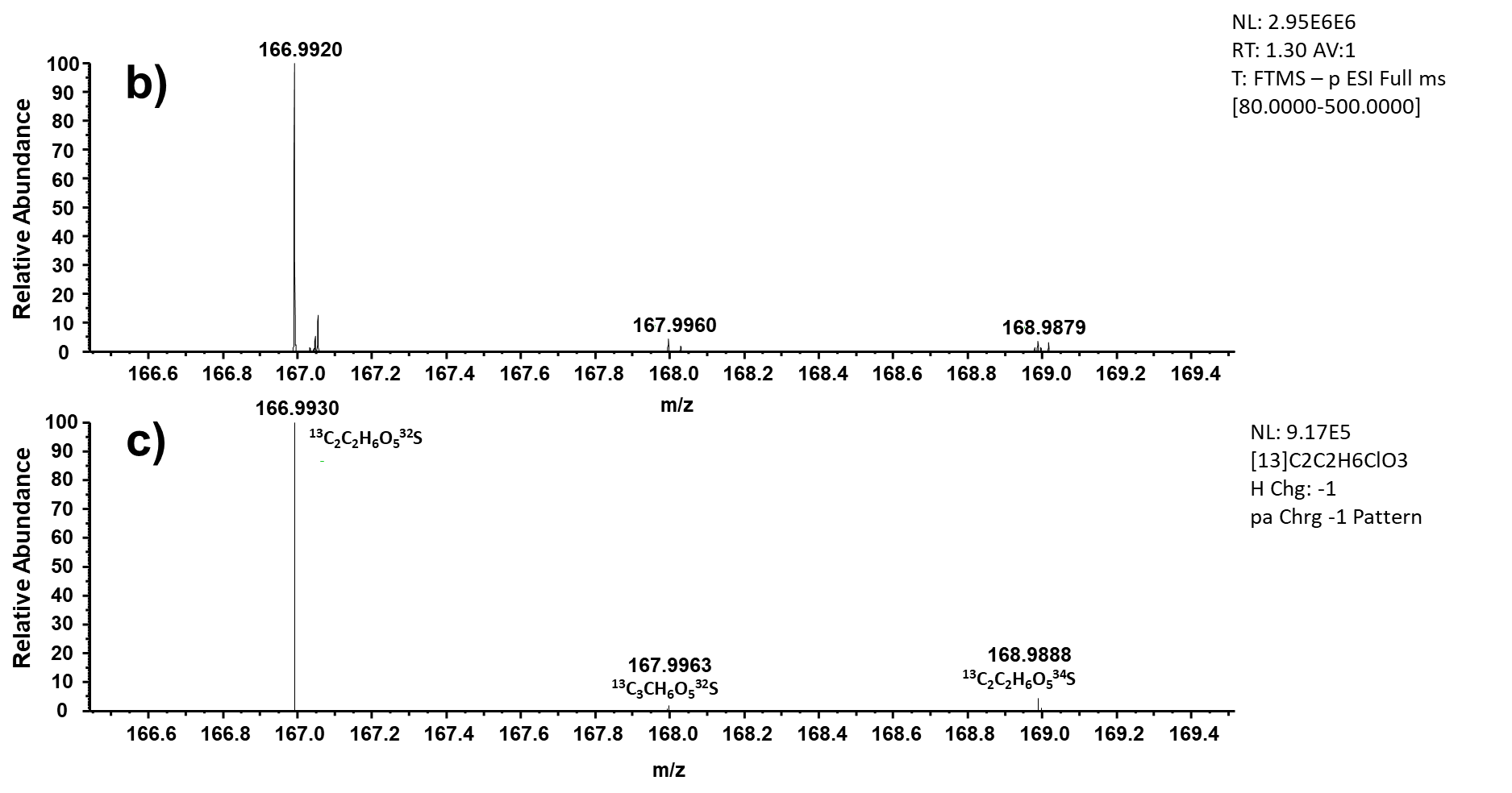 |
| **Figure S10**. HR-MS identification of [^13^C_2_]thionyldiglycolic acid: extracted ion chromatogramm (a), as well as experimental (b) and theoretical (c) isotopic patterns. Due to the low urinary concentration an MS2 spectrum was not recorded. |

| 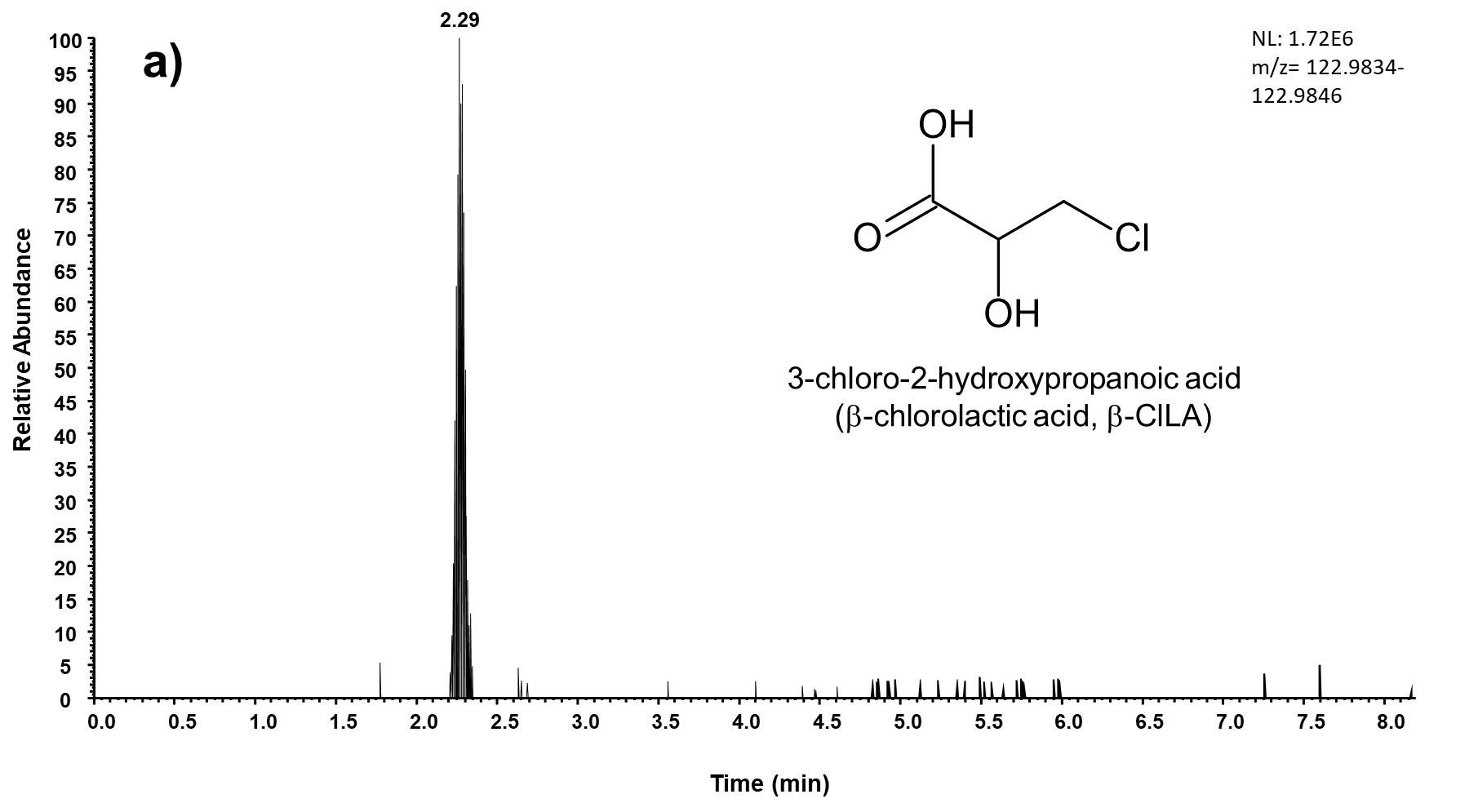 |
| --- |
| 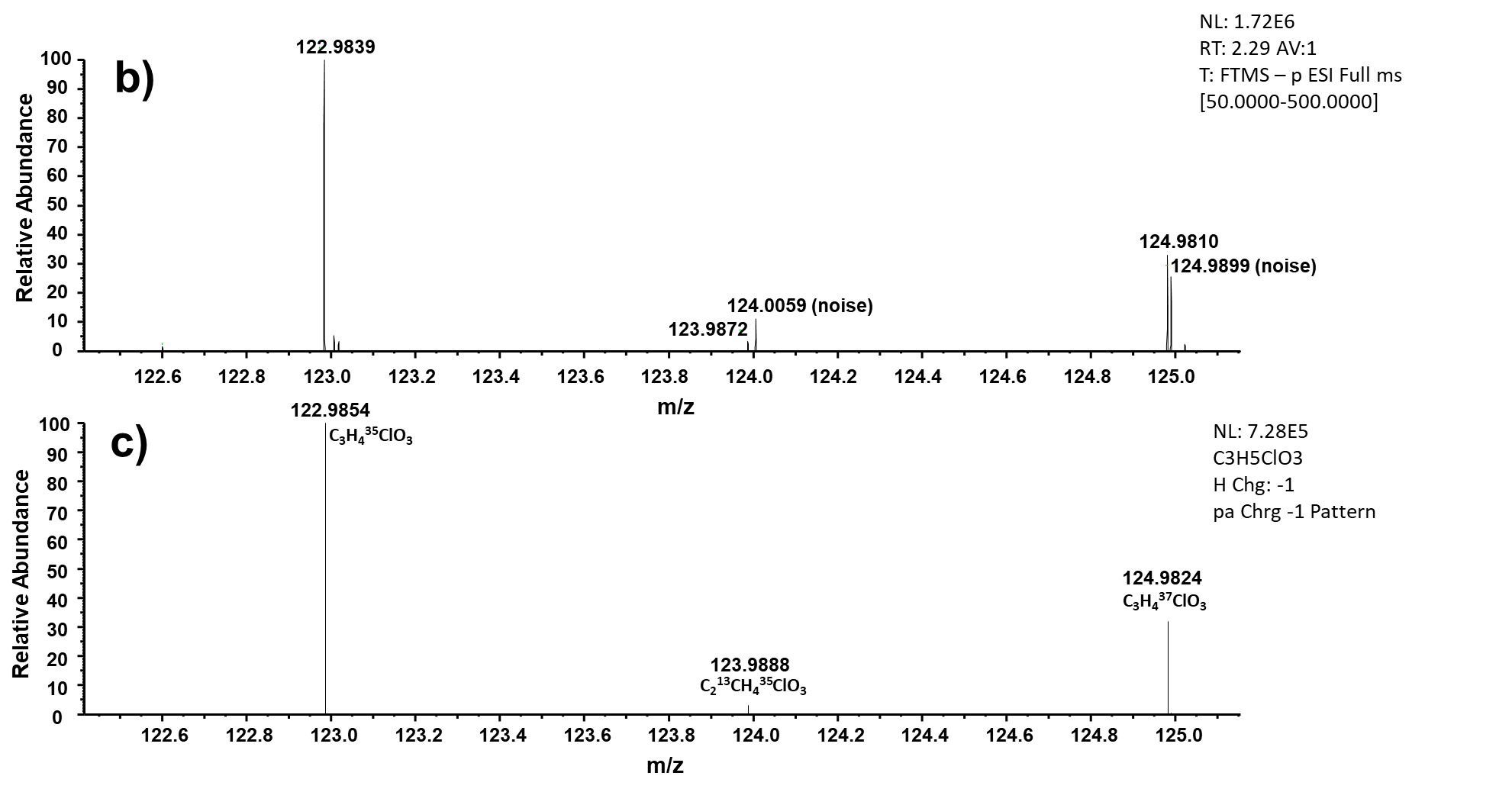 |
| **Figure S11**. HR-MS identification of β-chlorolactic acid (β-ClLA): extracted ion chromatogramm (a), as well as experimental (b) and theoretical (c) isotopic patterns. Due to the low urinary concentration an MS2 spectrum was not recorded. |

| 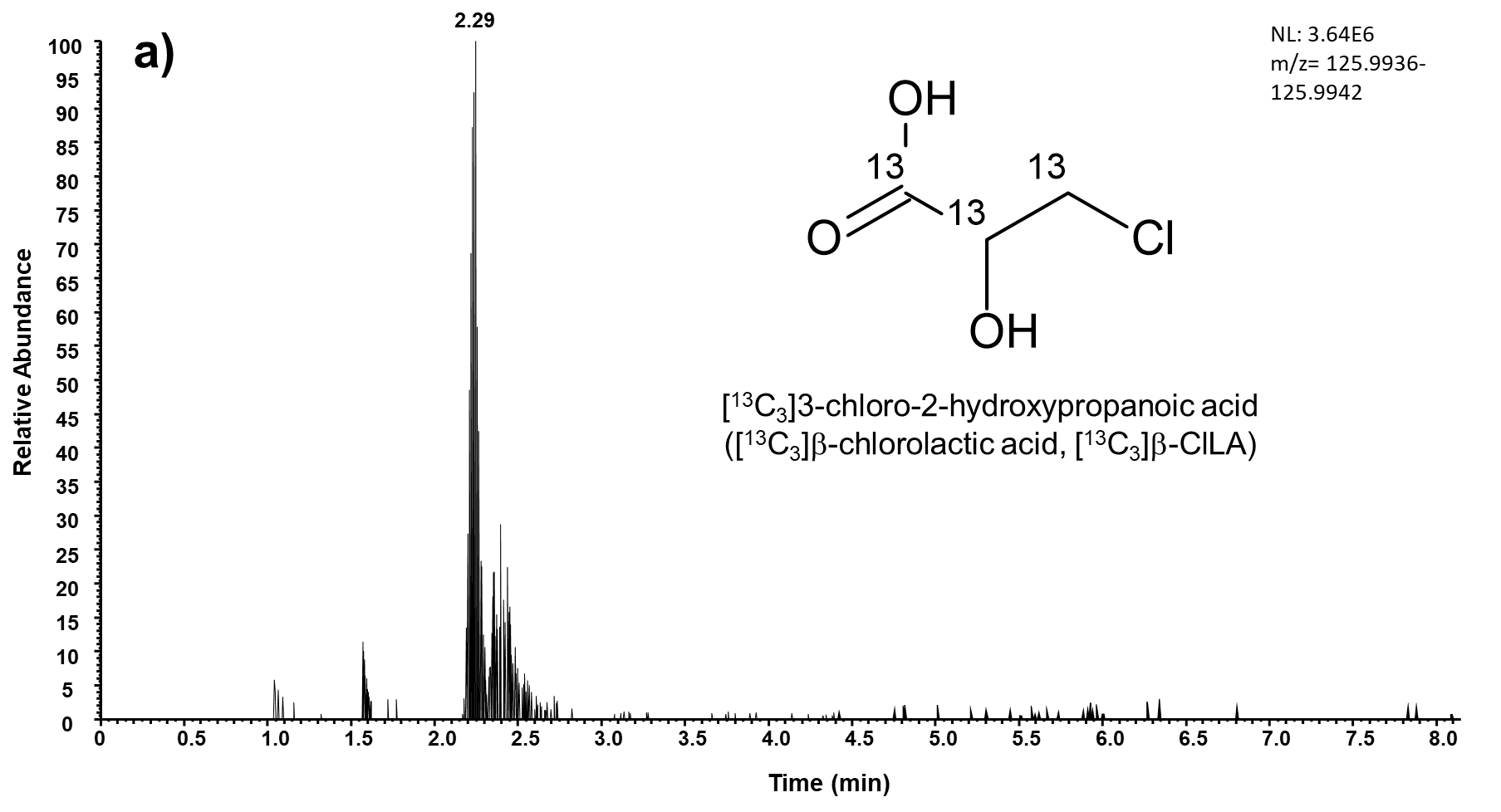 |
| --- |
| 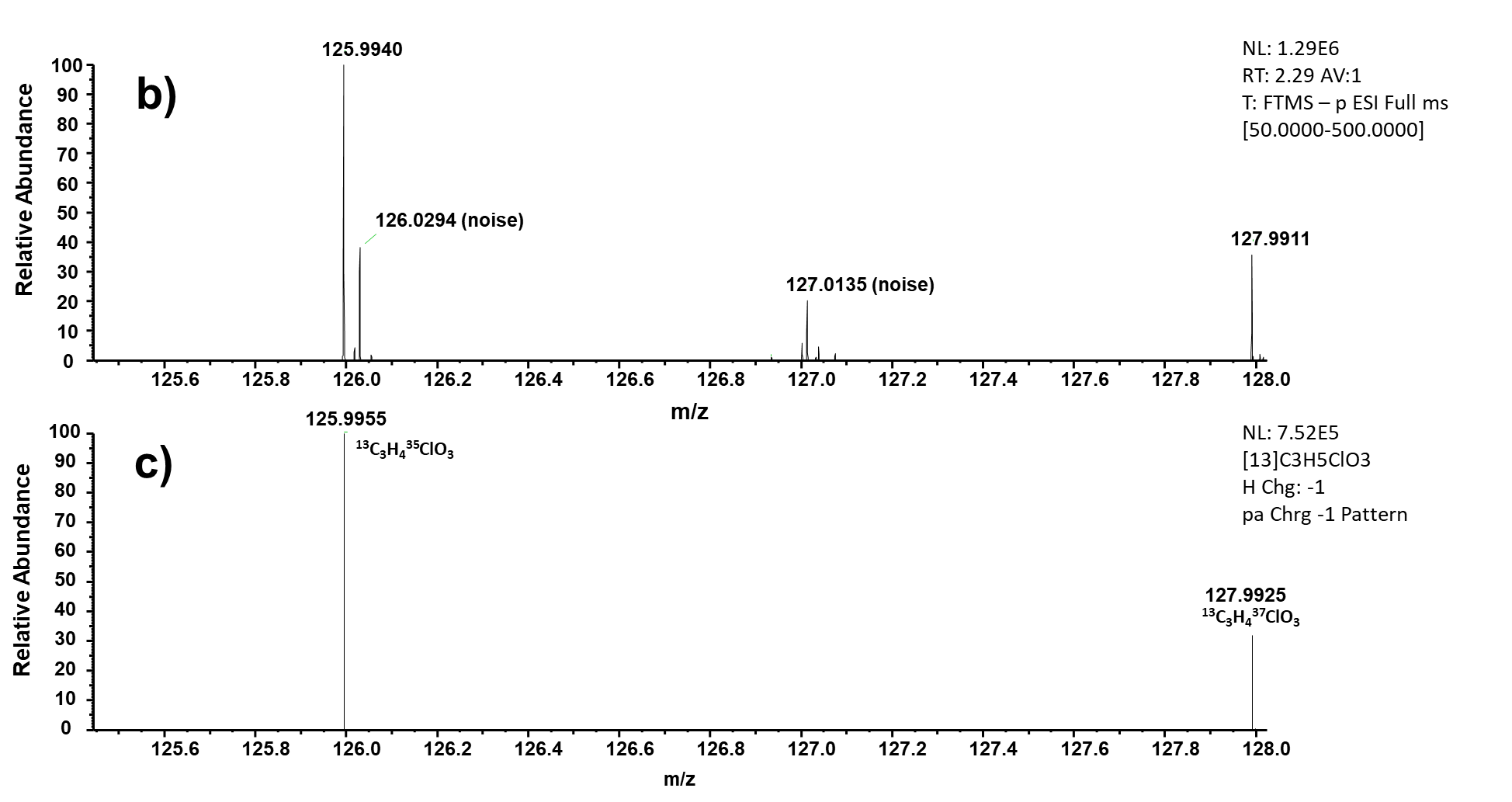 |
| **Figure S12**. HR-MS identification of [^13^C_3_]β-chlorolactic acid ([^13^C_3_]β-ClLA): extracted ion chromatogramm (a), as well as experimental (b) and theoretical (c) isotopic patterns. Due to the low urinary concentration an MS2 spectrum was not recorded. |

| 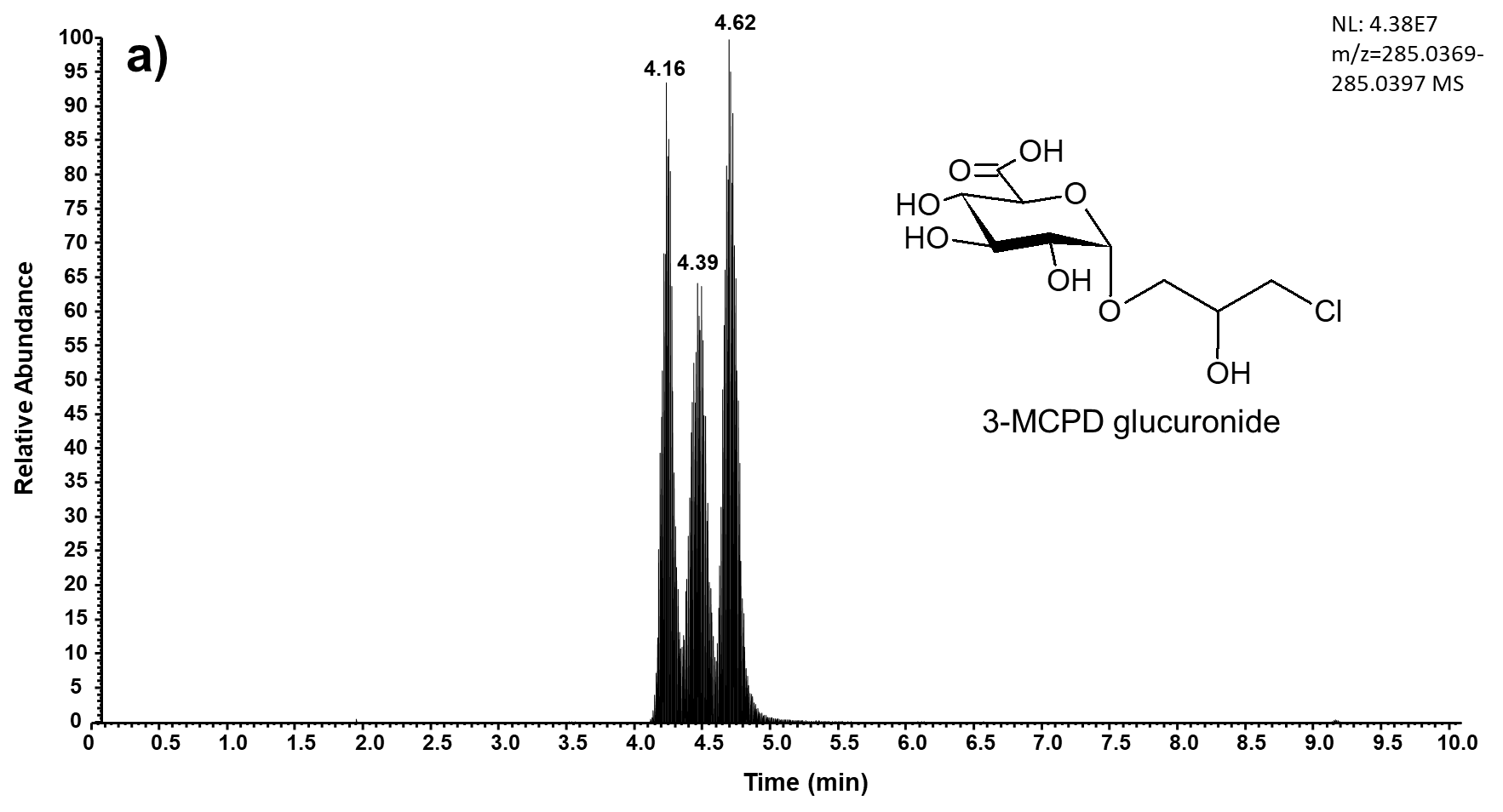 |
| --- |
| 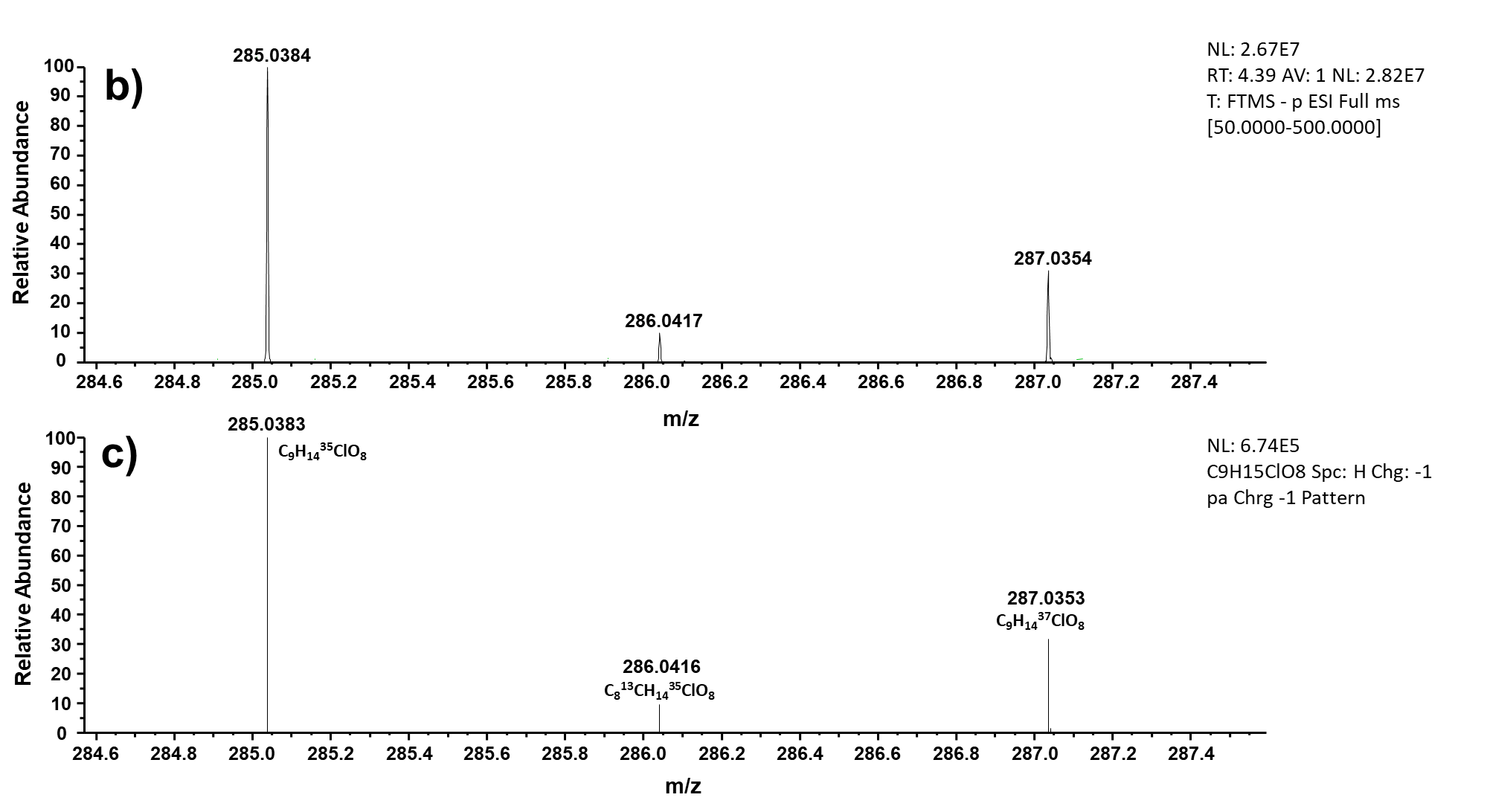 |
| 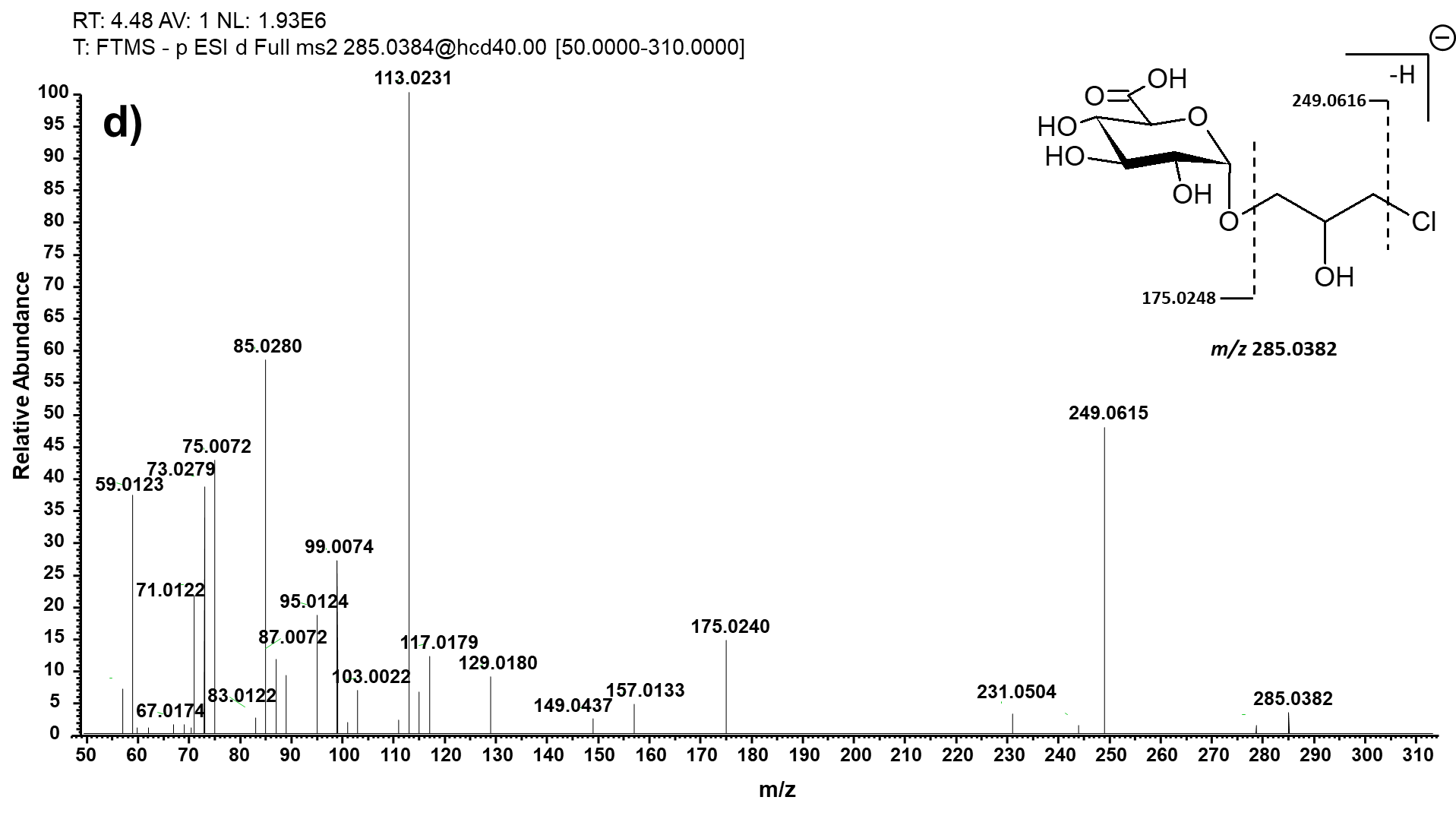 |
| **Figure S13**. HR-MS identification of 3-MCPD glucuronide: extracted ion chromatogramm (a), experimental (b) and theoretical (c) isotopic patterns, and MS2 spectrum (d). |
| 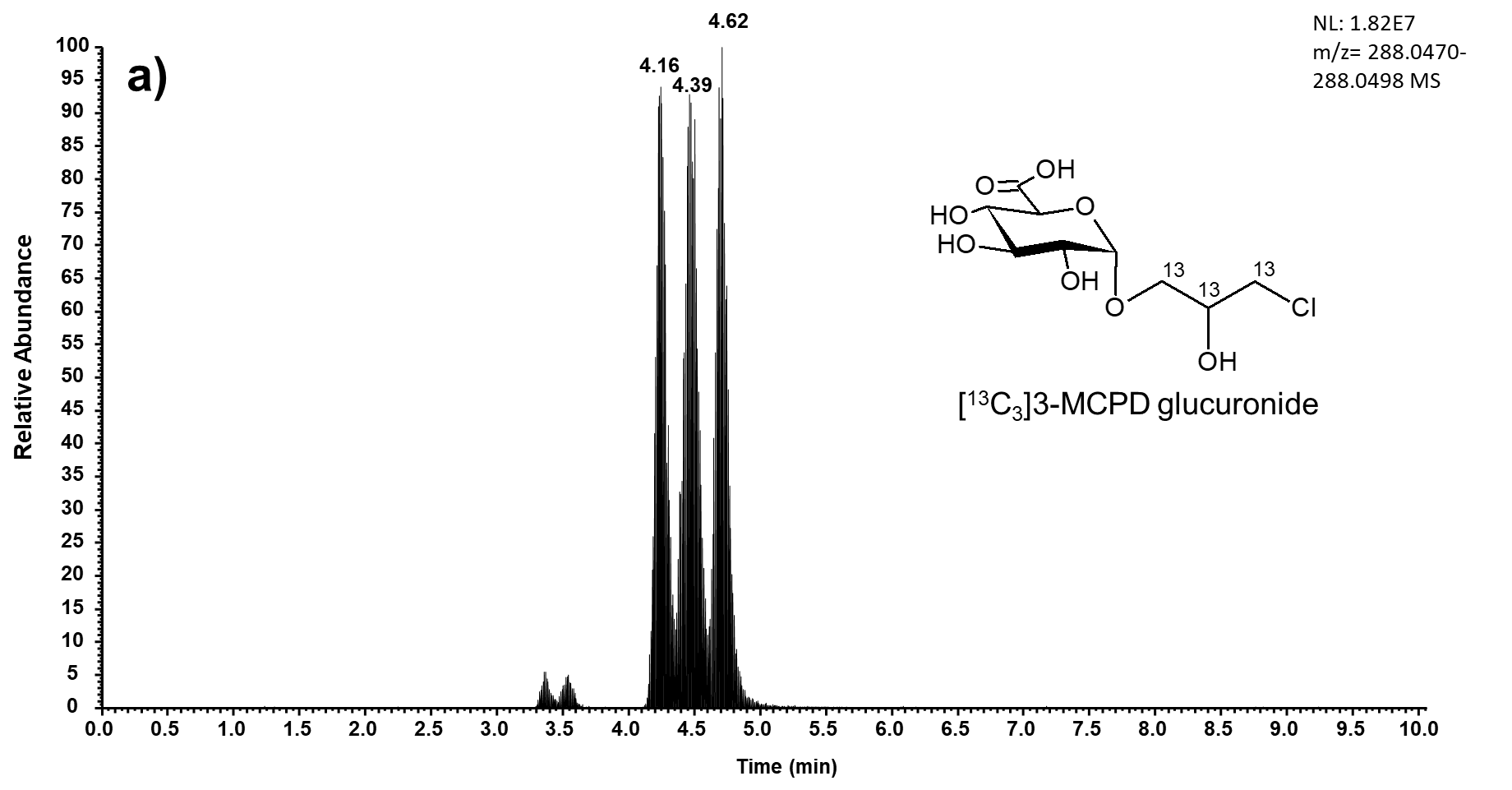 |
| 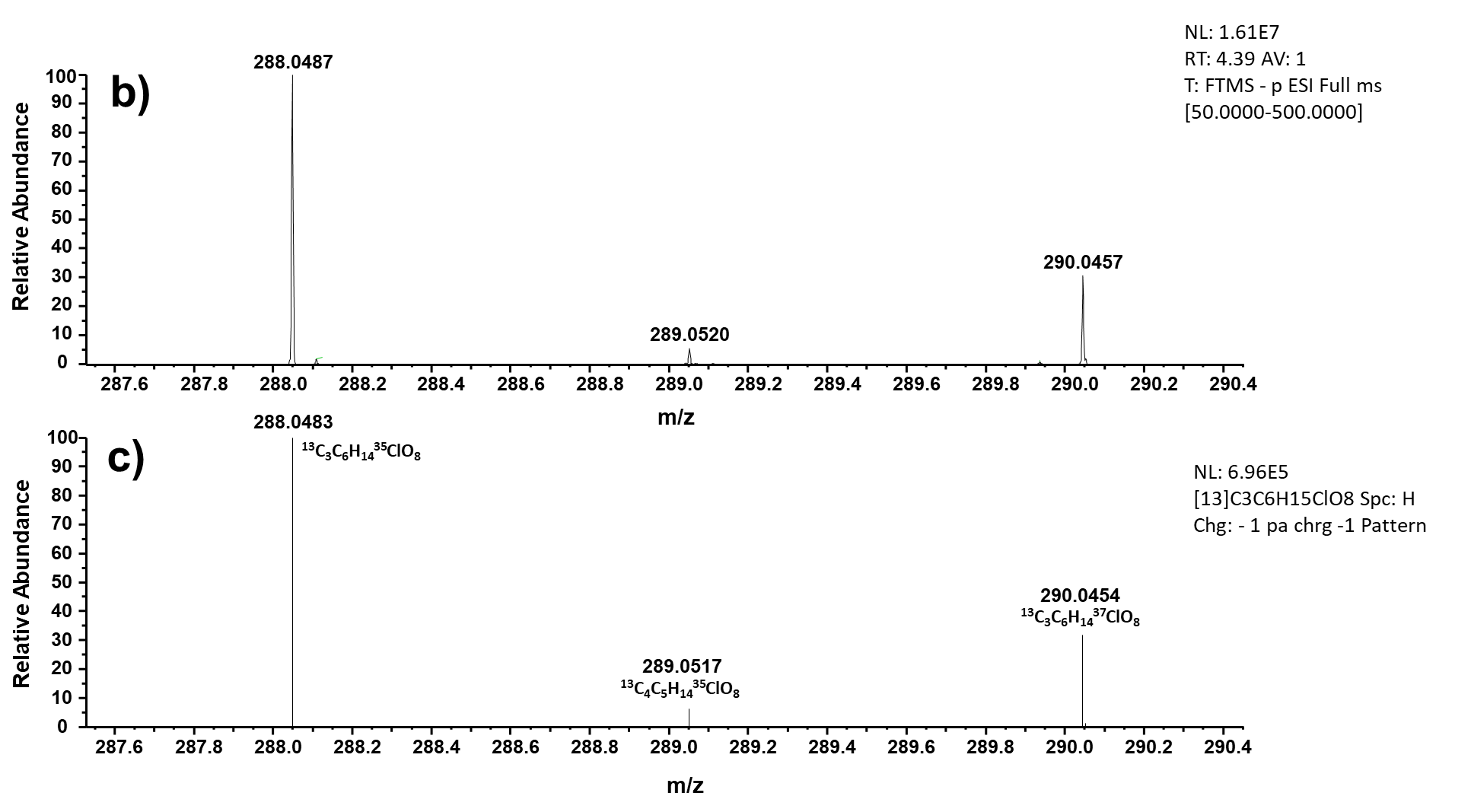 |
| 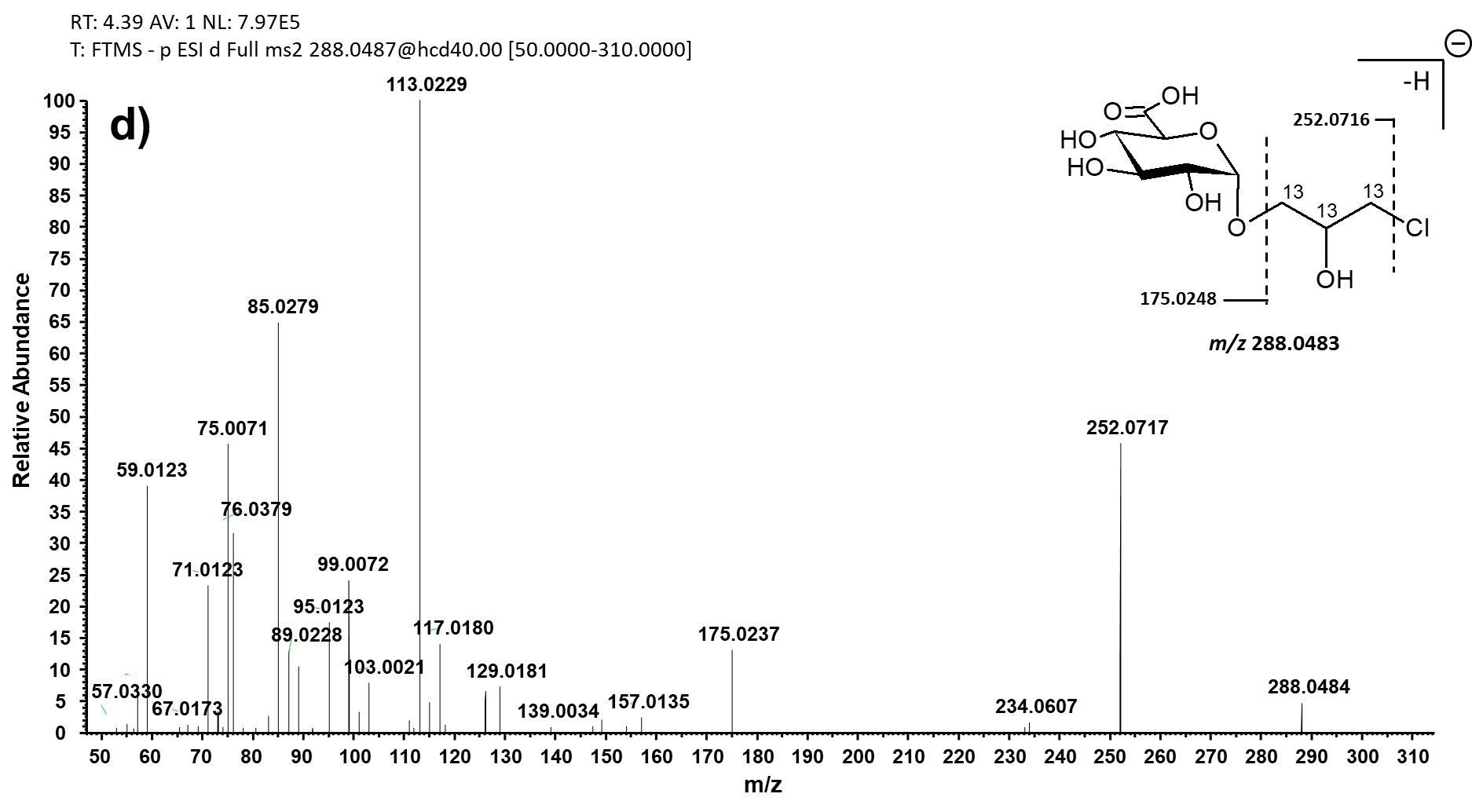 |
| **Figure S14**. HR-MS identification of [^13^C_3_]3-MCPD glucuronide: extracted ion chromatogramm (a), experimental (b) and theoretical (c) isotopic patterns, and MS2 spectrum (d). |

| 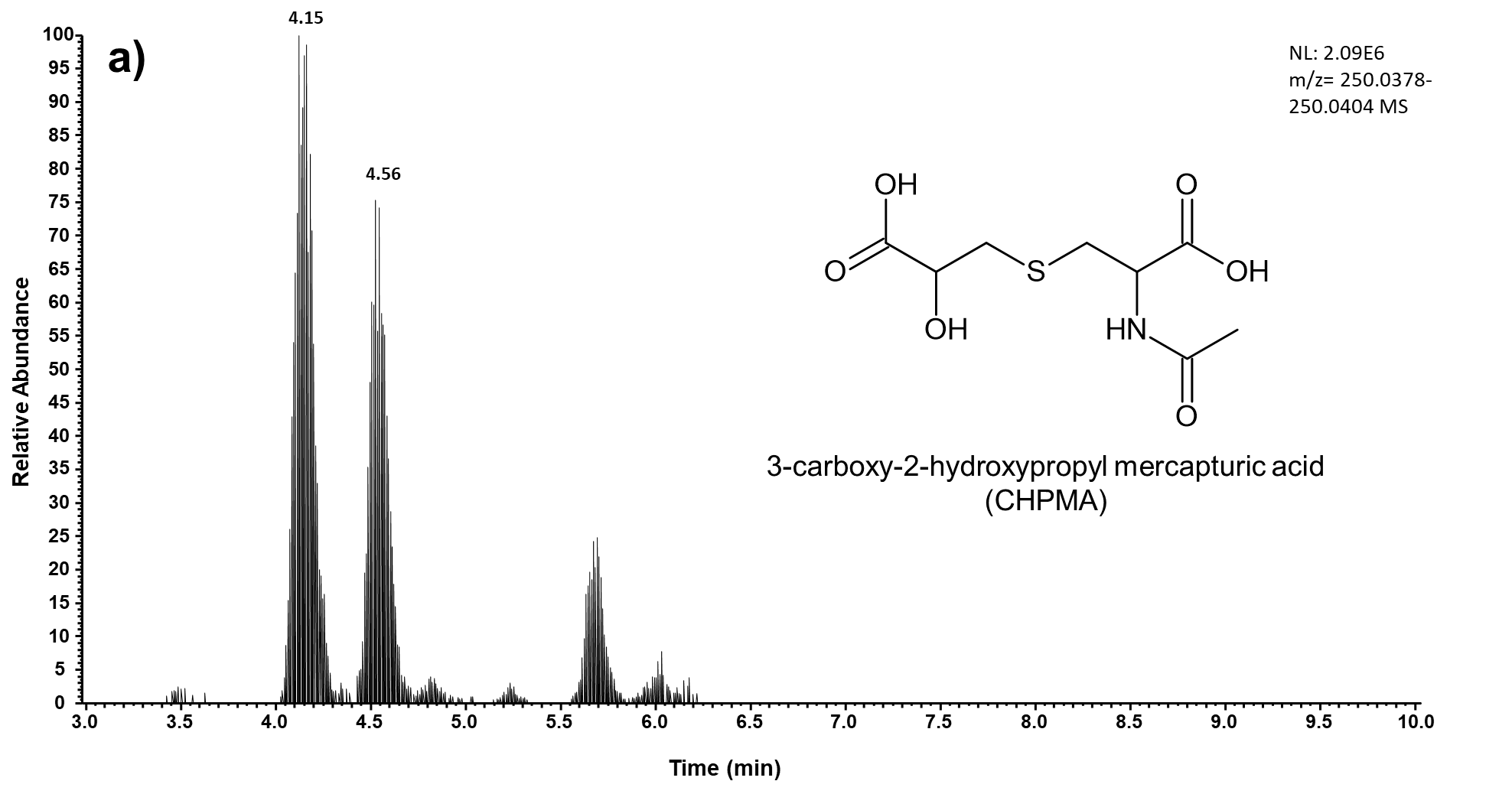 |
| --- |
| 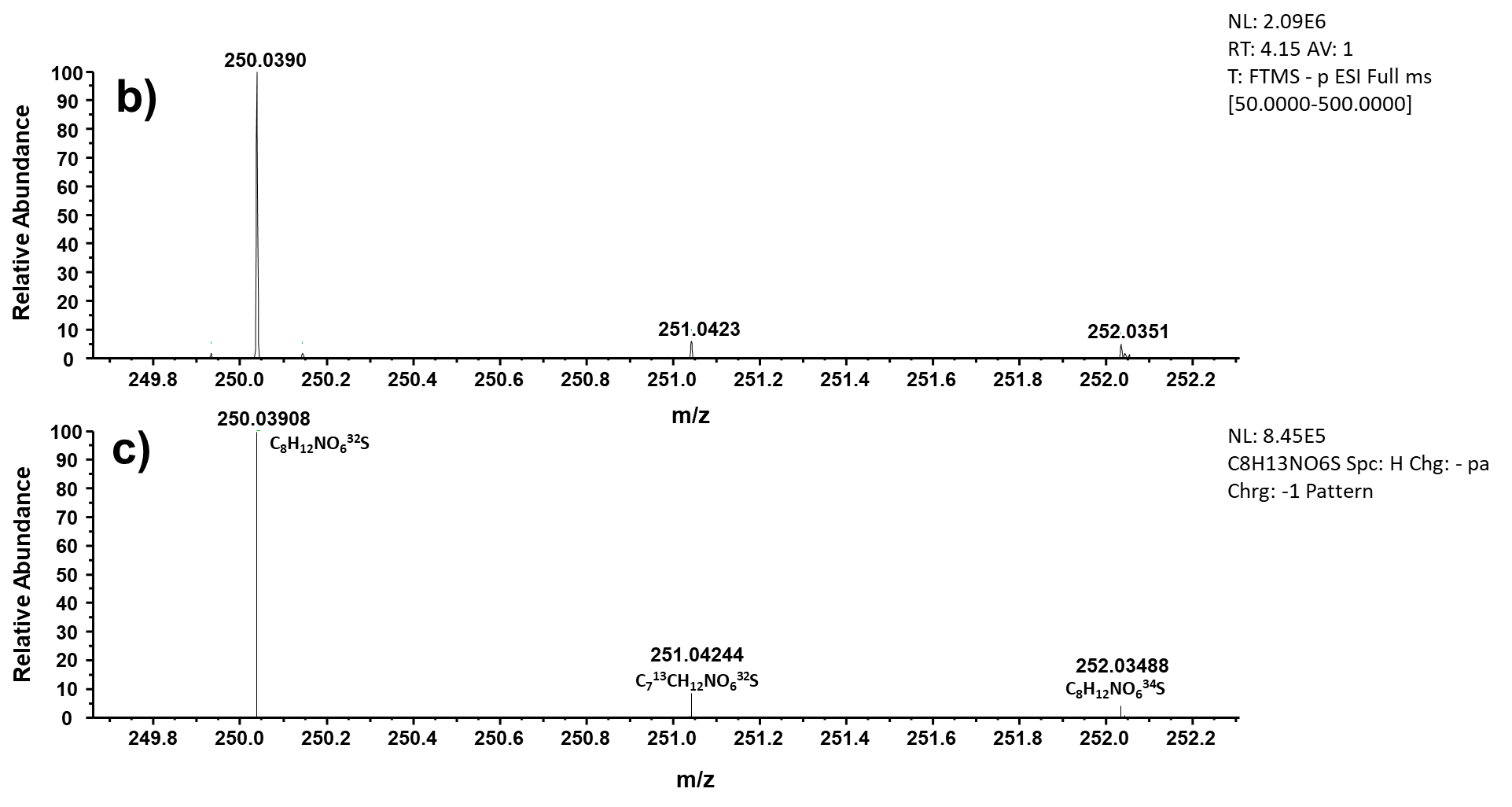 |
| 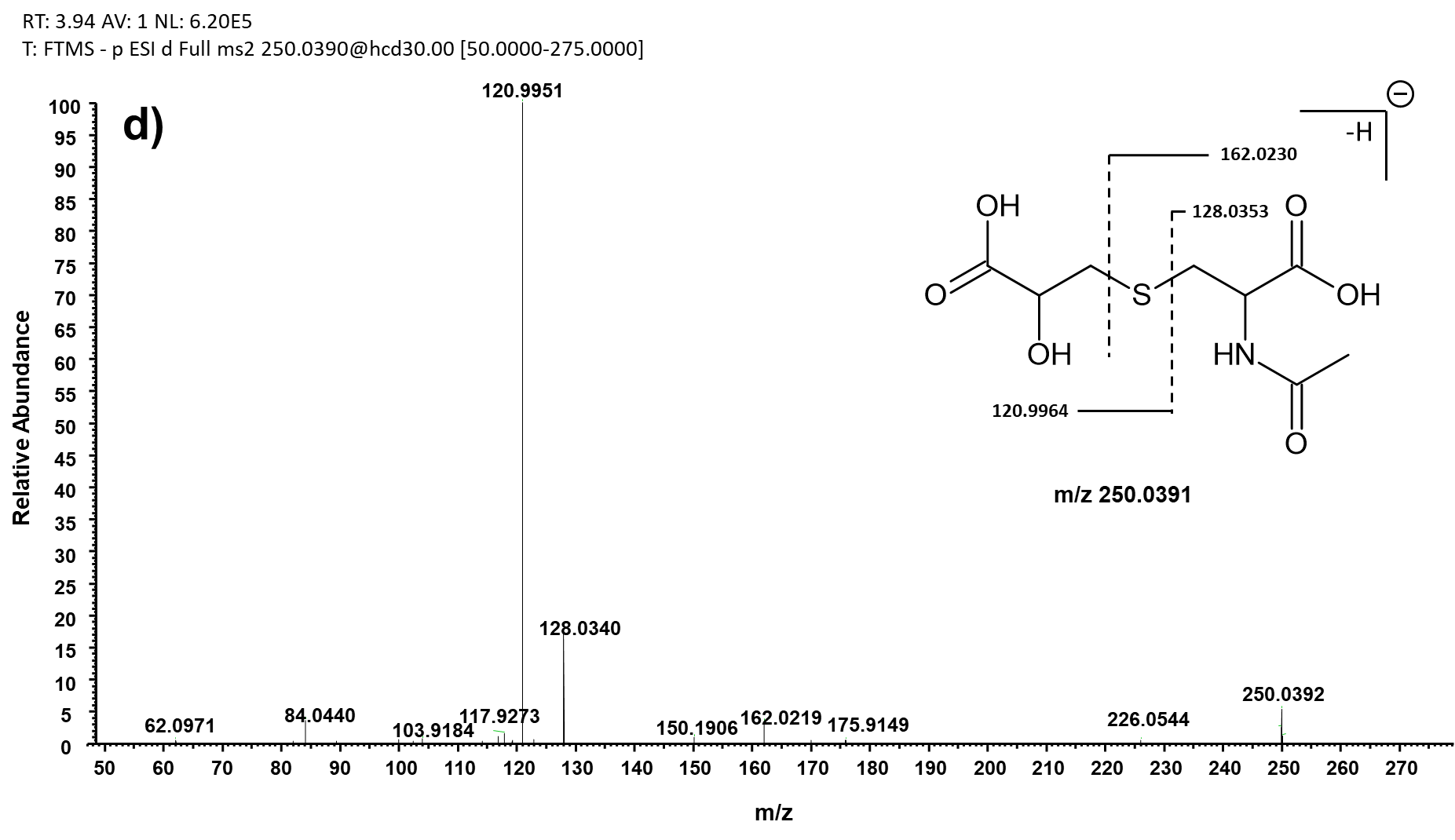 |
| **Figure S15**. HR-MS identification of 3-carboxy-2-hydroxypropyl mercapturic acid (CHPMA): extracted ion chromatogramm (a), experimental (b) and theoretical (c) isotopic patterns, and MS2 spectrum (d). |
| 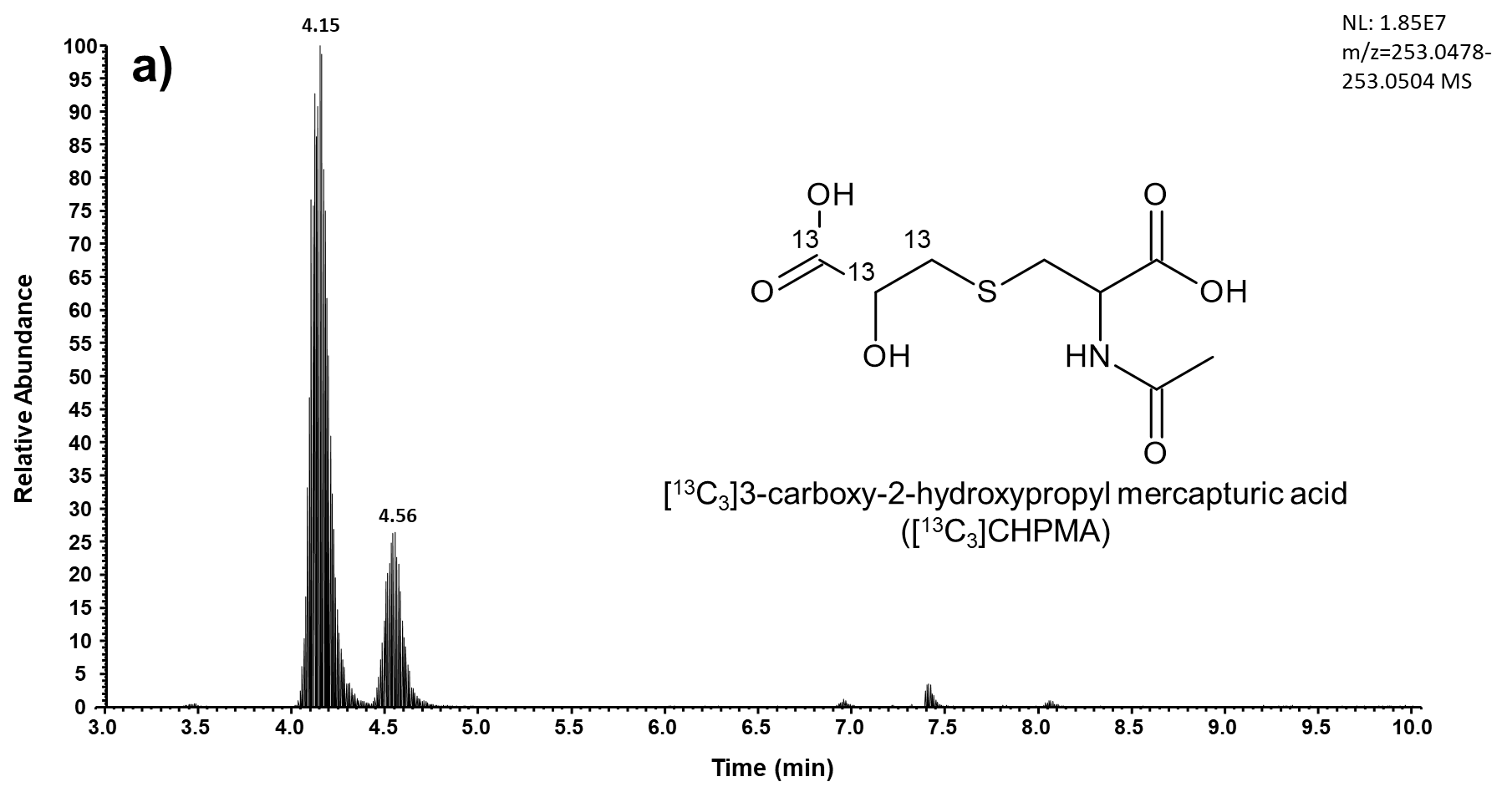 |
| 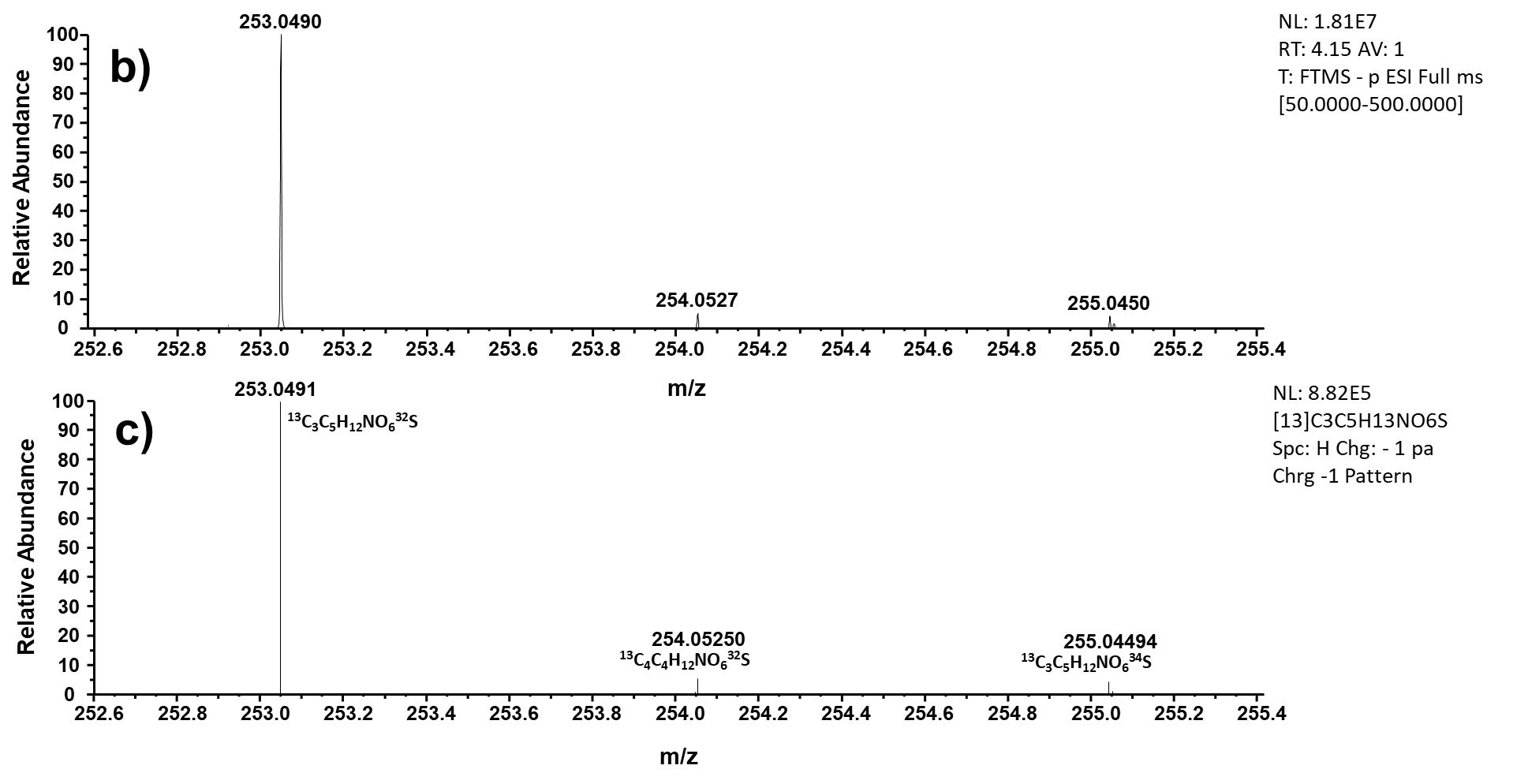 |
| 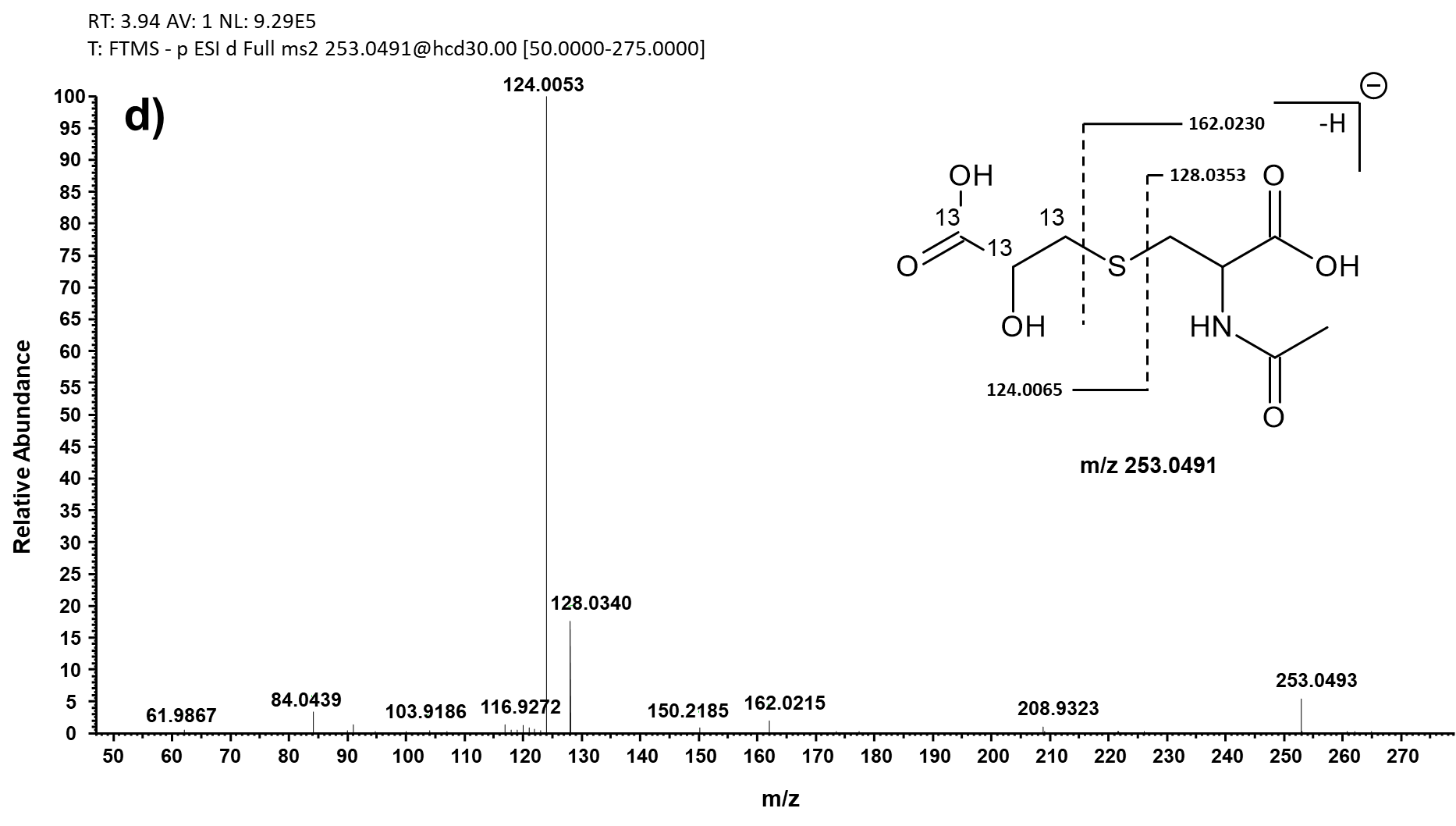 |
| **Figure S16**. HR-MS identification of [^13^C_3_]3-carboxy-2-hydroxypropyl mercapturic acid ([^13^C_3_]CHPMA): extracted ion chromatogramm (a), experimental (b) and theoretical (c) isotopic patterns, and MS2 spectrum (d). |
| 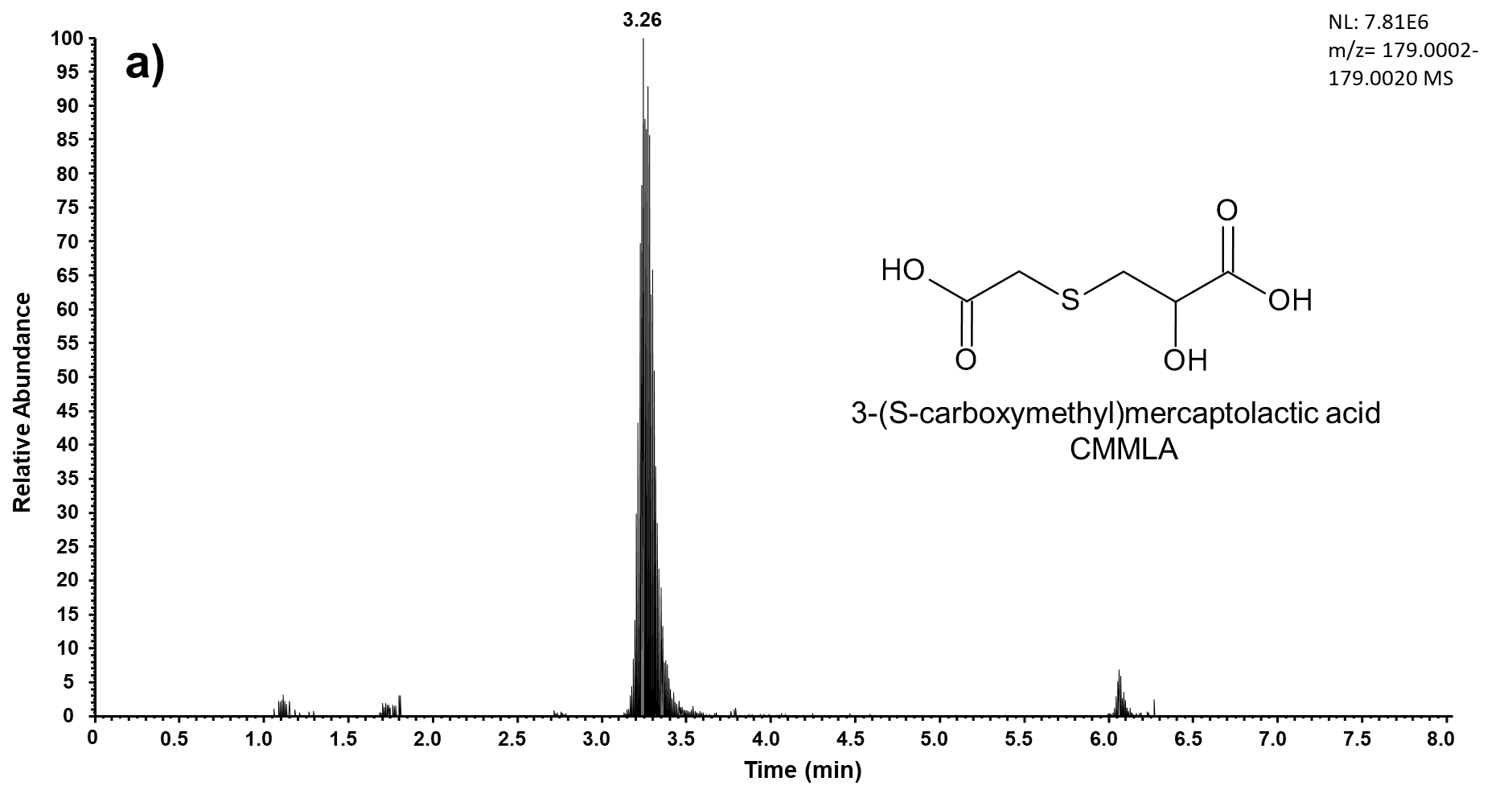 |
| 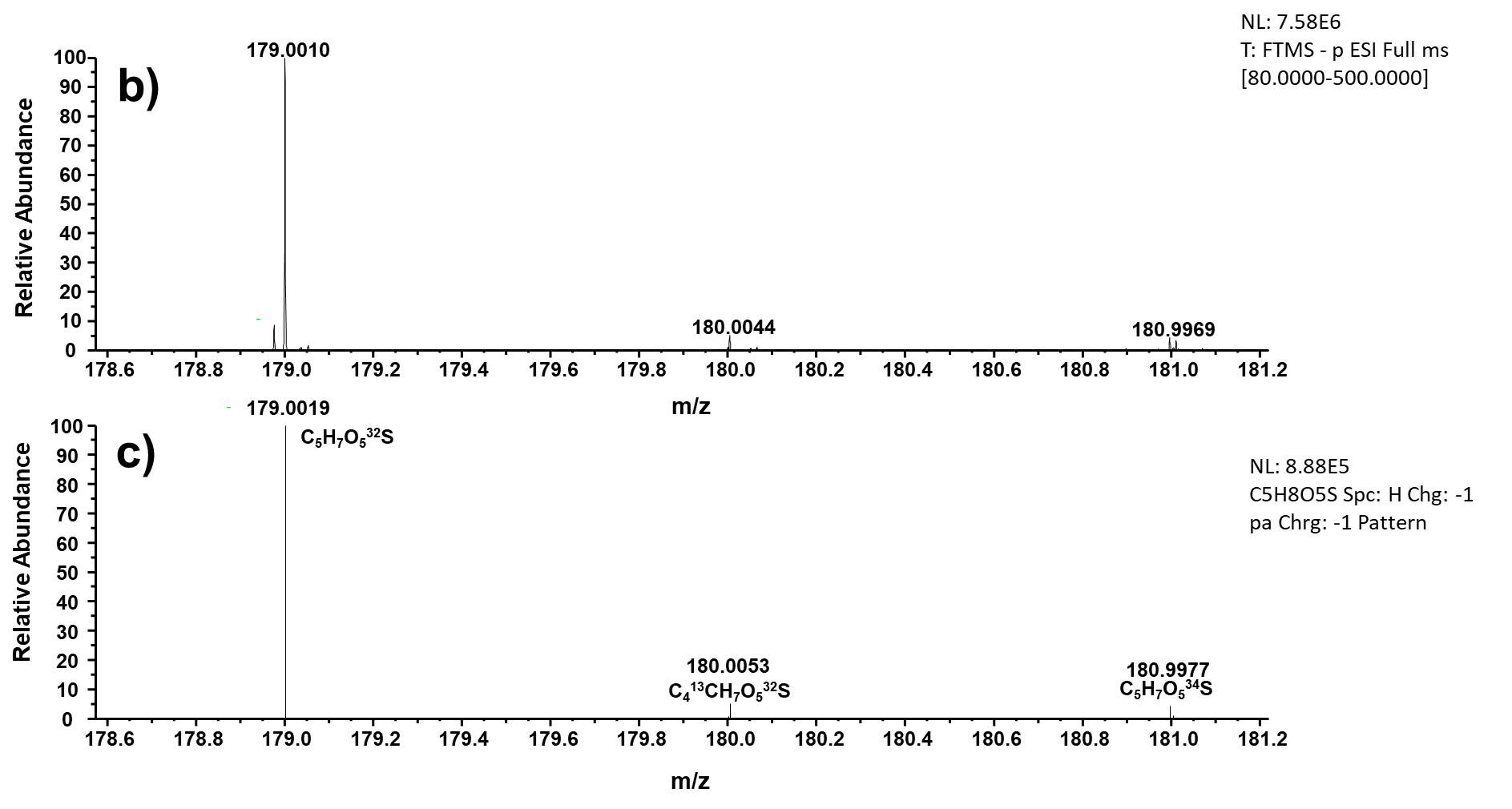 |
| 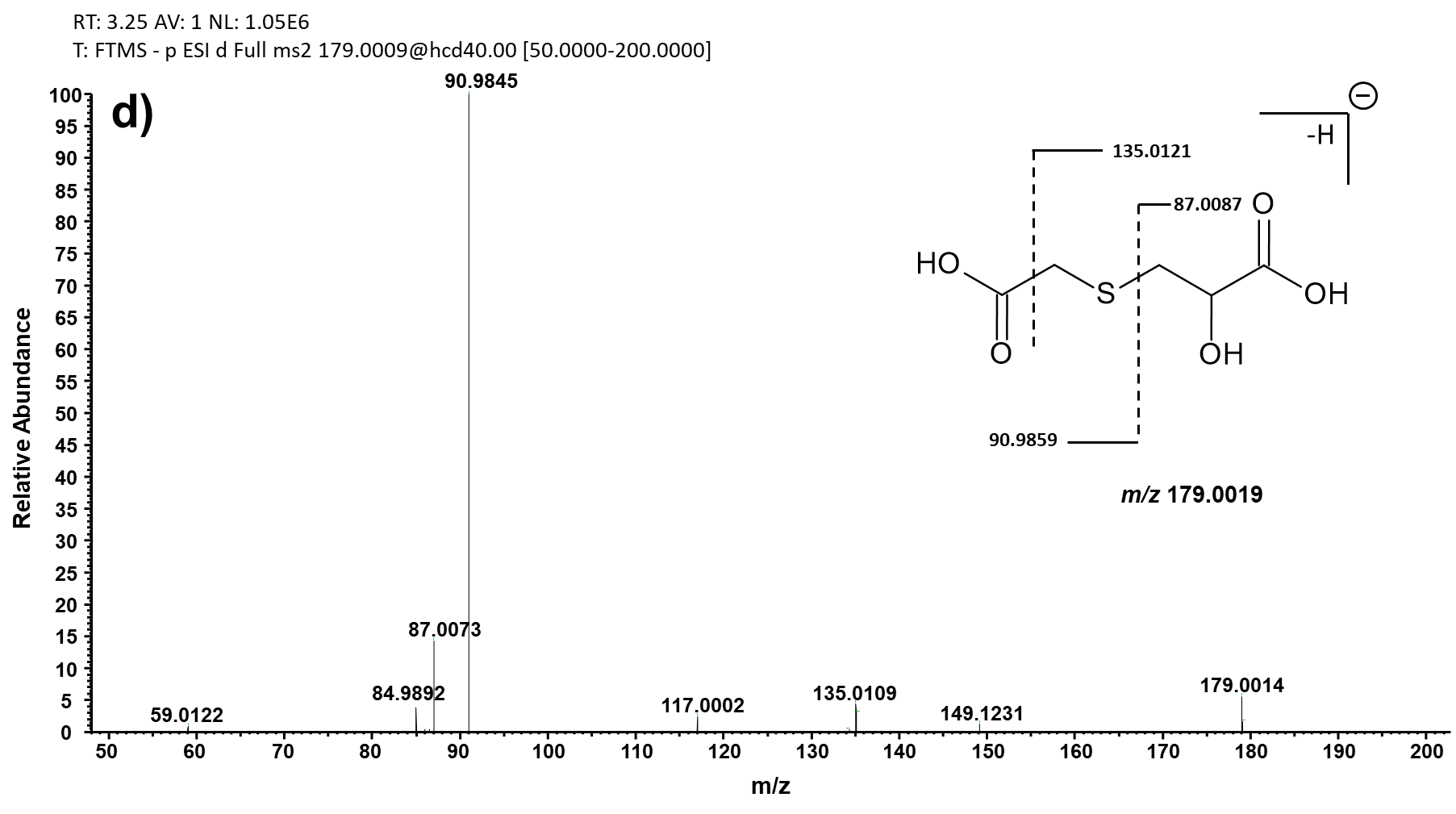 |
| **Figure S17**. HR-MS identification of 3-(S-carboxymethyl) mercaptolactic acid (CMMLA): extracted ion chromatogramm (a), experimental (b) and theoretical (c) isotopic patterns, and MS2 spectrum (d). |
| 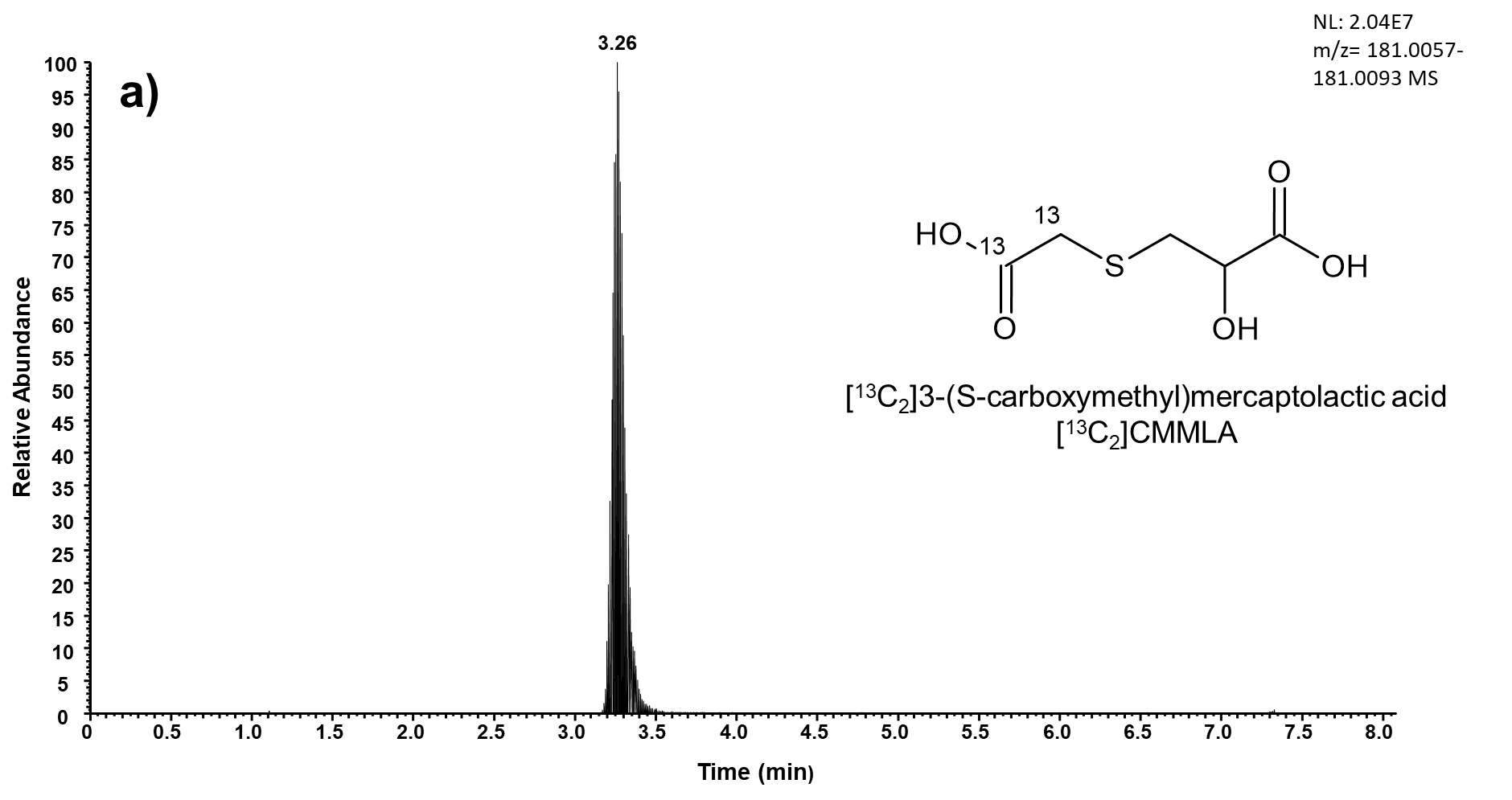 |
| 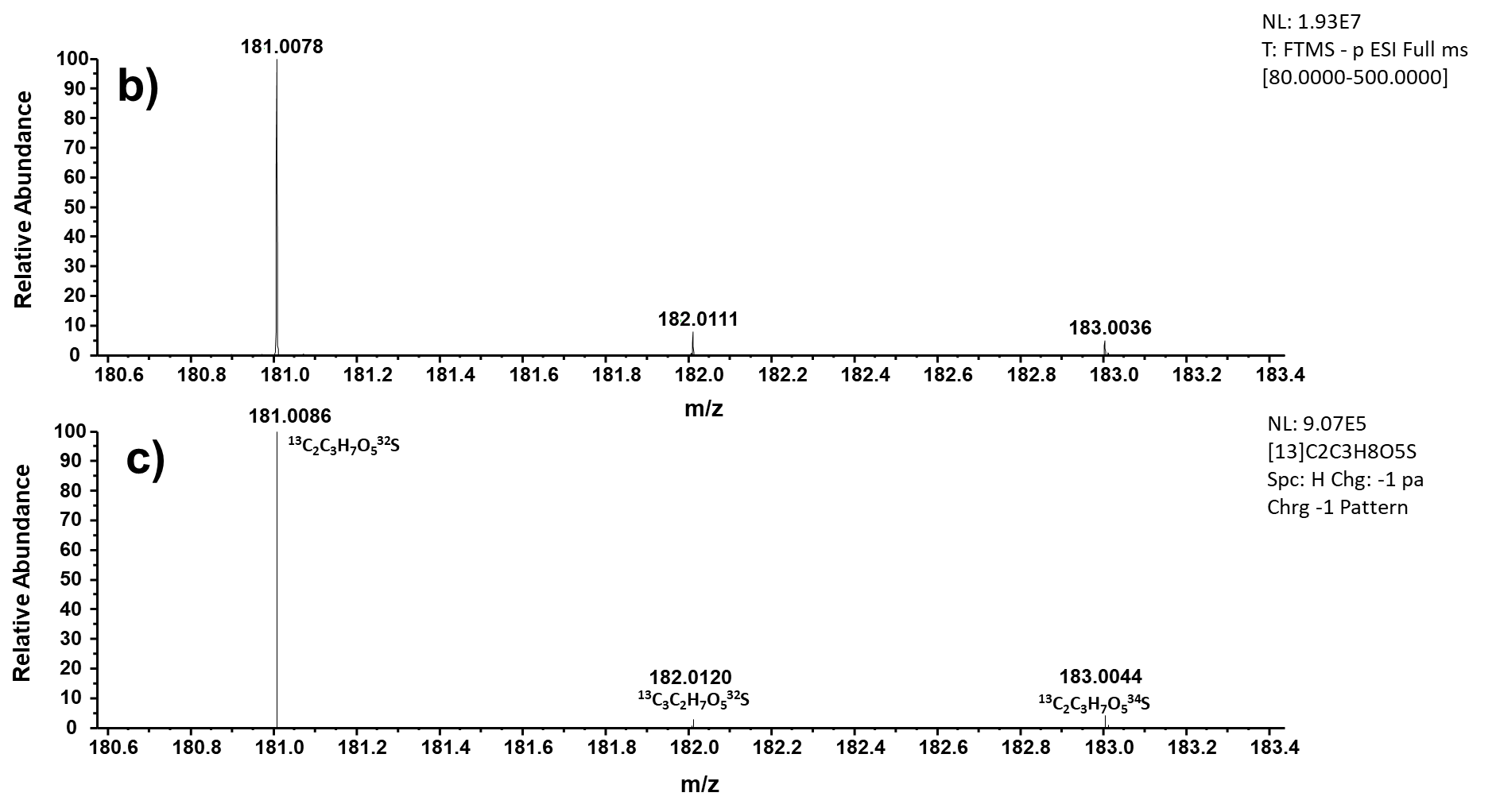 |
| 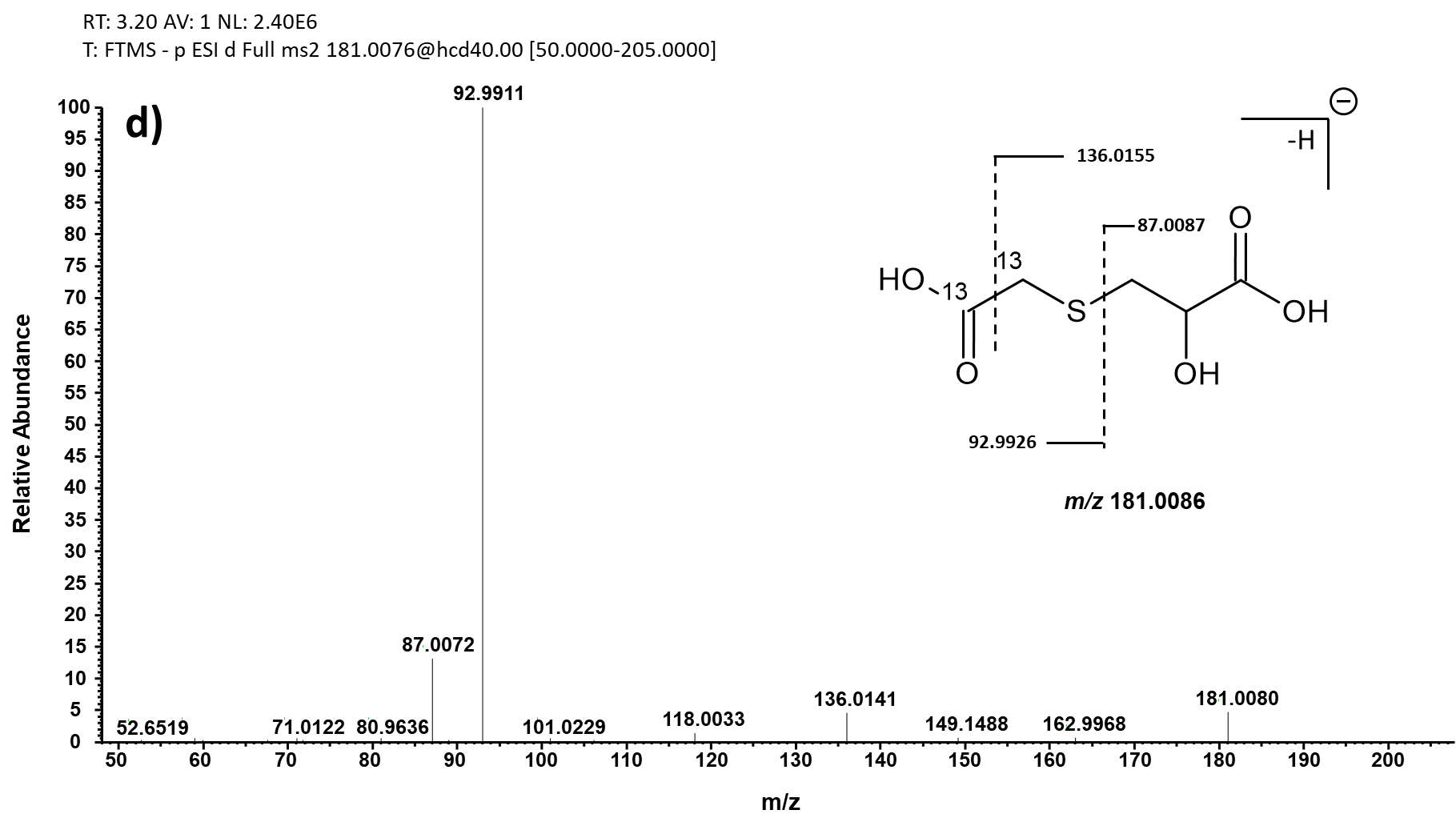 |
| **Figure S18**. HR-MS identification of [^13^C_2_]3-(S-carboxymethyl)mercaptolactic acid ([^13^C_2_]CMMLA): extracted ion chromatogramm (a), experimental (b) and theoretical (c) isotopic patterns, and MS2 spectrum (d). |
